# Mental health and psychosocial wellbeing among beneficiaries of a community-based intervention in Nepal: Findings from a cross-sectional endline evaluation of the PARBARDHAN project

**DOI:** 10.1371/journal.pgph.0006666

**Published:** 2026-10-16

**Authors:** Himal Gaire, Ganesh Bhandari, Rima Mishra, Ram Lal Shrestha, Aishwarya Prasad Dhakal, Pashupati Mahat

**Affiliations:** 1 Centre for Mental Health and Counselling Nepal (CMC-N), Kathmandu, Nepal; 2 School of Health and Allied Sciences, Pokhara University, Pokhara, Nepal; 3 Mid-Western University, Birendrangar, Nepal; ICDDR: International Centre for Diarrhoeal Disease Research Bangladesh, BANGLADESH

## Abstract

Mental health and psychosocial wellbeing among migrant workers and their families are important public health concerns in Nepal. The PARBARDHAN project used a community-based approach to strengthen mental health and psychosocial support, community participation, and local service capacity in migration-affected communities. This study describes psychosocial wellbeing at endline evaluation and compares findings descriptively with baseline and midline**.** A cross-sectional, mixed-methods endline evaluation was conducted in Salyan and Kailali districts of Nepal. The quantitative component included 245 project beneficiaries aged 16–80 yearsincluding20 (8.2%) migrant/returnee workers and 225 (91.8%) family/community respondents; 216 (88.2%) reported having a family member currently abroad. Psychological distress was assessed using the General Health Questionnaire (GHQ-12), and anxiety and depression the Hopkins Symptom Checklist (HSCL-25). Life satisfaction and subjective happiness were assessed. Qualitative interviews and focus group discussions provided contextual information on project implementation and perceived effects. Quantitative data were analysed using descriptive statistics while qualitative data were analysed using framework analysis. Data were collected from3-26 February, 2026. At endline, 34.7% screened positive for psychological distress, while 8.2% screened positive for anxiety and 5.3% for depression (5.3%). Compared with baseline and midline assessment rounds, anxiety and depression screening positivity was lower at endline, whereas probable psychological distress was higher. Qualitative findings indicated increased mental health awareness, greater willingness to seek support, perceived reductions in stigma, and support through self-help groups, peer groups counselling, and community activities. However, substantial dissatisfaction with life was also reported. Because the evaluation rounds used independent cross-sectional samples, the observed differences cannot be interpreted as within-person changes or attributed causally to the PARBARDHAN intervention. The findings indicate the perceived value of community-based mental health and psychosocial support while highlighting continuing economic insecurity, family separation, and other structural factors affecting psychosocial wellbeing.

## 1. Introduction

Nepal has an economy increasingly reliant on remittances generated by labour migration which has become an important livelihood strategy for many households. Globally, migrant workers constitute a substantial and growing component of the labour force, with approximately 164 million people worldwide classified as migrant workers in 2017 [1,2]. Nepal is one of the largest labor-sending nations in South Asia, supplying workers to countries across the region and beyond [3]. Although labor migration provides important economic benefits through employment and remittances, the migration process can also expose workers and their families to multiple social, occupational, economic, and relational stressors. Mental health should therefore be understood not only as an individual health outcome but also as a product of the conditions in which migration occurs and the social environments in which migrant workers and their families live [3,4].

Migrant workers may experience mental health difficulties throughout the migration cycle, including during recruitment, transit, employment, and return. Poor working and living conditions, occupational stress, social isolation, exploitation, limited legal protection, discrimination, disrupted social networks, and restricted access to healthcare have been identified as important psychosocial challenges [4–8]. Previous studies have consistently shown that migrant workers experience higher rates of mental health problems compared to native workers, highlighting the urgent need for attention to their mental health and psychosocial wellbeing [9,10]. A systematic review and meta-analysis by Hasan et al. (2021) found that approximately one in three migrant workers experienced a common mental health disorder, underscoring the scale of this global challenge [11]. In Nepal specifically, national survey data indicate that mental health problems are prevalent yet significantly underserved, particularly in rural and migration-affected communities [3,12]. Mental health problems among migrant workers therefore need to be understood not only as individual health conditions but also in relation to the broader social and structural circumstances surrounding migration.

The mental health and psychosocial wellbeing of family members left behind is an important but comparatively less explored dimension of labour migration. Separation from a migrant family member may alter household responsibilities, caregiving arrangements, emotional support, and family relationships. Although remittances may improve household economic conditions and provide opportunities for education and other forms of investment, the absence of a family member may also create emotional and social difficulties [5,6]. Systematic reviews have reported associations between parental or family migration and poorer mental health outcomes among children and adolescents left behind, including increased risks of depression and anxiety [13]. Evidence also suggests that the effects of international labour migration on left-behind children and their wellbeing are complex and may vary according to family circumstances, gender, age, duration of separation, and social support [14]. These findings highlight the importance of considering mental health and psychosocial wellbeing beyond migrant workers themselves and examining the broader family and community context in which migration occurs.

Mental health outcomes among migrant workers and their families are influenced by multiple interconnected psychosocial and structural determinants. These include family separation, economic insecurity, migration-related debt, employment uncertainty, poor working and living conditions, social isolation, discrimination, stigma, limited social support, and restricted access to appropriate health and psychosocial services [4–6,9]. The availability and accessibility of mental health services are particularly important in rural and resource-constrained settings, where shortages of trained personnel, limited service availability, stigma, and weak referral mechanisms can create substantial barriers to care [9,15]. In Nepal, persistent gaps in mental health service provision and integration into primary healthcare further reinforce the need for community-based approaches that can address both individual needs and the social determinants of mental health [15,16].

Community-based mental health and psychosocial support (MHPSS) approaches have increasingly been promoted as a means of improving access to care, reducing stigma, strengthening social support, and integrating mental health services within existing community and primary healthcare systems [15,17–21]. Task-sharing, community-based psychosocial interventions, peer support, self-help groups, and engagement of people with lived experience have demonstrated potential to expand mental health support, particularly in low-resource settings [17–19]. However, evidence regarding the effectiveness and sustainability of such approaches remains variable across settings, and implementation depends strongly on local health systems, community structures, available resources, and social context [18,21]. This suggests that community-based MHPSS interventions should be examined not only in terms of individual mental health outcomes but also in relation to social support, stigma, service access, inclusion, local capacity, and sustainability.

In response to these challenges, CMC-Nepal implemented the PARBARDHAN project; formally titled “Enhancing Mental Health and Psychosocial Wellbeing of Migrant Workers and their Families” (EMHPW-MW) in English, and known locally in Nepali communities as PARBARDHAN — from January 2022 to December 2025, with financial support from Felm. The project was implemented across seven municipalities in Salyan district (Sharada Municipality, Bangad Kupinde Municipality, Bagchaur Municipality, and Chattreshwori Rural Municipality) and Kailali district (Tikapur Municipality, Ghodaghodi Municipality, and Janaki Rural Municipality). Primary beneficiaries included migrant workers and their family members, persons with mental health conditions, persons with disabilities and other community stakeholders. The project adopted a community-based, rights-oriented approach with a strong focus on disability inclusion, local capacity strengthening, and integration of mental health services into primary healthcare. Key intervention strategies included mental health awareness campaigns, formation and mobilization of self-help groups (SHGs) and peer support networks, counseling services, capacity building of health workers, and engagement of local governments and health institutions to build sustainable support systems and reduce stigma. The baseline evaluation documented substantial mental health needs in the project communities with 20.4% of participants screeing positive for probable psychosocial distress,37.2% for anxiety, and 26.2% for depression [11]. The midline evaluation subsequently reported lower levels of anxiety and depression and higher levels of happiness and life satisfaction, alongside expansion of mental health services and continued challenges related to stigma, resources, and policy implementation.¹¹Despite these findings, important knowledge gaps remain regarding mental health and psychosocial wellbeing in communities affected by labour migration and the extent to which community-based MHPSS approaches are associated with changes in different dimensions of wellbeing. Previous evaluations provided evidence on psychological distress, anxiety, depression, happiness, life satisfaction, service access, stigma, social support, and community-level implementation, but further evidence was needed at the end of the project period to understand the status of these outcomes and the experiences of beneficiaries and stakeholders. The present endline evaluation therefore examined psychological distress, anxiety, depression, life satisfaction, and happiness, while the qualitative component explored perceived changes in mental health and psychosocial wellbeing, coping and social support, stigma, service access, community support mechanisms, and sustainability. Importantly, the baseline, midline, and endline evaluations were conducted using independent cross-sectional samples rather than following the same individuals over time. Accordingly, comparisons across evaluation rounds are descriptive and are intended to identify patterns and differences across project phases rather than to estimate within-person change or establish a causal effect of the PARBARDHAN intervention. By combining quantitative assessment with qualitative evidence, this study contributes to the limited evidence on community-based MHPSS in migration-affected communities in Nepal and identifies remaining gaps and priorities for strengthening sustainable, inclusive, and locally responsive mental health services.

## 2. Materials and methods

### 2.1 Ethical considerations

The study protocol was reviewed and approved by the Nepal Health Research Council (NHRC; Ref. 1088 approved on 13 Janaury 2026). Data Collection was carried out from 3-26 February 2026. Participation was entirely voluntary, and all eligible participants were informed about the study objectives, procedures, potential risks, and benefits before enrollment. Written informed consent was obtained from all adult participants. For participants below 18 years of age, parental or legal guardian consent was obtained and assent was sought from the participant, in accordance with the approved protocol. Participants were free to decline participation or withdraw at any stage without any penalty and without affecting their access to services, support, or routine care provided through the project or local health system. Confidentiality was maintained through anonymized coding, secure storage of data, and restricted access to study records.

### 2.2 Study design and attribution of effectiveness

This endline evaluation used a cross-sectional mixed methods design to assess the mental health and psychosocial wellbeing of participants and to document their experiences of mental health and psychosocial support within the PARBARDHAN project communities. The quantitative component consisted of a cross-sectional survey conducted among project beneficiaries and associated community stakeholders, while the qualitative component comprised Focus Group Discussions (FGDs) and Key Informant Interviews (KIIs) to explore experiences related to project relevance, implementation, perceived benefits, service access, stigma, coping, social support, and sustainability. Quantitative and qualitative findings were integrated during interpretation to provide complementary evidence on psychosocial outcomes and community-level experiences. The three evaluation phases were conducted using independent cross-sectional samples. Participants enrolled at baseline were not individually followed through midline or endline, and no participant-level identifiers were used to construct a longitudinal panel. Therefore, the study does not constitute a longitudinal cohort study, repeated-measures study, controlled before-and-after study, or quasi-experimental evaluation. There was no random allocation to intervention and control groups, no concurrent control or comparator group, and no individual-level follow-up across evaluation rounds.

For clarity, the evaluation phases corresponded to different assessment points within the PARBARDHAN project period. The baseline evaluation assessed the mental health and psychosocial situation before or at the early stage of project implementation, the midline evaluation assessed the situation during project implementation, and the endline evaluation assessed outcomes and experiences at the close of the project period. The baseline evaluation reported psychological distress, anxiety, and depression among the project population, while the midline evaluation reported mental health outcomes, happiness, life satisfaction, service-related experiences, and implementation-related findings [11,12]. The present endline evaluation collected new cross-sectional quantitative and qualitative data in February 2025. The sample sizes and participant composition differed across evaluation phases, and the populations represented by the three rounds were therefore overlapping but not identical.

Comparisons between baseline, midline, and endline findings were consequently undertaken descriptively to identify differences in observed proportions and patterns across evaluation points. For example, the endline prevalence estimates for psychological distress, anxiety, and depression were compared with the corresponding estimates previously reported at baseline and midline. These comparisons do not represent changes experienced by the same individuals and should not be interpreted as longitudinal change. Because the evaluation did not include randomization, a control group, individual follow-up, or a quasi-experimental design, observed differences between evaluation rounds cannot be attributed causally to the PARBARDHAN intervention.

Accordingly, the purpose of the endline evaluation was to describe the mental health and psychosocial wellbeing of the study population at endline, document participants’ and stakeholders’ experiences of the project, and compare available indicators descriptively with findings from earlier evaluation rounds. The study does not estimate a causal intervention effect. Where qualitative participants described perceived benefits associated with project activities, these accounts are presented as participants’ experiences and perceptions rather than as evidence of causal effectiveness. The analysis therefore distinguishes between observed differences across evaluation rounds and evidence of intervention effectiveness.

### 2.3 Study population, eligibility and sampling

The broader PARBARDHAN evaluation population comprised migrant workers and returnees, family members of migrant workers, persons with psychosocial or mental health conditions, persons with disabilities, self-help group (SHG) and peer-support group members, and relevant community and local-government stakeholders. Migrant workers and their families, persons with mental health conditions, and persons with disabilities were the primary beneficiary groups of the project. The broader evaluation therefore included both beneficiary populations and stakeholders involved in project implementation and community-level mental health and psychosocial support.

For the quantitative component, eligible participants were direct or indirect project beneficiaries aged 15 years or older, residing in one of the seven project municipalities and belonging to at least one defined beneficiary group. A total of 245 participants were enrolled using simple random sampling from the available project beneficiary lists.

The quantitative endline dataset was subsequently classified according to the respondent’s migration-related population status. Of the 245 respondents, 20 (8.2%) were migrant/returnee workers and 225 (91.8%) were family/community respondents. Thus, the quantitative sample predominantly represented family and community members associated with migrant-affected households rather than migrant workers themselves. In addition, 216 respondents (88.2%) reported having a family member currently abroad, while 29 (11.8%) reported no family member currently abroad. The latter group was retained because the project also included families of returnee migrants and other eligible project beneficiaries.

Disability and mental health status were treated as overlapping participant characteristics rather than mutually exclusive population categories. Seventy-four respondents (30.2%) were coded as having a disability and 68 (27.8%) reported a mental health problem. These characteristics could occur among both migrant/returnee workers and family/community respondents and were therefore not added to the primary population categories.

The sample size was calculated using the Cochran formula: n = Z²pq/d², where Z = 1.96, p = 13.2% (midline depression prevalence), q = 86.8%, and d = 0.05, yielding a minimum sample of 177. After adding 20% for potential non-response, the target sample was 212; ultimately, 245 participants were enrolled.

The qualitative component used purposive sampling to obtain perspectives from relevant beneficiary and stakeholder groups, including SHG and peer-support group members, local elected representatives, health officials, and health-facility personnel. Qualitative participants were analyzed separately from the quantitative survey sample and were not combined with the 245 respondents for quantitative outcome estimation.

The intervention comprised multiple components, including mental health awareness, psychosocial counselling, self-help and peer-support groups, referral mechanisms, health-worker capacity building, disability inclusion, and engagement of local governments and health institutions. Consequently, exposure to individual intervention components was heterogeneous, and the study did not assume that all respondents received the same intervention components or intensity.

Baseline, midline, and endline assessments used independent cross-sectional samples. Therefore, comparisons across evaluation phases are presented descriptively and are not interpreted as within-person changes over time.

### 2.4 Comparability of baseline, midline and endline samples

The baseline, midline, and endline evaluations were conducted as separate cross-sectional assessments using independent samples. Although the evaluations covered the same PARBARDHAN project area and broadly targeted the project’s beneficiary population, the participants recruited at each evaluation phase were not individually followed across rounds. Consequently, the three samples should not be considered a longitudinal panel, and differences in their characteristics may contribute to differences in observed mental health and psychosocial wellbeing indicators.

The evaluation phases were conducted within the same overall project geography, comprising seven municipalities in Salyan and Kailali districts. However, the individual sampling units differed across evaluation rounds. For the endline evaluation, a comprehensive beneficiary list was obtained from CMC-Nepal for each municipality, and participants were selected using a random number generator, with replacement of individuals who were unavailable or did not consent. The baseline and midline evaluations were also conducted within the PARBARDHAN project population, but the available evaluation documentation did not establish individual-level continuity of participants across the three rounds. Therefore, the samples are considered overlapping in terms of the project population and geographical setting but non-identical at the individual level.

The characteristics of the three evaluation samples were compared descriptively for variables for which information was available and sufficiently comparable across evaluation rounds, including sample size, age, sex, district, migration or family migration status, beneficiary category, socioeconomic characteristics, and disability status (Table 1). Where a variable was not collected, reported, or measured using sufficiently comparable definitions in an earlier evaluation, it was not treated as directly comparable. In particular, differences in the composition of migrant workers, left-behind family members, persons with psychosocial disabilities, and other beneficiary groups may affect the distribution of mental health outcomes observed at each assessment point.

**Table 1:** Characteristics and methodological comparability of the baseline, midline, and endline assessment samples.

Characteristic	Baseline	Midline	Endline
Assessment design	Cross-sectional	Cross-sectional	Cross-sectional
Assessment period	March 2022	Feb 2025	Feb 2025
Study area	Salyan and Kailali	Salyan and Kailali	Salyan and Kailali
Sampling approach	Random Sampling	Random selection from beneficiary list	Random selection from beneficiary list
Participants followed longitudinally	No	No	No
Sample size	210	91	245
Sex distribution	Not available in harmonized dataset	Not available	94.3% female, 5.7% male
Migration/family status	Not available in harmonized dataset	Not available	88.2% had a family member abroad

### 2.5 Data collection tools

#### 2.5.1 Quantitative data collection.

- General Health Questionnaire (GHQ-12): A 12-item tool to assess psychological distress in nepali language. The questionnaire was administered in Nepali language A score of 2 or above indicated psychological distress [17].
- Hopkins Symptom Checklist (HSCL-25): A 25-item tool to measure anxiety and depression symptoms using Nepali language. The HSCL-25 was scored according to its established scoring procedure. The anxiety and depression items were scored individually and/or summarized according to the scoring approach specified in the study protocol. A cutoff score of 1.78 or more indicated significant distress [18]
- Life Satisfaction Tool: A 6-item tool to assess satisfaction with various life domains using a 6-point Likert scale.
- Happiness Tool: A 25-item tool to measure subjective well-being using a 5- point Likert scale.
- Structured household questionnaire: Collected participants demographics, exposure to migration, household composition, disability, MHPSS access.

#### 2.5.2 Qualitative data collection.

In depth Interviews (IDIs) were conducted with local government officials (mayors, deputy mayors, health section heads). An interview guide was used for IDI. Focus Group Discussions (FGDs) were conducted with diverse community groups, including Self-Help Groups (SHGs), peer support groups. A semi-structured FGD discussion guide was used, covering topics including project relevance, perceived changes in mental health, service access, stigma, coping strategies, and sustainability. KIIs were conducted at municipal offices or health facility premises; FGDs were conducted at community gathering venues including SHG meeting spaces and community halls. Quantitative data were collected through face-to-face structured interviews at participants’ households by trained enumerators.

### 2.6 Data collection process

Data for the PARBARDHAN endline evaluation were collected over 15 days in Kailali and Salyan by four trained Research Assistants (two per district). Prior to fieldwork, they received structured training from CMC-Nepal on study objectives, ethics, digital tools (KoBoToolbox), standardized instruments (GHQ-12, HSCL-25, Happiness, Life Satisfaction), qualitative methods, and referral procedures. Data collection involved coordination with local stakeholders to ensure proper recruitment and reduce non-response. Fieldwork was closely supervised by the lead evaluator and CMC-Nepal staff through visits, real-time data checks, and daily debriefings to maintain quality and address challenges.

### 2.7 Data analysis

Quantitative data were analyzed using SPSS v26. Descriptive statistics were used to summarize participant characteristics and the distribution of mental health and psychosocial outcomes. Categorical variables were summarized using frequencies and percentages, while continuous variables were summarized using means and standard deviations or medians and interquartile ranges, as appropriate based on the distribution of the data.

#### 2.7.1 Qualitative data analysis.

Qualitative data from the key informant interviews (KIIs) and focus group discussions (FGDs) were analyzed using framework analysis with NVivo 12. The qualitative component comprised 14 KIIs with local government officials and health workers and seven FGDs with self-help group (SHG) and peer support group members across the seven project municipalities. The qualitative sample was purposively selected to capture perspectives from key stakeholder and beneficiary groups relevant to the evaluation objectives.

Audio recordings were transcribed verbatim in Nepali and subsequently translated into English by trained translators. Transcripts were anonymized and reviewed for completeness, accuracy, and contextual clarity before analysis. The qualitative analysis followed the five stages of framework analysis: familiarization with the data, identification and development of a thematic framework, indexing/coding, charting of coded data into the framework, and mapping and interpretation of relationships and patterns across themes.

The initial analytical framework was developed deductively from the evaluation objectives and the OECD-DAC evaluation criteria, including relevance, effectiveness, efficiency, impact, sustainability, and coherence, as well as the project’s focus on disability inclusion. The framework was subsequently refined inductively during repeated review of the transcripts to incorporate themes and concepts that emerged directly from participants’ accounts. Coding was conducted at the level of meaningful segments of text, and related codes were grouped into categories and subsequently organized into overarching themes and subthemes.

Two researchers independently coded a subset of transcripts to assess consistency in interpretation and application of the coding framework. Coding discrepancies or differences in interpretation were discussed between the researchers and resolved through consensus. The coding framework was reviewed and refined during the analysis process as new concepts emerged. The final coded framework was then used to compare patterns across respondent groups, municipalities, and evaluation domains.

Qualitative findings were triangulated across data sources and respondent groups, particularly by comparing perspectives from SHG and peer support group members with those of local government officials and health workers. The qualitative findings were also considered alongside the quantitative results during interpretation. This integration was used to identify areas of convergence and divergence between the quantitative estimates and participants’ reported experiences and to provide contextual explanations for unexpected or contrasting quantitative findings.

Credibility of the qualitative findings was strengthened through the use of trained research assistants, standardized interview and FGD guides, field supervision by the lead evaluator and CMC-Nepal monitoring staff, daily field debriefings, transcript review, independent coding of a subset of transcripts, consensus-based resolution of coding discrepancies, and triangulation across respondent groups and methods. The analysis was directed toward the evaluation questions and was not intended to generate statistically generalizable estimates.

## 3. Results

### 3.1 Quantitative results

#### 3.1.1 Population composition of the quantitative endline sample.

Among the 245 quantitative respondents, the mean age was 38.16 years (SD 12.12), and 231 (94.3%) were female. Based on the migration-related population classification in the endline dataset, 20 respondents (8.2%) were migrant/returnee workers and 225 (91.8%) were family/community respondents. In addition, 216 respondents (88.2%) reported having a family member currently abroad. The person abroad was most commonly a husband (126, 51.4%) or son/daughter (73, 29.7%). These findings indicate that the quantitative endline sample predominantly represents family and community members affected by labor migration rather than migrant workers themselves. The predominance of female respondents is also consistent with this population composition. This distinction is important when interpreting the findings, particularly because the sample provides limited representation of migrant workers and returnees themselves.

The quantitative endline sample comprised 245 respondents. Based on the migration-related respondent classification recorded in the endline dataset, 20 (8.2%) were migrant/returnee workers and 225 (91.8%) were family/community respondents. Thus, the quantitative findings predominantly represent family and community members associated with migrant-affected households rather than migrant workers themselves.

Overall, 216 respondents (88.2%) reported having a family member currently abroad, whereas 29 (11.8%) reported no family member currently abroad. Among respondents reporting a family member abroad, the person abroad was most commonly a husband (126, 51.4%) or son/daughter (73, 29.7%), followed by father/mother (2, 0.8%) and other relatives (15, 6.1%).

Disability and self-reported mental health problems were overlapping characteristics within the sample rather than mutually exclusive population categories. Seventy-four respondents (30.2%) were coded as having a disability and 68 (27.8%) reported a mental health problem. These characteristics could occur among both migrant/returnee workers and family/community respondents and were therefore analyzed as participant characteristics rather than separate population groups (Table 2).

**Table 2:** Background, socio-demographic and disability related characteristics of the participants. (N = 245).

Variable	Category	Frequency	Percentage
**Age**	Below 18	1	0.4
	18-30	74	30.2
	31-44	94	38.4
	≥45	76	31
Mean: 38.16 and Sd: ± 12.12 Range: 16–80			
**Gender**	Male	14	5.7
	Female	231	94.3
**District**	Kailai	105	42.9
	Salyan	140	57.1
**Municipality**	35 samples from all selected municipalities		
**Ethnicity**	Bhraman	21	8.6
	Chettri/Thakuri	99	40.4
	Janajati	77	31.4
	Dalit	48	19.6
**Religion**	Hindu	229	93.47
	Christian	13	5.31
	Others (Attamabiswasi, no belief, kuldevata)	3	1.22
**Education Level**	Literate (able to read and write)	91	37.14
	Primary Level	69	28.16
	Secondar level	48	19.59
	intermediate	33	13.47
	Bachelor level and above	4	1.63
**Marital status**	Married	233	95.1
	Unmarried	7	2.86
	Single or divorced	5	2.04
**Type of Marriage**	Arrange	103	42.04
	Love marriage	56	22.86
	Elopement	56	22.86
**Number of family members**	≤5	138	43.7
	>5	107	56.3
Mean	**5.69**		
**Type of family**	Nuclear	109	44.49
	Joint	118	48.16
	Extended	18	7.35
**Occupation**	Housewife	94	38.37
	Agriculture	90	36.73
	Business/self-employed	30	12.24
	Employed	21	8.57
	Daily allowance based	6	2.45
	Unemployed	4	1.63
**Family income**	Mean: 65196		
**Relationship with family member**	Good	187	76.33

The detailed population composition is presented in Table 3.

**Table 3:** Population composition and key beneficiary characteristics of the quantitative endline sample (N = 245).

Population characteristic	n	%
**Primary respondent classification**		
Migrant/returnee worker	20	8.2
Family/community respondent	225	91.8
**Migration-related household status**		
Respondent had a family member currently abroad	216	88.2
Respondent had no family member currently abroad	29	11.8
Relationship to family member abroad*		
Husband	126	51.4
Son/daughter	73	29.7
Father/mother	2	0.8
Other	15	6.1
**Overlapping beneficiary characteristics**		
Reported disability	74	30.2
No reported disability	171	69.8
Reported mental health problem	68	27.8
No reported mental health problem	177	72.2

#### 3.1.2 Relationship status and remittance-related information.

Table 2 presents information on family relationships, migration, and remittances. Most respondents reported good family relationships (76.3%), with smaller proportions describing them as normal (19.6%) or not good (3.2%). Among those with family abroad, husbands (51.4%) and children (29.7%) were the most common. Most households received remittances regularly (62.4%) or sometimes (20.8%), while a few did not (4.9%) due to unemployment, loss of contact, or other reasons.

#### 3.1.3 Mental health and disability related characteristics of the respondents.

According to Table 4, 27.8% reported mental health problems, mostly with onset over one year ago. First contact for care was primarily health posts (13.1%) or hospitals (4.9%), with few consulting traditional healers (0.4%). About 30.2% of them reported disabilities with the majority having mental disability (70.3). All participants (100%) reported no vision or hearing problems after using glasses and hearing device. Most could walk easily (97.3%), 70.4% had some concentration difficulty and 65.8% some self-care difficulty; severe difficulties were reported by 1.4% and 5.5%, respectively. Communication was the most affected, with 32.4% reporting slight difficulty, 35.1% severe difficulty, and only 28.3% experiencing no problems. Half felt respected (51.4%) sometimes, 36.5% sometimes, and 12.2% not at all (Table 5).

**Table 4:** Relationship status with family and remittance-related information.

Variable	Category	n/N	%
**Relationship with family member**	**Good**	**187/245**	**76.3**
	**Normal**	**48/245**	**19.6**
	**Not good**	**8/245**	**3.3**
	**Very bad**	**2/245**	**0.8**
**Relationship with family member currently abroad**	**Husband**	**126/216**	**58.3**
	**Son/daughter**	**73/216**	**33.8**
	**Father/mother**	**2/216**	**0.9**
	**Other**	**15/216**	**6.9**
**Remittance received**	**Yes/regularly**	**153/216**	**70.8**
	**Sometimes**	**51/216**	**23.6**
	**No**	**12/216**	**5.6**
**Reason for not receiving remittance**	**Unemployed**	**4/12**	**33.3**
	**Loss of contact**	**3/12**	**25.0**
	**Other**	**5/12**	**41.7**

**Table 5:** Mental health and disability related characteristics of the respondents.

Variable	Category	n/N	%
Self-reported mental health problem	Yes	68/245	27.8
	No	177/245	72.2
Onset of mental health problem	≤1 year	12/68	17.6
	1–5 years	27/68	39.7
	>5 years	29/68	42.6
First point of contact for mental health services	Basic health service centre/health post	32/68	47.1
	District hospital	12/68	17.6
	Private hospital	9/68	13.2
	Traditional healer	1/68	1.5
	Other	14/68	20.6
Disability reported	Yes	74/245	30.2
	No	171/245	69.8
Type of disability among those reporting disability	Physical	5/74	6.8
	Mental/psychosocial	52/74	70.3
	Vision-related	1/74	1.4
	Intellectual	16/74	21.6
Difficulty walking	No difficulty	72/74	97.3
	Some difficulty	2/74	2.7
Difficulty concentrating	No difficulty	23/74	31.1
	Some difficulty	50/74	67.6
	A lot of difficulty	1/74	1.4
Self-care difficulty	No difficulty	22/74	29.7
	Some difficulty	48/74	64.9
	A lot of difficulty	4/74	5.4
Communication difficulty	No difficulty	23/74	31.1
	Some difficulty	24/74	32.4
	A lot of difficulty	26/74	35.1
	Cannot do at all	1/74	1.4
Feeling respected by others	Not at all	9/74	12.2
	Sometimes	38/74	51.4
	Most of the time	27/74	36.5

#### 3.1.4 Psychological distress, anxiety, and depression among the respondents (n=245).

Among the 245 endline participants, 160 (65.3%) scored 0–2 on the GHQ-12, while 85 (34.7%) scored 3–12, indicating probable psychological distress. The observed prevalence of probable psychological distress at endline was 34.7%, compared with 20.4% at baseline and 20.9% at midline. For anxiety, the observed endline prevalence was 8.2% (20/245), compared with 37.2% at baseline and 20.9% at midline. Similarly, the observed prevalence of depression at endline was 5.3% (13/245), compared with 26.2% at baseline and 13.2% at midline. Because the three evaluation rounds used independent cross-sectional samples with differences in participant composition, these estimates represent descriptive differences between evaluation points rather than changes experienced by the same individuals. The differences should therefore not be interpreted as evidence of a causal intervention effect.

#### 3.1.5 Life satisfaction and happiness level among respondents.

Table 6 showed that among 245 participants, most reported low life satisfaction: 66.5% were dissatisfied, 15.9% slightly dissatisfied, and 6.1% extremely dissatisfied. Only a small proportion expressed satisfaction (2.0% satisfied, 5.7% slightly satisfied), while 3.7% were neutral. The Happiness Index showed that most were neutral (64.9%), about one-third were very happy (31%), and few were extremely happy (1.6%). Only a small minority reported being unhappy (2% slightly unhappy, 0.4% very unhappy) (Tables 7 and 8).

**Table 6:** Prevalence of psychological distress, anxiety and depression. (N = 245).

Prevalence of psychological distress (GHQ score)	n	%
Normal or no distress (GHQ = 0–2)	160	65.3
Probable distress (3–12)	85	34.7
**Prevalence of anxiety (HSCL-25, Cut-off ≥ 1.75)**		
Anxiety: Negative (<1.75)	225	91.8
Anxiety: Positive (≥1.75)	20	8.2
**Prevalence of depression (HSCL-25, Cut-off ≥ 1.78)**		
Depression: Negative (<1.78)	232	94.7
Depression: Positive (≥1.78)	13	5.3

**Table 7:** Three-phase comparison of key mental health outcome indicators (Baseline, Midline, Endline).

Indicator (Tool)	Baseline	Midline	Endline
Psychological distress (GHQ-12, cut-off ≥3)	20.4% (n = 245)	20.9% (n = 91)	34.7% (n = 245)
Anxiety (HSCL-25, cut-off ≥1.75)	37.2% (n = 245)	20.9% (n = 91)	8.2% (n = 245)
Depression (HSCL-25, cut-off ≥1.78)	26.2% (n = 245)	13.2% (n = 91)	5.3% (n = 245)

These three evaluation phases comprised independent cross-sectional samples from overlapping but non-identical populations. Comparisons are descriptive trend observations only; formal inferential testing is not appropriate given the independent sampling design. Baseline n = 245 refers to potential project beneficiaries; midline and endline n refer to confirmed project beneficiaries.

**Table 8:** Life satisfaction and happiness level among respondents.

Life satisfaction	Endline	Midline
	Endline (N = 245)	Midline (N = 68)
Extremely dissatisfied	15 (6.1)	2(2.9)
Dissatisfied	163 (66.5)	3(4.4)
Slightly dissatisfied	39 (15.9)	6(8.8)
Neutral	9 (3.7)	4(5.9)
Slightly satisfied	14 (5.7)	23(33.8)
Satisfied	5 (2.04)	24(35.3)
Extremely Satisfied	0(0)	6(8.8)
**Happiness level**		
Very unhappy (4–8)	1 (0.4)	0(0)
Slightly unhappy (9–13)	5 (2.0)	3(3.3)
Neutral (14–18)	159 (64.9)	48(52.7)
Very happy (19–23)	76 (31.0)	24(26.4)
Extremely happy (24–28)	4 (1.6)	15(16.5)

Baseline data for life satisfaction and happiness are not available for comparison, as these instruments were not administered during the baseline assessment. Comparisons are therefore limited to midline and endline assessments only.

#### 3.1.6 Overview of psychosocial outcome patterns across assessment phases.

Taken together, the available quantitative indicators showed heterogeneous patterns across domains of psychosocial wellbeing. The proportion of participants screening positive for anxiety and depression was lower at endline than at baseline and midline, whereas the proportion screening positive for probable psychological distress on the GHQ-12 was higher at endline. Life satisfaction also showed a different distribution at endline compared with midline, although these assessments involved different participants and substantially different sample sizes. Happiness showed a more mixed distribution across categories.

These findings do not support a single overall direction of change across all psychosocial outcomes. Rather, they indicate that different measures captured different dimensions of wellbeing and produced different patterns across independent assessment samples. The qualitative findings provided contextual information on social support, coping, mental health awareness, service access, economic insecurity, family separation, and employment uncertainty that may help interpret these heterogeneous quantitative patterns. These qualitative explanations are treated as participant-reported experiences and plausible contextual interpretations rather than demonstrated causal mechanisms.

### 3.2 Qualitative results

The qualitative component comprised 14 Key Informant Interviews (KIIs) with local government officials and health workers, and 7 Focus Group Discussions (FGDs) with SHG and peer support group members across the seven project municipalities. Framework analysis identified eight major themes: (1) Relevance of project activities, (2) Effectiveness of project activities, (3) Efficiency, (4) Impact of the project, (5) Sustainability, (6) Inclusion and equity, (7) Coherence, and (8) Future recommendations. Each theme is organised into subthemes below. Qualitative findings are interpreted in relation to the quantitative results and the broader evidence base in the Discussion section.

#### 3.2.1 Effectiveness of the project activities.

**Increased awareness and knowledge:** One of the clearest outcomes of the project was the transformation in community knowledge and perceptions about mental health. Participants consistently reported a shift away from traditional and harmful beliefs (e.g., viewing mental illness as madness, a curse, or spirit possession) toward understanding it as a health condition requiring treatment. This was facilitated through community awareness sessions, peer support group discussions, and public campaigns.

- *“Now, when we see such a person, we understand that they are experiencing mental health problems. There were many such people in our village.”* (FGD, Kupinde)
- *“Before, people thought it was possession by spirits, now they understand it is illness that needs treatment.”* (FGD, Kupinde)
- “Even family members started recognizing symptoms. They now come to us earlier, before the situation becomes worse.” (KII, Health Worker, Janaki)
- “Previously, villagers didn’t want to discuss mental health. After awareness initiatives, they started asking questions and seeking advice.” (IDI, Health Coordinator)

#### 3.2.2 Behavior change and reduced stigma.

Another strong outcome of the project was the reduction in stigma and discriminatory practices toward individuals with mental health conditions. Both community members and health workers observed a clear shift in how people with psychosocial disabilities were treated within households and society. Where previously families and communities practiced avoidance or exclusion, they are now increasingly inclusive and supportive.

- *“Now everyone eats together in the same place; they don’t say those who are ill should be separated.”* (FGD, Kupinde)
- *“Overall, this project has brought positive changes in people with mental health problems. We talk about counseling, basic income generation, and therapeutic services. Together, these three aspects help to reduce the problems*.”(IDI, Tikapur)
- *“Significant changes have occurred. Beneficiaries themselves say that they were previously in a disturbed state physically or mentally. After receiving support from the project, they have become mentally stronger and are now able to interact easily with their communities and families. Earlier, there were situations where families themselves discriminated against them, but now that is no longer the case”. (IDI, Ghodaghodi)*

#### 3.2.3 Emotional and social support.

Beyond formal health services, peer groups and community-based activities emerged as vital sources of emotional and social support. Group members consistently described these safe spaces as avenues for sharing personal struggles, reducing loneliness, and providing encouragement in times of crisis. Such peer-led support mechanisms contributed not only to improved well-being but also to suicide prevention within communities. Some participants also described situations in which individuals experiencing suicidal thoughts sought support from peers or group members. These accounts were presented as participants’ experiences and perceptions of the value of peer support rather than as evidence that the project directly prevented suicide attempts.

For example, one participant described contacting a peer-support member after experiencing suicidal thoughts and reported that the subsequent conversation was helpful. Another participant described providing support to a person experiencing suicidal feelings following migration-related family separation. These testimonies illustrate how participants perceived peer support as a source of immediate emotional assistance, but they do not establish a causal effect of the intervention on suicidal behavior.

- *“We go to the program, we listen to each other’s stories, and that makes us feel very good. Sharing your thoughts with a friend makes your heart feel lighter.”* (FGD, Kupinde)
- *“One of our sisters had suicidal thoughts, but after she read a pamphlet and called me, I counseled her and she decided to live.”* (FGD, Janaki)

#### 3.2.4 Perceived changes in mental health and functioning.

Participants described perceived improvements in emotional wellbeing, coping, social interaction, and daily functioning. Respondents associated these experiences with several forms of support, including psychosocial counselling, medication, peer support, self-help groups, and increased awareness of mental health.

These accounts provide qualitative evidence of participants’ experiences and perceptions of changes during the project period. They cannot, however, establish that the reported changes were caused by the PARBARDHAN intervention. The findings are therefore interpreted as perceived benefits and experiences rather than measured intervention effects.

**Improved mental health outcomes:** A key impact of the project was improved mental health outcomes among participants and their families. Respondents across IDIs and FGDs consistently highlighted noticeable changes in recovery, emotional regulation, and daily functioning. These improvements were attributed to increased access to counseling, medication, peer support, and awareness initiatives.

- *“There has been a change in cleanliness, and now we know that taking medicine helps to recover. We are now able to concentrate and work better.”* (FGD, Kupinde)
- *“Significant changes have occurred. Beneficiaries themselves say that they were previously in a disturbed state physically or mentally. After receiving support from the project, they have become mentally stronger and are now able to interact easily with their communities and families.”* (IDI, Ghodaghodi)

**Reduction in suicide risk:** Several participants described encounters with community members experiencing suicidal thoughts and reported that peer or community support provided an opportunity for these individuals to seek help. These accounts included experiences of counselling, informal conversations, referral to services, and encouragement from peers.

Participants perceived these interactions as helpful in supporting individuals during periods of emotional difficulty. However, the qualitative data did not prospectively measure suicidal behavior or establish whether an intervention prevented a suicide attempt. Accordingly, these accounts are presented as participant perceptions and experiences rather than evidence of a direct causal effect of the project on suicide prevention.

- *“One woman had suicidal thoughts, but after counseling and support, her health improved greatly.”* (FGD, Kupinde)
- *“We even advised patients who were having suicidal thoughts. After we talked with them, they said their mind felt lighter.”* (FGD, Janaki Municipality SHG)
- *“I counseled a sister who had suicidal feelings after her husband migrated abroad. She said later that our talk gave her the courage to live.”* (FGD, Peer Support Group Member, Ghodaghodi)
- *“While thinking and thinking, many thoughts come—even thoughts of suicide. That is why it is necessary to participate in such groups.”* (FGD, Kupinde)

**Smooth operation of self-help group:** Both local stakeholders and SHG members expressed confidence in their ability to sustain and operate the groups independently in the future. The groups have developed leadership capacity, internal trust, and established routines that make their continuity feasible even without intensive external support.

- *“Absolutely, they can operate independently. We have trained them in various stages on how to operate independently. We can continue to give them orientation and workshops to make them self-reliant. They may not need any external supporting organization in the future. They have also committed to operating independently after the project ends.”(IDI Health Coordinator)*
- *“We sit together, share our problems, and manage on our own. Even if the project ends, we can continue meeting.”* (FGD, Janaki Municipality SHG)
- *“We feel this program should have continued for some more years so we could learn more, but still, we are confident we can run the group.”* (FGD, Kupinde)
- *“The municipality and health posts know about our groups. If there is any problem, we can coordinate with them.”* (IDI, Municipal Representative)

#### 3.2.5 Inclusion & equity.

**Participation of marginalized groups:** Dalits, women, persons with disabilities, and Janajatis were actively included. Both IDI and FGD respondents emphasized that equal participation was encouraged, and deliberate efforts were made to invite those who were often left behind.

- *“Those who don’t have information, we call them and invite them. Dalits, women, and Janajatis all have equally received their rights.”* (FGD, Kupinde)
- *“In our group, there are Dalit members and also people with disabilities. Everyone is given the same chance to participate.”* (FGD, Janaki Municipality SHG)
- *“My son has a disability, but through this group we were supported and never excluded.”* (FGD, Janaki Municipality SHG)
- *“Services are being provided equally to everyone.”* (FGD, Kupinde)

Marginalized communities, who often face layered stigma and exclusion, were deliberately targeted for inclusion in awareness programs, counseling, and self-help groups.

**Disability inclusion:** Respondents shared that **access to disability allowances and services had begun**, and awareness had reduced negative labeling. However, challenges persisted in obtaining formal certification for psychosocial disabilities, which limited access to certain government benefits.

- *“The identity card for psychosocial disability has not come yet, but treatment is happening.”* (FGD, Kupinde)
- *“Compared to before, people with mental illness used to be called mad. Now everyone understands they need hospital treatment.”* (FGD, Kupinde)
- *“For physical disabilities, the allowance is provided. But for mental health problems, the government process is still complicated.”* (KII, Health Facility Incharge, Janaki)

### 3.3 Integration of quantitative and qualitative findings

Integration of the quantitative and qualitative findings showed both convergence and divergence across key domains of psychosocial wellbeing. Quantitative findings indicated lower prevalence of anxiety and depression at endline compared with baseline and midline estimates. Qualitative participants similarly described improved coping, stronger social support, greater confidence in discussing mental health concerns, and benefits from self-help groups, peer support networks, psychosocial counselling, and awareness activities. These findings provided contextual support for the observed pattern of lower anxiety and depression. At the same time, the quantitative findings showed a different pattern for psychological distress, which was higher at endline than at baseline and midline. Qualitative findings helped contextualize this discordance. Participants described continuing economic hardship, uncertainty regarding employment and income, family separation, and other migration-related stressors that could continue to contribute to broader psychological distress despite improvements in specific anxiety and depressive symptoms. Participants also described greater awareness and openness regarding mental health, which may have influenced recognition and reporting of psychological difficulties. These qualitative explanations should be considered contextual interpretations rather than evidence of causality. The findings on life satisfaction similarly illustrated the value of integrating the two strands. While the quantitative results indicated a decline in reported life satisfaction between midline and endline, qualitative participants frequently discussed financial insecurity, debt, employment uncertainty, and concerns about future livelihoods. Thus, the qualitative findings provided a plausible contextual explanation for the quantitative pattern and highlighted structural determinants of wellbeing that may not be captured by symptom-based screening measures. Overall, the qualitative evidence complemented the quantitative findings by providing explanations for observed patterns and highlighting contextual factors that shaped psychosocial wellbeing. Where findings were discordant, the evaluation retained the differences and considered possible explanations rather than treating the qualitative findings as confirmation of the quantitative results. Given the independent cross-sectional samples and descriptive nature of the quantitative analysis, the integrated findings are interpreted as complementary evidence about patterns and experiences rather than evidence of causal intervention effects.

## 4. Discussion

This endline evaluation of the PARBARDHAN project provides evidence of meaningful psychosocial improvements among migrant workers and their left-behind families in Salyan and Kailali districts of Nepal over the project period (2022–2025). The findings reveal a complex and nuanced picture: while anxiety and depression declined substantially from baseline levels, psychological distress increased, and life satisfaction deteriorated markedly by endline. These contrasting patterns reflect both the achievements and the limitations of community-based MHPSS interventions in addressing the multi-layered determinants of psychosocial wellbeing in migration-affected communities. The following discussion situates these findings within the broader regional and international evidence base and critically examines their implications for policy and practice.

An important consideration in interpreting the endline findings is the composition of the evaluation population. The quantitative assessment included participants with different relationships to labor migration and different psychosocial and disability-related characteristics. Most participants were connected to migrant-worker households, with 216 (88.2%) reporting a family member abroad, while 29 (11.8%) reported no family member abroad. In addition, 74 participants (30.2%) were recorded as having a disability and 68 (27.8%) reported previous mental health problems. These characteristics were not mutually exclusive and therefore should not be interpreted as separate population groups. The inclusion of these participants reflects the community- and household-oriented design of PARBARDHAN, which addressed mental health and psychosocial wellbeing not only among migrant workers but also among family members and other community members affected by migration and psychosocial vulnerabilities. The quantitative outcomes were therefore interpreted at the level of the overall evaluation sample rather than as evidence of intervention effects in a single homogeneous migrant-worker population. This distinction is important because the evaluation used independent cross-sectional samples and the composition of participants varied across the evaluation rounds. Accordingly, the findings primarily describe the psychosocial wellbeing of people reached by or connected to the intervention at endline and should not be interpreted as estimates of change among individual migrant workers over time. The observed differences in mental health indicators across baseline, midline, and endline should be interpreted cautiously because the three evaluation rounds used independent cross-sectional samples. Although the assessments were conducted within the same PARBARDHAN project geography and broadly targeted the project beneficiary population, individual participants were not followed across rounds. The composition of the samples also differed, and the endline sample was particularly dominated by women (94.3%) and left-behind family members, with 88.2% reporting a family member currently working abroad. Therefore, differences in observed prevalence may reflect variation in sex, age, migration status, beneficiary category, district or municipality, socioeconomic circumstances, disability status, participant selection, temporal conditions, or other unmeasured characteristics, in addition to any possible influence of project activities. Differences in measurement procedures or availability of indicators across evaluation rounds may also contribute to apparent differences. Consequently, the lower observed prevalence of anxiety and depression at endline cannot be interpreted as evidence that these outcomes improved within individuals or that the PARBARDHAN intervention caused the observed differences. The findings are more appropriately understood as descriptive contrasts between populations assessed at different stages of the project.

One of the most notable findings of this evaluation is the lower prevalence of anxiety and depression at endline relative to earlier evaluation phases. Respondents reported improved emotional regulation, resilience, and overall wellbeing. These reductions are clinically meaningful and compare favorably with findings from similar community-based MHPSS interventions in low- and middle-income country (LMIC) settings. A systematic review by Hoeft et al. (2018) found that task-shifted, community-based mental health interventions in LMICs consistently reduced depression and anxiety symptoms, with effect sizes comparable to those achieved in specialist settings [19]. Similarly, a randomized controlled trial of a community-based psychosocial intervention in Nepal by Jordans et al. (2010) demonstrated significant reductions in depression and anxiety among conflict-affected populations through structured psychosocial support; a design closely paralleling the PARBARDHAN approach [20]. The higher proportion screening positive for probable psychological distress on the GHQ-12 presents a contrasting finding. This does not necessarily contradict the lower HSCL-25 anxiety and depression estimates because the instruments assess related but distinct dimensions of psychological wellbeing. The GHQ-12 is a broader measure of general psychological distress, whereas the HSCL-25 anxiety and depression subscales focus on more specific symptom domains. Consequently, participants may experience substantial general distress without exceeding the specific HSCL-25 thresholds for anxiety or depression.

Qualitative findings align with this trend. Respondents consistently reported improved mental health, attributing it to psychosocial counseling, awareness programs, and peer support groups. The midline evaluation similarly reported lower anxiety and depression prevalence than baseline, suggesting that these differences were already evident early in the intervention period and remained evident at project completion; however, because each phase drew an independent sample, this pattern cannot be interpreted as change sustained within the same individuals [12] While the observed improvements cannot be exclusively attributed to project interventions given the cross-sectional, non-randomized design, the convergence of quantitative trends and qualitative narratives provides strong programmatic evidence of the project’s contributory role. Previous studies in Nepal and other low-resource settings have shown that community-based psychosocial interventions and mhGAP training significantly improve detection and management of common mental disorders, leading to reduced psychological distress [21,22].

The findings on life satisfaction further demonstrate why mental health outcomes should not be interpreted using symptom-based indicators alone. At midline, a substantial proportion of respondents reported some level of satisfaction with life, whereas the endline distribution was considerably more concentrated in the dissatisfied categories. However, the midline life-satisfaction assessment included only 68 participants compared with 245 at endline, and the participants were not followed longitudinally. The apparent difference therefore cannot be interpreted as evidence that individual participants experienced deterioration in life satisfaction.

Nevertheless, the finding is important because life satisfaction captures a broader evaluation of one’s circumstances than anxiety or depression screening alone. A participant may have access to counselling and experience fewer anxiety or depressive symptoms while continuing to be dissatisfied with household income, employment opportunities, debt, family separation, or future prospects. This distinction may help explain why improvements in specific symptom domains can coexist with continuing concerns about overall wellbeing. The qualitative findings support this interpretation. Participants discussed livelihood uncertainty, unstable income, employment concerns, and the continuing consequences of migration. These accounts are consistent with literature describing the psychosocial consequences of labour migration for both migrants and their families.1,3,5,6 They also suggest that improvements in mental health service access cannot by themselves resolve socioeconomic factors that influence people’s broader assessment of their lives.

The findings therefore argue for a multidimensional understanding of psychosocial wellbeing in migration-affected communities. Anxiety, depression, general psychological distress, life satisfaction, happiness, social support, and social inclusion represent related but non-identical domains. An intervention may perform differently across these domains, and improvement in one domain should not automatically be interpreted as overall improvement in wellbeing.

The project was also highly relevant to community priorities. Migrant workers and their families often burdened by separation, uncertainty, and financial strain—described the interventions as both timely and essential. Health workers highlighted that the project enabled them to identify individuals who were previously hidden due to stigma and connect them with care, directly addressing a critical service gap. The cultural acceptability of the strategies, such as peer support groups, SHGs, and local awareness campaigns, further reinforced their relevance. These interventions aligned with values of solidarity, collective responsibility, and mutual support, helping normalize conversations about mental health and reduce resistance to participation.

The project also contributed to increased availability and utilization of mental health services. At baseline, access to MHPSS services was very limited, with only 4 health facilities providing such support and from 4 to 19 health facilities by midline. By endline, this number had expanded to 29 health facilities, reflecting a significant scale-up in service availability alongside enhanced referral mechanisms. Trained personnel were able to deliver quality services at primary health facilities, which improved trust and service uptake within communities. Qualitative findings supported these achievements: participants expressed satisfaction with improved access, particularly mentioning the availability of medicines at municipal level and trained counselors at health facilities.

Qualitative findings indicate that stigma surrounding mental health has considerably reduced in the intervention areas. Respondents described a shift from harmful beliefs such as associating mental illness with spirit possession or weakness to greater recognition of mental health conditions as treatable. Families that previously excluded or discriminated against persons with psychosocial disabilities were increasingly supportive and inclusive. These changes reflect the effectiveness of targeted awareness campaigns, peer learning, and SHG activities in shifting social norms. Yet both findings reveal that stigma remains a significant barrier to mental health and well-being. Quantitatively, 11.8–16.7% of respondents reported feeling ‘not at all respected,’ while the majority indicated they were respected only “sometimes” (50–51%). Qualitative narratives confirmed these results. Persons with disabilities and returnee migrants frequently reported being labeled as “burdens” by families and communities, excluded from decision-making, or called derogatory names. A participant in one FGD noted, “People think we are weak because we returned from abroad without success.”

The formation and mobilization of SHGs and peer support networks were consistently described by participants as useful sources of psychosocial and social support. Participants reported opportunities to share concerns, reduce isolation, and seek advice from peers. These accounts suggest perceived benefits of peer-based support, although the qualitative data cannot establish the magnitude or causal effect of these activities. Some testimonies described situations in which individuals experiencing suicidal thoughts sought support from peers or group members. Participants perceived these interactions as helpful during periods of emotional difficulty; however, these accounts do not establish that the intervention directly prevented suicide attempts. Evidence from Nepal and beyond supports the role of peer and community groups in reducing isolation, enhancing resilience, and promoting recovery among persons with psychosocial distress [16].

The project also aimed to improve livelihoods and income for migrant families and PWDs. Quantitative findings show that 188 individuals engaged in self-employment or small businesses, and household income increased from NPR 20,552 to NPR 65,196 on average. This is supported by qualitative data, which showed that participants felt more positive and financially secure as a result of small business prospects. Some participants did, however, note that income sources are still uncertain, particularly for remittance-dependent households or those living in areas with little access to markets. Although the study assessed household income, it did not disaggregate between returnee migrants and non-migrant households. Since the project primarily targets migrants and their families, this lack of disaggregation limits the ability to fully capture the economic reintegration status of returnee migrants.

In terms of inclusion, the project ensured participation of marginalized groups including Dalits, Janajatis, women, and persons with disabilities within SHGs, awareness programs, and service delivery. Importantly, endline data also shows that PWDs were increasingly represented in mainstream community structures such as School Management Committees and Forest User Groups, reflecting progress toward social integration beyond project-supported platforms. This aligns with global recommendations for inclusive MHPSS programming that deliberately targets disadvantaged population [13]. However, challenges remain in disability certification processes, particularly for mental health-related disabilities, which limit access to government entitlements [14]. Strengthening advocacy and policy engagement is therefore critical for sustaining the gains in disability inclusion.

The evaluation reveals persistent challenges despite notable progress. A significant share of respondents continued to experience psychological distress and dissatisfaction with life, highlighting the complex and multi-layered determinants of mental health. Factors such as migration-related stress, family separation, and economic precarity extend beyond the scope of community-based interventions and require broader, multi-sectoral collaboration involving health, labor, education, and social protection systems [16]. At the same time, systemic barriers remain: access to psychotropic medicines is inconsistent, disability certification processes particularly for mental health-related conditions are bureaucratically complex and hinder access to entitlements [14], and subtle forms of stigma and discrimination persist within some households. These challenges underscore that while the PARBARDHAN project has strengthened community-level support and service delivery, addressing the deeper structural drivers of distress will require stronger health system integration, simplified administrative procedures, and sustained policy advocacy to ensure long-term, inclusive mental health care [23].

### 4.1 Strengths and limitations

This evaluation has several strengths, including the use of a mixed-methods design combining quantitative screening measures with qualitative accounts from multiple stakeholder and beneficiary groups. The use of standardized instruments and data collection across multiple project municipalities provided a broad assessment of psychosocial wellbeing and project experiences.

Several limitations should be considered when interpreting the findings. First, the endline quantitative sample was overwhelmingly female (94.3%) and predominantly comprised participants from migration-affected households. Although 88.2% reported having a family member currently working abroad, the available data did not provide a sufficiently clear mutually exclusive classification of all participants as migrant workers, left-behind family members, or other beneficiary groups. The findings therefore should not be generalized to all migrant workers in Nepal, particularly male migrant workers and returnees.

Second, baseline, midline and endline assessments comprised independent cross-sectional samples from overlapping but non-identical populations. Consequently, differences between evaluation phases may reflect differences in sample composition and contextual circumstances as well as differences in the underlying outcomes. The study cannot determine individual-level longitudinal change and does not provide a basis for attributing observed differences to the PARBARDHAN intervention.

Third, quantitative analysis was descriptive and no formal inferential testing was performed. The study was therefore not designed to estimate statistical differences or causal intervention effects between assessment phases.

Fourth, some outcomes were not available at all assessment points. In particular, life satisfaction and happiness were not assessed at baseline, and the midline sample available for these outcomes was smaller than the endline sample. Comparisons for these measures are therefore limited to the assessment points with available data and should be interpreted cautiously.

Fifth, GHQ-12 and HSCL-25 are screening instruments rather than diagnostic assessments. Their cut-off-based classifications should therefore not be interpreted as clinical diagnoses.

Finally, qualitative findings reflect participant and stakeholder experiences and perceptions. Although these findings provide important contextual information and help interpret quantitative patterns, they cannot independently establish program effectiveness or causal effects.

## 5. Conclusions

The endline evaluation identified heterogeneous patterns of psychosocial wellbeing among participants in the PARBARDHAN project areas of Salyan and Kailali. The proportions screening positive for anxiety and depression were lower at endline than at earlier assessment points, while the proportion screening positive for probable psychological distress was higher. Life satisfaction also showed a substantially different distribution between midline and endline, although these findings were based on independent samples and cannot be interpreted as individual-level change.Thus, the findings were not uniformly favorable across psychosocial outcomes.

Qualitative findings indicated that participants valued mental health awareness activities, self-help groups, peer support, counselling, and improved access to services. Participants also described continuing challenges related to economic insecurity, employment uncertainty, family separation, and migration-related stress. These findings suggest that community-based MHPSS support may be relevant to the needs of migration-affected communities, while also highlighting the persistence of broader structural determinants of psychosocial wellbeing.

Because the evaluation used independent cross-sectional samples and descriptive quantitative analysis, the observed differences cannot be interpreted as causal effects of the PARBARDHAN intervention. The findings primarily describe project beneficiaries and migration-affected households in the participating municipalities and should not be generalized to all migrant workers or their families in Nepal. Future evaluations should use longitudinal designs with consistent sampling and measurement across assessment points to assess individual-level change and strengthen causal inference.
