# Supplementary material for: Mental health and psychosocial wellbeing among beneficiaries of a community-based intervention in Nepal: Findings from a cross-sectional endline evaluation of the PARBARDHAN project
Source: PLOS Glob Public Health. 2026 Oct 16;6(10):e0006666. doi: 10.1371/journal.pgph.0006666 (PMC13674775; doi:10.1371/journal.pgph.0006666)
Supplement: Supplementary file 1 [file pgph.0006666.s002.pdf]

PLOS Global Public Health

Mental Health and Psychosocial Wellbeing of Migrant Workers and their left behind families in Nepal: Insights from the PARBARDHAN Endline Evaluation

--Manuscript Draft--

**Manuscript Number:**	PGPH-D-26-01439
**Article Type:**	Research Article
**Full Title:**	Mental Health and Psychosocial Wellbeing of Migrant Workers and their left behind families in Nepal: Insights from the PARBARDHAN Endline Evaluation
**Short Title:**	Mental Health of Migrant Workers in Nepal
**Corresponding Author:**	Himal Gaire, Mphil<br>CMC-Nepal: Center for Mental Health and Counseling- Nepal<br>Birendranagar, NEPAL
**Order of Authors:**	Himal Gaire, Mphil
	Ganesh Bhandari, MPH
	Rima Mishra, MPH
	Ram lal shrestha
	Aishwarya Prashad Dhakal
	Pashupati Mahat, Phd
**Keywords:**	mental health; psychosocial wellbeing; migrant workers; Nepal
**Abstract:**	Poor mental health among migrant workers has emerged as a serious public health concern affecting both workers and their families. This study aimed to examine the psychosocial wellbeing of migrant workers and their families by assessing psychological distress, coping strategies, social support, and stigma.<br>A cross-sectional, mixed-methods study with retrospective elements was conducted in Salyan and Kailali districts of Nepal to evaluate the PARBARDHAN project. The quantitative component surveyed 245 participants from 3–26 February 2026 which includes migrant workers, their family members, persons with psychosocial disabilities, members of self-help groups (SHGs), local government officials, health workers, and adolescents in the project communities using simple random sampling using validated tools to assess psychological distress, anxiety, and depression. The qualitative component involved interviews with Municipality executives, Health workers, Self-help groups, Peer support groups to explore project relevance and impact. Data analysis was performed using SPSS v26 for quantitative data and NVivo 12 for qualitative data, with ethical approval obtained from the National Health Research Council (NHRC).<br>Among 245 participants, 34.7% showed probable psychological distress, 8.2% had anxiety, and 5.3% exhibited depressive symptoms highlighting psychosocial challenges among migrant workers and their families. These outcomes reflect changes observed following the implementation of the PARBARDHAN project interventions conducted between the baseline and endline periods. During the period, the project addressed these challenges by increasing mental health awareness, reducing stigma, and strengthening coping strategies. Peer support groups and community activities further enhanced social and emotional support, improved mental health outcomes, and reduced suicide risk, with inclusive participation of marginalized groups ensuring equitable benefits.<br>The PARBARDHAN project effectively improved the psychosocial wellbeing of migrant workers and their families by increasing mental health awareness, reducing stigma, and strengthening social support through self-help groups. This highlights the need for continued investment in integrated mental health and psychosocial support (MHPSS) interventions.
**Additional Information:**	
**Question**	**Response**
**Financial Disclosure**	This work was conducted as part of an endline evaluation of a project implemented by

Commented [DM1]: Grammar error: This verb include should be in past tense and become (included)

Commented [DM2]: Typo error: , …person (you should write “and person with psychosocial disabilities”)

Commented [DM3]: Punctuation: Add comma after communities.

Enter a financial disclosure statement that describes the sources of funding for the work included in this submission and the role the funder(s) played. This includes grants and any commercial funding of the work or authors.<br><br>This statement will be typeset if the manuscript is accepted for publication.<br><br>*Please review the submission guidelines and the instructions link below for detailed requirements and guidance.*	CMC-Nepal with grant support from Felm. The funder (Felm) had no role in study design, data collection and analysis, decision to publish, or preparation of the manuscript.
**Competing Interests**<br><br>On behalf of all authors, disclose any competing interests that could be perceived to bias this work.<br><br>This statement will be typeset if the manuscript is accepted for publication.<br><br>*Please review the instructions link below and PLOS Global Public Health's competing interests policy to determine what information must be disclosed at submission.*	All other authors declare no competing interests.
**Data Availability**<br><br>Before publication, Authors are required to make fully available and without restriction all data underlying their findings. Please see our PLOS Data Policy page for detailed information on this policy.<br><br>A **Data Availability Statement**, detailing where the data can be accessed, is required at first submission. Insert your Data Availability Statement in the box	Due to ethical restrictions related to protecting participant confidentiality in this endline evaluation study, the underlying data cannot be made publicly available. Data are available upon reasonable request.

below.<br><br>Please see the data reporting section of our submission guidelines for instructions on what you need to include in your Data Availability Statement.<br><br>This statement will be typeset if the manuscript is accepted for publication.<br><br>*PLOS allows rare exemptions to address legal and ethical concerns. If you have legal or ethical restrictions, please use the box below to detail these in full sentences for the Journal team to consider.*	

Powered by Editorial Manager® and ProduXion Manager® from Aries Systems Corporation

Manuscript Click here to access/download;Manuscript;Manuscript.docx

Mental Health and Psychosocial Wellbeing of Migrant Workers and their left behind families in Nepal: Insights from the PARBARDHAN Endline Evaluation

Short title: Mental Health of Migrant Workers in Nepal

Himal Gaire[1*], Ganesh Bhandari[2], Rima Mishra[2], Ram Lal Shrestha[1], Aishwarya Prasad Dhakal[3], Pashupati Mahat[1]

Affiliations

[1]Centre for Mental Health and Counselling Nepal, CMC-Nepal

[2]Pokhara University, Nepal

[3]Mid-Western university

*Corresponding author

Himal.gaire@cmcnepal.org

Abstract

Poor mental health among migrant workers has emerged as a serious public health concern affecting both workers and their families. This study aimed to examine the psychosocial wellbeing of migrant workers and their families by assessing psychological distress, coping strategies, social support, and stigma.

A cross-sectional, mixed-methods study with retrospective elements was conducted in Salyan and Kailali districts of Nepal to evaluate the PARBARDHAN project. The quantitative component surveyed 245 participants from 3–26 February 2026 which includes migrant workers, their family members, persons with psychosocial disabilities, members of self-help groups (SHGs), local government officials, health workers, and adolescents in the project communities using simple random sampling using validated tools to assess psychological distress, anxiety, and depression. The qualitative component involved interviews with Municipality executives, Health workers, Self-help groups, Peer support groups to explore project relevance and impact. Data analysis was performed using SPSS v26 for quantitative data and NVivo 12 for qualitative data, with ethical approval obtained from the National Health Research Council (NHRC).

Among 245 participants, 34.7% showed probable psychological distress, 8.2% had anxiety, and 5.3% exhibited depressive symptoms highlighting psychosocial challenges among migrant workers and their families. These outcomes reflect changes observed following the implementation of the PARBARDHAN project interventions conducted between the baseline and endline periods. During the period, the project addressed these challenges by increasing mental health awareness, reducing stigma, and strengthening coping strategies. Peer support groups and community activities further enhanced social and emotional support, improved mental health

Commented [DM4]: Punctuation: you can add comma after 2026

Commented [DM5]: Punctuation: add comma after symptoms.

Commented [DM6]: Punctuation: Add comma after workers.

outcomes, and reduced suicide risk, with inclusive participation of marginalized groups ensuring equitable benefits.

The PARBARDHAN project effectively improved the psychosocial wellbeing of migrant workers and their families by increasing mental health awareness, reducing stigma, and strengthening social support through self-help groups. This highlights the need for continued investment in integrated mental health and psychosocial support (MHPSS) interventions.

Keywords: Mental health; psychosocial wellbeing; migrant workers; Nepal

Introduction

Nepal has an economy increasingly reliant on remittances generated by migrant workers. [1] Around 164 million people worldwide are migrant workers, marking a 9% increase between 2013 and 2017. Within this global context, Nepal plays significant role, with more than 300,000 Nepalis migrating abroad for employment each year. This makes Nepal one of the largest labor-sending nations in South Asia, supplying workers to countries across the region and beyond .[2]

While migration contributes substantially to Nepal's economy, poor mental health and illness among the migrant workers have emerged as serious socioeconomic and public health consequences [3], affecting not only the workers themselves but also their families and society/country. With a dramatic increase in work migration over the recent decades, there is recent concern about the health and wellbeing of migrant workers. [2] Some migrants face problems throughout the migration cycle leading to severe trauma and hardships that in turn have an adverse impact on their families left behind. On the other hand, problems encountered by family members left behind also affect migrant workers resident abroad .[4, 5] Post migration experiences such as exploitation, lack of legal protection, broken social networks, poor health care and discrimination in the destination country can lead to mental illness. [5] Previous studies have consistently shown that migrant workers experience higher rates of mental health problems compared to native workers, highlighting the urgent need for attention to their mental health and psychosocial wellbeing. [6]

The baseline study Enhancing Mental Health and Psychosocial Wellbeing of Migrant Workers and their Families (EMHPW-MW) project conducted by CMC-Nepal provided a comprehensive understanding of community knowledge, attitudes, practices, resources, and gaps related to mental

Commented [DM7]: Source of information: this is a global perspective, which should have the reference. For instance this link below is showing some source of global migrant workers. https://www.ilo.org/resource/news/new-ilo-figures-show-164-million-people-are-migrant-workers. However, the source of information on this sentence is needed to be added.

Formatted: Font color: Red

Commented [DM8]: Grammar: instead of there is recent concern about the health and wellbeing of migrant workers. It should be there ***has been a*** recent concern about the health and wellbeing of migrant workers.

Commented [DM9]: Punctuation: add comma between **cycle, and leading**

Commented [DM10]: Punctuation: add comma after **care,**

Commented [DM11]: Punctuation: add comma where it is necessary.

health, psychosocial issues, and migration through Key Informant Interviews (KII) and Focus Group Discussions (FGD). It also established baseline prevalence of stress, anxiety, and depression and updated key indicators of the project, offering an overall picture of mental health and psychosocial wellbeing in the project area, including access barriers, help-seeking behavior, community support mechanisms, cultural practices, and migration-related psychosocial risk factors.

Similarly, the midline evaluation of the Enhancing Mental Health and Psychosocial Wellbeing of Migrant Workers and their Families (PARBARDHAN) project assessed progress and impact of interventions on mental health and psychosocial wellbeing. Using a mixed-methods approach with surveys, FGDs, and KIIs, it found significant improvements in anxiety and depression levels, as well as increased happiness and life satisfaction. The evaluation also highlighted expansion of mental health services in greater health facilities, while identifying persistent challenges such as stigma, limited resources, and policy implementation ~~gaps.~~gaps. [7, 8]

Building on this foundation, the endline evaluation of the Enhancing Mental Health and Psychosocial Wellbeing for Migrant Workers and their Families (PARBARDHAN) project focused on changes in psychosocial wellbeing among migrant workers and their families. While the baseline and midline study identified gaps, needs, and contextual factors, the endline emphasized outcomes by assessing psychological distress, coping strategies, social support, and mental health-related stigma.The endline evaluation provided deeper insight into the manifestation of migration-related stressors and the effectiveness of coping mechanisms and support systems in building resilience. Additionally, issues of stigma and help-seeking, identified at baseline and midline, were examined more explicitly at endline, enabling assessment of persistent challenges

Commented [DM12]: Grammar: instead of It also established baseline prevalence of stress, you may write It also established **the** baseline prevalence of stress,

Commented [DM13]: Grammar: instead of assessed progress and impact, change to assessed **the** progress and impact

Commented [DM14]: highlighted **the** expansion

and emerging improvements. Overall, the endline evaluation allowed comparison with baseline and midline indicators and served as a key measure of progress and remaining gaps to guide the urgent need for targeted interventions that prioritize the mental health and wellbeing of both migrant workers and their families.

2. Materials and Methods

2.1 Study design and method

A cross-sectional study with retrospective elements was conducted to assess the relevance, effectiveness, efficiency, sustainability, and inclusiveness of the PARBARDHAN project, comparing findings with baseline data where available. A multi-method approach was used: quantitative surveys assessed mental health status and associated factors, while qualitative methods explored project relevance, effectiveness, and impact.

2.2 Study population and setting

Participants included migrant workers (returnees and families), persons with psychosocial disabilities, SHG members, adolescents, local health workers, and community or municipal representatives. The study was conducted in Salyan (Sharada, Bangad Kupinde, Bagchaur, Chattreshowri) and Kailali (Tikapur, Ghodaghodi, Janaki) districts.

2.3 Sample size and Sampling

For the quantitative component, a sample size of 212 was calculated using the Cochrane formula based on a 13.2% depression prevalence, with 245 participants ultimately surveyed via simple random sampling.

Commented [DM15]: Show how the sample size was calculated using Cochran's formula, and how a **non-response rate** was added.

For qualitative data, 7 FGDs (SHG and peer support groups) and 14 KIIs (local officials, health coordinators, facility staff) were conducted, guided by data saturation.

2.4. Data collection tools

2.4.1. Quantitative data collection

- General Health Questionnaire (GHQ-12): A 12-item tool to assess psychological distress. A score of 2 or above indicated psychological distress.[9]
- Hopkins Symptom Checklist (HSCL-25): A 25-item tool to measure anxiety and depression symptoms. A cutoff score of 1.78 or more indicated significant distress [10]
- Life Satisfaction Tool: A 6-item tool to assess satisfaction with various life domains using a 6-point Likert scale.
- Happiness Tool: A 25-item tool to measure subjective well-being using a 5- point Likert scale.
- Structured household questionnaire: Collected participants demographics, exposure to migration, household composition, disability, MHPSS access.

2.4.2 Qualitative Data collection

In depth Interviews (IDIs) were conducted with local government officials (mayors, deputy mayors, health section heads). An interview guide was used for IDI. Focus Group Discussions

(FGDs) were conducted with diverse community groups, including Self-Help Groups (SHGs), peer support groups. The FGD guideline was used for data collection.

2.5 Data collection process

Data for the PARBARDHAN endline evaluation were collected over 15 days in Kailali and Salyan by four trained Research Assistants (two per district). Prior to fieldwork, they received structured training from CMC-Nepal on study objectives, ethics, digital tools (KoBoToolbox), standardized instruments (GHQ-12, HSCL-25, Happiness, Life Satisfaction), qualitative methods, and referral procedures. Data collection involved coordination with local stakeholders to ensure proper recruitment and reduce non-response. Fieldwork was closely supervised by the lead evaluator and CMC-Nepal staff through visits, real-time data checks, and daily debriefings to maintain quality and address challenges.

2.6. Data Analysis

Quantitative analysis was conducted in SPSS v26, using descriptive statistics to summarize participant characteristics and outcomes (e.g., depression, anxiety, psychological distress, happiness, life satisfaction). Bivariate analyses examined associations with demographic variables, and results were compared to baseline to assess project impact.

Commented [DM16]: Grammar: instead of and result, you may say and **the** result.

Qualitative data were analyzed in NVivo 12 using thematic analysis, combining deductive (pre-defined themes like relevance, effectiveness, sustainability, disability inclusion) and inductive coding to identify major themes and subthemes.

2.7. Ethical considerations

The evaluation adhered to strict ethical guidelines to ensure the rights and well-being of participants. Ethical approval was obtained from the National Health Research Council (NHRC) (ref 1088), ensuring compliance with national research standards. All participants were informed about the study objectives, and written consent was obtained, when necessary, from legal guardians or caregivers. Confidentiality and participant autonomy were ensured through anonymized coding, secure encrypted storage, and assurance that personal information would be used solely for research purposes.

Results

3.1 Quantitative results

3.1.1 Socio-demographic characteristics of the respondents

Among 245 respondents, the mean age was 38.16 years (SD = 12.12), mostly aged 31–44 years (38.4%). The sample was predominantly female (94.3%), with over half from Salyan (57.1%). Ethnically, Chettri/Thakuri (40.4%) and Janajati (31.4%) were most common; 93.5% were Hindu. Educationally, 37.1% were literate and only 1.6% had a bachelor's or higher degree. Most were married (95.1%), with an average family size of 5.69 members. Nearly half lived in joint families (48.2%). The main occupations were housewives (38.4%) and agriculture (36.7%), with an average monthly family income of NPR 65,196 Table 1.

Commented [DM17]: You may slightly improve the writing for clarity:
"Among the 245 respondents, the mean age was 38.16 years **(SD ±12.12)**, with most participants aged 31–44 years (38.4%)."

Table 1. Background, socio-demographic and disability related characteristics of the participants.

Variable	Category	Frequency	Percentage
**Age**	Below 18	1	0.4
	18-30	74	30.2
	31-44	94	38.4
	≥45	76	31
	Mean: 38.16 and Sd: 12.12 Range: 16-80		
**Gender**	Male	14	5.7
	Female	231	94.3
**District**	Kailai	105	42.9
	Salyan	140	57.1

Commented [DM18]: The standard deviation should be within positive and negative range. **(SD ±12.12)**

Municipality	35 samples from all selected municipalities		
**Ethnicity**	Bhraman	21	8.6
	Chettri/Thakuri	99	40.4
	Janajati	77	31.4
	Dalit	48	19.6
**Religion**	Hindu	229	93.47
	Christian	13	5.31
	Others (Attamabiswasi, no belief, kuldevata)	3	1.22
**Education Level**	Literate (able to read and write)	91	37.14
	Primary Level	69	28.16
	Secondar level	48	19.59
	intermediate	33	13.47
	Bachelor level and above	4	1.63
**Marital status**	Married	233	95.1
	Unmarried	7	2.86
	Single or divorced	5	2.04
**Type of Marriage**	Arrange	103	42.04
	Love marriage	56	22.86
	Elopement	56	22.86
**Number of family members**	≤5	138	43.7
	>5	107	56.3
Mean	**5.69**		
**Type of family**	Nuclear	109	44.49
	Joint	118	48.16

	Extended	18	7.35
**Occupation**	Housewife	94	38.37
	Agriculture	90	36.73
	Business/self-employed	30	12.24
	Employed	21	8.57
	Daily allowance based	6	2.45
	Unemployed	4	1.63
**Family income**	Mean: 65196		
**Relationship with family member**	Good	187	76.33

3.1.2 Relationship status and remittance-related information

Table 2 presents information on family relationships, migration, and remittances. Most respondents reported good family relationships (76.3%), with smaller proportions describing them as normal (19.6%) or not good (3.2%). Among those with family abroad, husbands (51.4%) and children (29.7%) were the most common. Most households received remittances regularly (62.4%) or sometimes (20.8%), while few did not (4.9%) due to unemployment, loss of contact, or other reasons.

Commented [DM19]: a few

Table 2. Relationship status with family and remittance-related information.

Variable	Category	Frequency	Percentage
**Relationship with family member**	Good	187	76.33
	Normal	48	19.59
	Not good	8	3.27
	Very bad	2	0.82

Commented [DM20]: Grammar: Relationship with **a** family member

**Relation with family member who is in foreign**	Husband	126	51.43
	Son/daughter	73	29.70
	Father/mother	2	0.82
	No one	29	11.84
	Others	15	6.12
**Send Remittance**	Yes	153	62.45
	Sometime	51	20.82
	No	12	4.9
**Reason for not sending money (n=12)**	Unemployed	4	1.63
	Contact less	3	1.22
	Others	5	2.04

3.1.3 Mental health and Disability related characteristics of the respondents

According to Table 3, 27.8% reported mental health problems, mostly with onset over one year ago. First contact for care was primarily health posts (13.1%) or hospitals (4.9%), with few consulting traditional healers (0.4%). About 30.2% of them reported disabilities with majority having mental disability (70.3). All participants (100%) reported no vision or hearing problems after using glasses and hearing device. Most could walk easily (97.3%), 70.4% had some concentration difficulty and 65.8% some self-care difficulty; severe difficulties were reported by 1.4% and 5.5%, respectively. Communication was the most affected, with 32.4% reporting slight difficulty, 35.1% severe difficulty, and only 28.3% experiencing no problems. Half felt respected (51.4%) sometimes, 36.5% sometimes, and 12.2% not at all.

Commented [DM21]: with **the** majority

Table 3. Mental health and Disability related characteristics of the respondents.

Variable	Category	Frequency	Percentage
**Self-reported mental health status**	Yes	68	27.76
	No	177	72.24
**Time period when mental health problem was started (n=68)**	≤ one year	12	17.6
	One to five years	27	39.7
	>5year	29	42.6
**First point of contact point for mental health services (n=68)**	Basic Health service centers/health post	32	13.06
	District hospital	12	4.9
	Private hospital	9	3.67
	Traditional healers	1	0.41
	Others	14	5.71
**Disability**	Yes	74	30.2
	No	171	69.8
**Type of disability**	Physical	5	6.8
	Mental	52	70.3
	Vision related	1	1.4
	Intellectual	16	21.6
**Vision problem after using glass (n=74)**	No problem at all	74	100
**Hearing difficulty after using device (n=74)**			
**Difficulty in walking (n=74)**	No problem at all	72	97.30
	There is a little difficulty	2	2.70

**Difficulty in concentration (n=74)**	No problem at all	23	28.2
	There is a little difficulty	50	70.4
	There is a lot of difficulty.	1	1.4
**Self -care (n=74)**	No problem at all	22	28.8
	There is a little difficulty	48	65.8
	There is a lot of difficulty.	4	5.5
**Difficulty in Communication (n=74)**	No problem at all	23	100
	There is a little difficulty	24	32.4
	There is a lot of difficulty.	26	35.1
	Cannot do at all	1	1.4
**Felling respect from other**	Don’t feel respected at all	9	12.2
	Sometimes	38	51.4
	Most of the time	27	36.5

3.1.4 Psychological distress, anxiety and depression among the respondents (n=245)

Commented [DM22]: Punctuation: put a comma after anxiety

In Table 4, among the 245 participants, 160 (65.3%) scored 0–2 on the GHQ at endline evaluation, indicating no psychological distress, while 85 (34.7%) scored 3–12, suggesting probable distress. This represents an increase compared to the baseline prevalence of psychological distress (20.4%). At endline, most respondents were negative for anxiety (91.8%, n = 225), with 8.2% (n = 20) showing noticeable symptoms, reflecting a substantial reduction from the baseline prevalence of 37.2%. Similarly, depression was observed in 5.3% (n = 13) of participants at endline, compared to 26.2% at baseline, indicating marked improvement.

Table 4. Prevalence of psychological distress, anxiety and depression.

Prevalence of psychological distress (GHQ score)	n	%
Normal or no distress (GHQ=0-2)	160	65.3
Probable distress (3-12)	85	34.7
**Prevalence of anxiety (HSCL-25, Cut-off ≥ 1.75)**		
Anxiety: Negative (<1.75)	225	91.8
Anxiety: Positive (≥1.75)	20	8.2
**Prevalence of depression (HSCL-25, Cut-off ≥ 1.78)**		
Depression: Negative (<1.78)	232	94.7
Depression: Positive (≥1.78)	13	5.3

3.1.5 Life satisfaction and happiness level among respondents

Table 5 showed that among 245 participants, most reported low life satisfaction: 66.5% were dissatisfied, 15.9% slightly dissatisfied, and 6.1% extremely dissatisfied. Only a small proportion expressed satisfaction (2.0% satisfied, 5.7% slightly satisfied), while 3.7% were neutral. The Happiness Index showed that most were neutral (64.9%), about one-third were very happy (31%), and few were extremely happy (1.6%). Only a small minority reported being unhappy (2% slightly unhappy, 0.4% very unhappy).

Table 5. Life satisfaction and happiness level among respondents.

Life satisfaction	Endline	Midline
	Frequency (percent)	Frequency (percent)
Extremely dissatisfied	15 (6.1)	2(2.9)

Dissatisfied	163 (66.5)	3(4.4)
Slightly dissatisfied	39 (15.9)	6(8.8)
Neutral	9 (3.7)	4(5.9)
Slightly satisfied	14 (5.7)	23(33.8)
Satisfied	5 (2.04)	24(35.3)
Extremely Satisfied	0(0)	6(8.8)
**Happiness level**		
Very unhappy (4-8)	1 (0.4)	0(0)
Slightly unhappy (9-13)	5 (2.0)	3(3.3)
Neutral (14-18)	159 (64.9)	48(52.7)
Very happy (19-23)	76 (31.0)	24(26.4)
Extremely happy (24-28)	4 (1.6)	15(16.5)

3.2 Qualitative results

The project directly contributed to improving the psychosocial wellbeing of migrant workers and their families by reducing psychological distress, strengthening coping strategies, expanding social support, and challenging stigma.

3.2.1 Effectiveness of the project activities

Increased Awareness and Knowledge

One of the clearest outcomes of the project was the transformation in community knowledge and perceptions about mental health. Participants consistently reported a shift away from traditional and harmful beliefs (e.g., viewing mental illness as madness, a curse, or spirit possession) toward

understanding it as a health condition requiring treatment. This was facilitated through community awareness sessions, peer support group discussions, and public campaigns.

- *"Now, when we see such a person, we understand that they are experiencing mental health problems. There were many such people in our village."* (FGD, Kupinde)
- *"Before, people thought it was possession by spirits, now they understand it is illness that needs treatment."* (FGD, Kupinde)
- "Even family members started recognizing symptoms. They now come to us earlier, before the situation becomes worse." (KII, Health Worker, Janaki)
- "Previously, villagers didn't want to discuss mental health. After awareness initiatives, they started asking questions and seeking advice." (IDI, Health Coordinator)

3.2.2 Behavior Change and Reduced Stigma

Another strong outcome of the project was the reduction in stigma and discriminatory practices toward individuals with mental health conditions. Both community members and health workers observed a clear shift in how people with psychosocial disabilities were treated within households and society. Where previously families and communities practiced avoidance or exclusion, they are now increasingly inclusive and supportive.

- *"Now everyone eats together in the same place; they don't say those who are ill should be separated."* (FGD, Kupinde)
- *"Overall, this project has brought positive changes in people with mental health problems. We talk about counseling, basic income generation, and therapeutic services. Together, these three aspects help to reduce the problems*."(IDI, Tikapur)

- *“Significant changes have occurred. Beneficiaries themselves say that they were previously in a disturbed state physically or mentally. After receiving support from the project, they have become mentally stronger and are now able to interact easily with their communities and families. Earlier, there were situations where families themselves discriminated against them, but now that is no longer the case”. (IDI, Ghodaghodi)*

3.2.3 Emotional and Social Support

Beyond formal health services, peer groups and community-based activities emerged as vital sources of emotional and social support. Group members consistently described these safe spaces as avenues for sharing personal struggles, reducing loneliness, and providing encouragement in times of crisis. Such peer-led support mechanisms contributed not only to improved well-being but also to suicide prevention within communities.

- *“We go to the program, we listen to each other’s stories, and that makes us feel very good. Sharing your thoughts with a friend makes your heart feel lighter.”* (FGD, Kupinde)
- *“One of our sisters had suicidal thoughts, but after she read a pamphlet and called me, I counseled her and she decided to live.”* (FGD, Janaki)

3.2.4 Impact of the project

Improved Mental Health Outcomes

A key impact of the project was improved mental health outcomes among participants and their families. Respondents across IDIs and FGDs consistently highlighted noticeable changes in recovery, emotional regulation, and daily functioning. These improvements were attributed to increased access to counseling, medication, peer support, and awareness initiatives.

- *"There has been a change in cleanliness, and now we know that taking medicine helps to recover. We are now able to concentrate and work better."* (FGD, Kupinde)
- *"Significant changes have occurred. Beneficiaries themselves say that they were previously in a disturbed state physically or mentally. After receiving support from the project, they have become mentally stronger and are now able to interact easily with their communities and families."* (KII, Ghodaghodi)

Reduction in Suicide Risk

Several testimonies described lives saved from suicidal ideation. Both FGD and KII participants recalled cases where project interventions — such as peer counseling, group discussions, and timely referrals to health facilities — directly prevented suicide attempts.

- *"One woman had suicidal thoughts, but after counseling and support, her health improved greatly."* (FGD, Kupinde)
- *"We even advised patients who were having suicidal thoughts. After we talked with them, they said their mind felt lighter."* (FGD, Janaki Municipality SHG)
- *"I counseled a sister who had suicidal feelings after her husband migrated abroad. She said later that our talk gave her the courage to live."* (FGD, Peer Support Group Member, Ghodaghodi)
- *"While thinking and thinking, many thoughts come—even thoughts of suicide. That is why it is necessary to participate in such groups."* (FGD, Kupinde)

Smooth operation of Self-help Group

Both local stakeholders and SHG members expressed confidence in their ability to sustain and operate the groups independently in the future. The groups have developed leadership capacity,

internal trust, and established routines that make their continuity feasible even without intensive external support.

- *"Absolutely, they can operate independently. We have trained them in various stages on how to operate independently. We can continue to give them orientation and workshops to make them self-reliant. They may not need any external supporting organization in the future. They have also committed to operating independently after the project ends."(IDI Health Coordinator)*
- *"We sit together, share our problems, and manage on our own. Even if the project ends, we can continue meeting."* (FGD, Janaki Municipality SHG)
- *"We feel this program should have continued for some more years so we could learn more, but still, we are confident we can run the group."* (FGD, Kupinde)
- *"The municipality and health posts know about our groups. If there is any problem, we can coordinate with them."* (IDI, Municipal Representative)

3.2.5 Inclusion & Equity

Participation of Marginalized Groups

Dalits, women, persons with disabilities, and Janajatis were actively included. Both IDI and FGD respondents emphasized that equal participation was encouraged, and deliberate efforts were made to invite those who were often left behind.

- *"Those who don't have information, we call them and invite them. Dalits, women, and Janajatis all have equally received their rights."* (FGD, Kupinde)

- *"In our group, there are Dalit members and also people with disabilities. Everyone is given the same chance to participate."* (FGD, Janaki Municipality SHG)
- *"My son has a disability, but through this group we were supported and never excluded."* (FGD, Janaki Municipality SHG)
- *"Services are being provided equally to everyone."* (FGD, Kupinde)

Marginalized communities, who often face layered stigma and exclusion, were deliberately targeted for inclusion in awareness programs, counseling, and self-help groups.

Disability Inclusion

Respondents shared that **access to disability allowances and services had begun**, and awareness had reduced negative labeling. However, challenges persisted in obtaining formal certification for psychosocial disabilities, which limited access to certain government benefits.

- *"The identity card for psychosocial disability has not come yet, but treatment is happening."* (FGD, Kupinde)
- *"Compared to before, people with mental illness used to be called mad. Now everyone understands they need hospital treatment."* (FGD, Kupinde)
- *"For physical disabilities, the allowance is provided. But for mental health problems, the government process is still complicated."* (KII, Health Facility Incharge, Janaki)

Discussion

The findings highlight measurable improvements in mental health outcomes and service utilization. Quantitative findings reveal reductions in anxiety and depression compared to baseline, along with improvements in life satisfaction and happiness. Qualitative evidence complements these results, as beneficiaries consistently described improved coping, stronger family support, and reduced stigma in their communities. Engagement of municipalities in planning and resource allocation signals progress toward sustainability.

One of the most significant achievements observed in this evaluation is the reduction in anxiety and depression. The prevalence of depression and anxiety among participants decreased substantially over the project period, with respondents reporting improved emotional regulation, resilience, and overall wellbeing. Qualitative findings align with this trend. Respondents consistently reported improved mental health, attributing it to psychosocial counseling, awareness programs, and peer support groups. While these improvements cannot be solely attributed to project interventions, it is evident that the project's activities—such as counseling, awareness campaigns, and capacity-building of health workers—played a contributory role in creating enabling environments for early identification and treatment. Previous studies in Nepal and other low-resource settings have shown that community-based psychosocial interventions and mhGAP training significantly improve detection and management of common mental disorders, leading to reduced psychological distress.[11, 12]

Interestingly, psychological distress as measured by the GHQ-12 showed an increase from baseline and endline. This tool is sensitive to short-term stressors and captures broader psychosocial and physical strains, suggesting that while anxiety and depression were addressed,

ongoing challenges linked to migration, economic insecurity, and family separation continued to generate transient distress. [13] Qualitative data helps in explaining this contradiction where respondents highlighted some stressors such as economic hardship and uncertainty of employment. Also, it is possible that increased awareness and openness created by project enabled them to recognize and report their psychological distress. The quantitative results show a contrast between midline and endline findings regarding life satisfaction. At midline, the majority of respondents expressed positive life satisfaction, with nearly four in five reporting being slightly satisfied, satisfied, or extremely satisfied. By endline, this pattern reversed dramatically: more than 88% of respondents reported being dissatisfied to some extent, and only a very small proportion (about 8%) expressed any level of satisfaction. This indicates a substantial decline in life satisfaction among the target population over the project period. This change has some meaning according to qualitative research. Issues including work insecurity, unstable finances, and concern regarding future employment prospects were commonly brought up by respondents. Despite having access to psychosocial support during the experiment, migrant workers specifically underlined the stress of inconsistent income and the load of debt, which may have affected their feeling of life satisfaction.

The project was also highly relevant to community priorities. Migrant workers and their families often burdened by separation, uncertainty, and financial strain—described the interventions as both timely and essential. Health workers highlighted that the project enabled them to identify individuals who were previously hidden due to stigma and connect them with care, directly addressing a critical service gap. The cultural acceptability of the strategies, such as peer support groups, SHGs, and local awareness campaigns, further reinforced their relevance. These

interventions aligned with values of solidarity, collective responsibility, and mutual support, helping normalize conversations about mental health and reduce resistance to participation.

The project also contributed to increased availability and utilization of mental health services. At baseline, access to MHPSS services was very limited, with only 4 health facilities providing such support and from 4 to 19 health facilities till midline. By endline, this number had expanded to 29 health facilities, reflecting a significant scale-up in service availability alongside enhanced referral mechanisms. Trained personnel were able to deliver quality services at primary health facilities, which improved trust and service uptake within communities. Qualitative findings have supported that increased service availability and utilization has showed strong programmatic achievement. Participants expressed satisfaction with improved access, particularly mentioning availability of medicine at municipal level and trained counselors at the health facilities.

Qualitative findings indicate that stigma surrounding mental health has considerably reduced in the intervention areas. Respondents described a shift from harmful beliefs such as associating mental illness with spirit possession or weakness to greater recognition of mental health conditions as treatable. Families that previously excluded or discriminated against persons with psychosocial disabilities were increasingly supportive and inclusive. These changes reflect the effectiveness of targeted awareness campaigns, peer learning, and SHG activities in shifting social norms. Yet both findings reveal that stigma remains a significant barrier to mental health and well-being. Quantitatively, 11.8–16.7% of respondents reported feeling ‘not at all respected,’ while the majority indicated they were respected only “sometimes” (50–51%). Qualitative narratives confirmed these results. Persons with disabilities and returnee migrants frequently reported being labeled as “burdens” by families and communities, excluded from decision-making, or called

derogatory names. A participant in one FGD noted, “People think we are weak because we returned from abroad without success.”

The formation and mobilization of SHGs and peer support networks proved highly effective in providing both psychosocial and economic support. Group members reported improved coping, decreased loneliness, and greater confidence in managing mental health challenges. Testimonies highlighted instances where peer-led counseling and group discussions directly prevented suicide attempts, underscoring the importance of informal support networks in suicide prevention. Evidence from Nepal and beyond supports the role of peer and community groups in reducing isolation, enhancing resilience, and promoting recovery among persons with psychosocial distress. [14]

The project also aimed to improve livelihoods and income for migrant families and PWDs. Quantitative findings show that 188 individuals engaged in self-employment or small businesses, and household income increased from NPR 20,552 to NPR 65,196 on average. This is supported by qualitative data, which showed that participants felt more positive and financially secure as a result of small business prospects. Some participants did, however, note that income sources are still uncertain, particularly for remittance-dependent households or those living in areas with little access to markets. Although the study assessed household income, it did not disaggregate between returnee migrants and non-migrant households. Since the project primarily targets migrants and their families, this lack of disaggregation limits the ability to fully capture the economic reintegration status of returnee migrants.

In terms of inclusion, the project ensured participation of marginalized groups—including Dalits, Janajatis, women, and persons with disabilities—within SHGs, awareness programs, and service delivery. Importantly, endline data also shows that PWDs were increasingly represented in

mainstream community structures such as School Management Committees and Forest User Groups, reflecting progress toward social integration beyond project-supported platforms. This aligns with global recommendations for inclusive MHPSS programming that deliberately targets disadvantaged population. [15] However, challenges remain in disability certification processes, particularly for mental health-related disabilities, which limit access to government entitlements. Strengthening advocacy and policy engagement is therefore critical for sustaining the gains in disability inclusion. [16]

The evaluation reveals persistent challenges despite notable progress. A significant share of respondents continued to experience psychological distress and dissatisfaction with life, highlighting the complex and multi-layered determinants of mental health. Factors such as migration-related stress, family separation, and economic precarity extend beyond the scope of community-based interventions and require broader, multi-sectoral collaboration involving health, labor, education, and social protection systems. [14] At the same time, systemic barriers remain: access to psychotropic medicines is inconsistent, disability certification processes—particularly for mental health-related conditions—are bureaucratically complex and hinder access to entitlements, [17] and subtle forms of stigma and discrimination persist within some households. These challenges underscore that while the PARBARDHAN project has strengthened community-level support and service delivery, addressing the deeper structural drivers of distress will require stronger health system integration, simplified administrative procedures, and sustained policy advocacy to ensure long-term, inclusive mental health care.

Conclusions

The PARBARDHAN project effectively addressed the mental health and psychosocial wellbeing of migrant workers and their families by increasing mental health awareness and knowledge, fostering behavioral changes toward inclusivity, and improving access to emotional and social support through self-help groups and peer support mechanisms and reducing stigma surrounding mental health issues. The findings emphasize the urgent need for sustained investment in mental health and psychosocial support (MHPSS) for migrant workers and their families. Interventions that combine awareness, stigma reduction, peer-led support, livelihood opportunities, and government engagement are vital to safeguarding psychosocial wellbeing. Scaling up such approaches within local health systems and policy frameworks can contribute to more resilient families and communities in the face of migration-related challenges.

Financial Disclosure

This work was conducted as part of an endline evaluation of a project implemented by CMC-Nepal with grant support from Felm. The funder (Felm) had no role in study design, data collection and analysis, decision to publish, or preparation of the manuscript.

Competing Interests

All other authors declare no competing interests.

Data Availability

A The data that support the findings of this study are available from CMC-Nepal but restrictions apply to the availability of these data, which were used under ethical approval for the current study. Data are, however, available from the authors upon reasonable request and with permission of CMC-Nepal.

Acknowledgements

The authors thank all participants migrant workers, family members, persons with psychosocial disabilities, SHG and peer support group members, health workers, and local government officials for their valuable contributions. We acknowledge the support of municipal authorities in Salyan and Kailali districts, the National Health Research Council (NHRC) for ethical approval (ref 1088), and the research assistants for fieldwork. We are grateful to CMC-Nepal for technical support and coordination.

References

1. Mahat P, Thorley K, Kunwar K, Ghirime SJm. Mental health problems in Nepalese migrant workers and their families. 2020:2020.08. 04.20168104.

2. Devkota HR, Bhandari B, Adhikary PJJoM, Health. Perceived mental health, wellbeing and associated factors among Nepali male migrant and non-migrant workers: A qualitative study. 2021;3:100013.

3. Mucci N, Traversini V, Giorgi G, Tommasi E, De Sio S, Arcangeli GJS. Migrant workers and psychological health: A systematic review. 2019;12(1):120.

4. Hettige S, Ekanayake ES, Jayasundere R, Rathnayake A, Figurado PJC, Sri Lanka: Ministry of Foreign Employment Promotion, Welfare. Understanding psychosocial issues faced by migrant workers and their families. 2012.

5. Dhungana RR, Aryal N, Adhikary P, Kc RK, Regmi PR, Devkota B, et al. Psychological morbidity in Nepali cross-border migrants in India: a community based cross-sectional study. 2019;19(1):1534.

6. Hasan SI, Yee A, Rinaldi A, Azham AA, Mohd Hairi F, Amer Nordin ASJPo. Prevalence of common mental health issues among migrant workers: A systematic review and meta-analysis. 2021;16(12):e0260221.

7. Gaire H, Bhandari G, Verma M, Karki S, Nath L, Chaudhary A, et al. Provision of mental health and psychosocial support services at the community level in Nepal: Insights from a midterm evaluation. 2025;4(1):3068-.

8. Gaire H, Shrestha RL, Mhhat P, Chaudhary A, Bhandari GJEJoPH. 348; Mental health and psychosocial well-being of migrant workers and their families: policy and practice of Nepal. 2025;35(Supplement_6).

9. Maridal HK, Bjørgaas HM, Hagen K, Jonsbu E, Mahat P, Malakar S, et al. Psychological distress among caregivers of children with neurodevelopmental disorders in Nepal. 2021;18(5):2460.

10. Thapa SB, Hauff EJSp, epidemiology p. Psychological distress among displaced persons during an armed conflict in Nepal. 2005;40(8):672-9.

11. Kohrt BA, Jordans MJ, Turner EL, Rai S, Gurung D, Dhakal M, et al. Collaboration with people with lived experience of mental illness to reduce stigma and improve primary care services: a pilot cluster randomized clinical trial. 2021;4(11):e2131475-e.

12. Luitel NP, Jordans MJ, Adhikari A, Upadhaya N, Hanlon C, Lund C, et al. Mental health care in Nepal: current situation and challenges for development of a district mental health care plan. 2015;9(1):3.

13. Li R, Liu S, Huang C, Darabi D, Zhao M, Heinzel SJH. The influence of perceived stress and income on mental health in China and Germany. 2023;9(6).

14. National Mental Health Survey 2020. Kathmandu, Nepal: Nepal Health Research Council; 2021.

15. World Health Organization. WHO Guidance on Community Mental Health Services: Promoting Person-Centered and Rights-Based Approaches. 2021.

16. World Health Organization. Improving access to mental health services by integrating them into general health services in Nepal. . 2022.

17. Bachhav AK, Sharma HB, Singhai KMJIJoP. Challenges in disability certification in psychiatry in India. 2025;67(7):707-9.

499

Response to Editorial Office – Resubmission of PGPH-D-26-01321

Dear Editors,

Thank you for your request.

This manuscript is a resubmission of the accidentally withdrawn submission PGPH-D-26-01321. The previous submission was withdrawn by the author in error before it entered peer review. No editorial decision or reviewer comments were ever issued.

Therefore, no changes have been made to the manuscript itself as there were no reviewer or editor comments to address.

However, the following administrative corrections have been made to the submission record to comply with PLOS policies:

- Financial disclosure statement added
- Competing interests statement added
- Data availability statement added

The manuscript file remains unchanged from the original submission.

We respectfully submit this manuscript for your consideration and apologize again for the administrative error.

Sincerely,

Dr. Ganesh Bhandari
