# Supplementary material for: Mental health and psychosocial wellbeing among beneficiaries of a community-based intervention in Nepal: Findings from a cross-sectional endline evaluation of the PARBARDHAN project
Source: PLOS Glob Public Health. 2026 Oct 16;6(10):e0006666. doi: 10.1371/journal.pgph.0006666 (PMC13674775; doi:10.1371/journal.pgph.0006666)
Supplement: Supplementary file 2 [file pgph.0006666.s006.pdf]

MANUSCRIPT EVALUATION

General Comment

I thank the editor and the authors for the opportunity to review this manuscript. The study addresses a relevant public health problem related to the mental health and psychosocial well-being of migrant workers and their families in Nepal, within the context of the endline evaluation of the PARBARDHAN project. The topic is relevant to the scope of *PLOS Global Public Health*, and the use of quantitative and qualitative methods has the potential to generate useful information for the planning of community interventions.

However, after a detailed evaluation of the manuscript, I consider that the current version requires a major and substantial revision before it can be considered for publication. The main concerns do not correspond solely to aspects of editorial presentation, but rather to issues related to the coherence between the study design and the inferences made, the comparability of the different evaluation rounds, the definition of the study population, the statistical strategy, the interpretation of the results, the depth of the Introduction and Discussion, and the documentation of ethical aspects.

The central problem running throughout the manuscript is that different sections use expressions that suggest program effectiveness or impact, whereas the described design corresponds to a cross-sectional evaluation and the different evaluation rounds do not appear to correspond to longitudinal follow-up of the same individuals. Therefore, it is necessary to carefully revise the way the results are presented and discussed in order to avoid causal inferences that cannot be supported by the design.

Likewise, I consider that the Introduction and, particularly, the Discussion require substantial reconstruction in order to adequately integrate the available scientific evidence, including relevant systematic reviews and meta-analyses, and to discuss in a balanced manner both favorable findings and those showing discordant or unfavorable results.

1. TITLE

Persistent Problem

The manuscript title is:

"Mental Health and Psychosocial Wellbeing of Migrant Workers and their left behind families in Nepal: Insights from the PARBARDHAN Endline Evaluation."

Although the title identifies the general topic and the program evaluated, it does not fully represent the population included and does not provide sufficient information about the design.

The manuscript reports that the sample included different groups of participants, not exclusively migrant workers and their family members. These include, among others,

people with psychosocial disabilities, members of self-help groups, healthcare workers, officials, and adolescents. Therefore, the designation “Migrant Workers and their left behind families” may be too restrictive to describe the population actually analyzed.

Additionally, “Insights from” is a nonspecific formulation for an original research article and does not inform the reader about the nature of the evaluation.

Additional Problem Related to the Design

The title should be particularly careful regarding the use of terms that may suggest longitudinal follow-up of individuals. The manuscript subsequently acknowledges that the different evaluation rounds correspond to independent samples. Therefore, any title presenting the research as a longitudinal evaluation should be used only if the authors can demonstrate that a true longitudinal component exists at the individual level.

Requested Action

The authors should revise the title so that it accurately represents the population and study design. If baseline, midline, and endline correspond to independent cross-sectional samples, any terminology suggesting individual longitudinal follow-up should be avoided.

I also recommend replacing “Insights from” with a more informative formulation that clearly identifies the nature of the evaluation.

2. ABSTRACT

2.1. Use of Causal Language

The abstract states that the results:

“reflect changes observed following the implementation”

and concludes that:

“The PARBARDHAN project effectively improved the psychosocial wellbeing…”

These statements are too strong for the described design.

The manuscript identifies the study as cross-sectional with retrospective elements and subsequently acknowledges that the samples from the different rounds are not necessarily the same individuals.

A difference between two cross-sectional samples taken at different time points does not, by itself, demonstrate a change attributable to the intervention.

Requested Action

The authors should review the entire abstract and replace expressions implying causal effectiveness with language compatible with the design, for example:

- “differences observed between evaluation rounds”;
- “findings were consistent with potential benefits”;
- “participants reported perceived benefits”;

provided that these expressions correspond to the data actually available.

It should not be stated that the project “effectively improved” well-being without a design that allows such an inference.

2.2. Statement Regarding Suicide Prevention

The abstract presents the intervention as contributing to a reduction in suicide risk. However, the manuscript does not present a population-level assessment of suicide risk that would allow a reduction in this outcome to be estimated.

Qualitative testimonies from participants reporting that they received help during a crisis may demonstrate perceived experiences of support, but they do not allow estimation of a reduction in suicide risk in the population or establishment of causality.

Requested Action

The authors should remove or reformulate any statement indicating that the program reduced suicide risk or prevented suicide attempts at the population level, unless they can provide specific and systematic analyses supporting such a claim.

Qualitative results should be presented as experiences or perceptions reported by the participants.

3. INTRODUCTION

3.1. Insufficiently Developed Conceptual Framework

I consider that the Introduction requires substantial restructuring.

The Introduction appropriately establishes that labor migration may affect mental health and mentions factors such as family separation, discrimination, exploitation, and difficulties accessing services. However, the manuscript subsequently studies multiple domains:

- psychological distress;
- anxiety;
- depression;
- life satisfaction;
- happiness;

- stigma;
- social support;
- disability;
- access to services;
- self-help groups;
- sustainability and inclusion.

The Introduction does not sufficiently develop the conceptual framework connecting these domains with labor migration and with the intervention being evaluated.

Requested Action

The authors should expand and reorganize the Introduction to develop:

1. the magnitude of the mental health problem among migrant workers;
2. the situation of families remaining in the country of origin;
3. the main psychosocial determinants associated with labor migration;
4. the consequences of family separation and economic conditions;
5. barriers to accessing mental health services;
6. evidence on community interventions and MHPSS;
7. evidence specific to Nepal and South Asia;
8. comparable international evidence;
9. the remaining knowledge gap;
10. the specific scientific rationale for the endline evaluation.

3.2. Insufficient Use of Systematic Reviews and Meta-analyses

The manuscript includes some synthesis references, but these are not used sufficiently in depth to construct the scientific argument.

Considering the breadth of the question, the authors should review and incorporate, when relevant, systematic reviews and meta-analyses on:

- mental health of migrant workers;
- mental health of family members of migrant workers;
- mental health interventions in migrant populations;

- community-based MHPSS interventions;
- peer support;
- mental health-related stigma;
- interventions in low- and middle-income countries.

The purpose is not simply to increase the number of references, but to establish what is known, which results have been consistent, which interventions have demonstrated benefits, and what uncertainties remain.

Requested Action

The authors should conduct a substantial update of the literature and use relevant systematic reviews/meta-analyses to construct both the rationale for the study and the subsequent interpretation of the results.

General statements regarding prevalence, risk factors, intervention effectiveness, or mechanisms should be supported by appropriate evidence.

3.3. Insufficiently Defined Knowledge Gap

The Introduction describes the PARBARDHAN program and its previous evaluations, but does not sufficiently precisely define what new scientific question this study answers.

It is not sufficient to state that an endline evaluation was conducted.

Requested Action

The authors should explicitly formulate:

- what knowledge was missing after baseline and midline;
- what hypothesis or scientific question the endline addresses;
- what outcomes constitute the primary outcomes;
- what additional information this study provides;
- and how its results contribute to existing knowledge.

4. METHODS

4.1. Study Design and Attribution of Effectiveness

This is one of the main methodological concerns.

The manuscript describes the study as cross-sectional and uses the expression 'retrospective elements'; however, it is not sufficiently clear what information was obtained

retrospectively, what time period this information covers, and how this characteristic relates to the overall study design.

The problem is not evaluating a program through a cross-sectional study. This can be perfectly valid for certain objectives. The problem is drawing causal inferences that the design does not allow.

There is no evidence presented of:

- random allocation;
- control group;
- comparator group;
- individual follow-up;
- longitudinal analysis of participants;
- quasi-experimental design.

Requested Action

The authors should:

1. precisely describe the epidemiological design;
2. explain exactly what “retrospective elements” means;
3. clarify what information corresponds to each round;
4. indicate whether any participant was followed individually;
5. differentiate descriptive comparisons from causal evaluation;
6. remove from the Results, Discussion, and Conclusions any causal language that cannot be supported.

If the authors consider that the study allows effectiveness to be evaluated, they should provide an explicit methodological justification for this inference. I suggest that they explicitly define which variables or information were collected retrospectively, when they were obtained, and what their relationship was to the endline evaluation.

4.2. Comparability of Baseline, Midline, and Endline

This is probably the most important methodological problem in the manuscript.

The manuscript compares results from different phases but acknowledges that the samples are independent.

Therefore, an observed difference between baseline and endline may be due to:

- the intervention;
- differences in sample composition;
- sociodemographic differences;
- territorial differences;
- differences in migration status;
- economic changes;
- temporal changes;
- selection differences;
- differences in instruments or procedures.

This is particularly relevant because the endline sample presents an extremely imbalanced sex distribution: 231 women (94.3%) and only 14 men (5.7%).

Requested Action

The authors should present a detailed comparison of the baseline, midline, and endline samples, including at minimum:

- sample size;
- age;
- sex;
- district;
- migration status;
- migrant worker/family member status;
- socioeconomic variables;
- disability;
- other relevant characteristics.

In addition, they should explain whether the same sampling frame was used and whether the selection procedures were comparable.

Finally, the Discussion should acknowledge that differences between independent samples cannot be directly interpreted as individual changes over time.

4.3. Population Composition

There is an important discrepancy between the population suggested by the title and the population actually analyzed.

A considerable proportion of the sample does not clearly correspond to migrant workers.

Requested Action

The authors should clearly present the number and proportion of participants belonging to each population category and explain:

- why they were included;
- what analyses were conducted for each group;
- whether all participants were exposed to the intervention;
- and whether outcomes were analyzed jointly or by subgroups.

If conceptually different populations are combined, the authors should epidemiologically justify this decision.

4.4. Sample Size

The authors use a prevalence of depression to justify the sample size calculation, but the study evaluates numerous outcomes.

It is not sufficiently clear which was the primary outcome used to determine the sample size or whether the calculated sample size provides adequate precision for the other results.

Requested Action

The authors should specify:

- the outcome used for the calculation;
- assumed prevalence;
- confidence level;
- precision;
- design effect, if applicable;
- expected losses or missing data;
- justification of the sample size for the qualitative component.

They should also clarify whether the study was designed to estimate prevalences or to compare phases.

4.5. Instruments and Measurement Validity

GHQ-12, HSCL-25, and life satisfaction and happiness tools are used.

However, the methodological description is not sufficiently complete to assess the validity of the measurements in the population studied.

Requested Action

The authors should specify for each instrument:

- version used;
- language;
- translation/adaptation process;
- evidence of validation in the Nepalese population;
- cut-off point;
- rationale for the cut-off point;
- relevant psychometric properties;
- treatment of missing data;
- scoring method.

If the cut-off points were developed for another population, this should be explicitly acknowledged as a limitation.

4.6. Statistical Analysis

The statistical section requires greater specificity.

The manuscript does not allow the following to be fully reconstructed:

- which statistical tests were used;
- how the rounds were compared;
- which measures of association were calculated;
- how continuous variables were handled;
- how missing data were handled;
- whether adjusted analyses were performed;
- how covariates were selected;
- whether sensitivity analyses were performed.

Requested Action

The authors should fully expand the statistical plan and specify:

1. tests used for each comparison;
2. effect measures;
3. confidence intervals;
4. treatment of missing data;
5. criteria for covariate inclusion;
6. relevant stratified analyses;
7. sensitivity analyses;
8. handling of multiple comparisons when applicable.

When proportions are compared between phases, it is recommended that absolute differences and their confidence intervals be presented, in addition to p-values when appropriate.

4.7. Qualitative Component

The description of the qualitative component does not reach the level of detail necessary for reproducibility.

It is not sufficiently clear how the following were conducted:

- participant selection;
- number of interviews/groups;
- saturation;
- coding;
- development of categories;
- triangulation;
- participation of different researchers;
- resolution of discrepancies.

Requested Action

The authors should expand the qualitative methods and clearly specify the analytical procedure, including how themes were generated and how the credibility of the findings was ensured.

4.8. Integration of Mixed Methods

Presenting quantitative and qualitative results does not, by itself, demonstrate adequate integration of mixed methods.

Currently, the components appear largely separate.

Requested Action

The authors should explain:

- what the mixed-methods design was;
- which component had priority;
- how the results were integrated;
- how qualitative data helped explain the quantitative results;
- how discordant results were addressed.

An explicit integration of the quantitative and qualitative findings would be particularly useful, rather than presenting both components as independent narratives.

4.9. Ethical Approval

The manuscript indicates approval from the National Health Research Council (NHRC), reference 1088, but does not provide the approval date.

The reported collection period is **3–26 February 2026**. There is a profound inconsistency.

Requested Action

The authors should indicate:

the exact date on which ethical approval was granted by the National Health Research Council (NHRC; reference 1088) and confirm whether this approval was obtained before the start of participant recruitment and data collection (3–26 February 2026).

Likewise, I recommend that they provide the corresponding ethical approval document as supplementary material, if permitted by the journal's editorial policies.

What was the exact date of ethical approval from the NHRC (ref. 1088), and was it granted before the start of recruitment and data collection? The original manuscript had 3–26 February 2026, whereas the subsequent revision states that it was actually 3–26 February 2025. Therefore, the approval date becomes important from a documentary standpoint.

4.10. Consent and Minor Participants

The manuscript includes adolescents, but it is not sufficiently clear how consent/assent was managed for minor participants.

Requested Action

The authors should specify:

- age range of the adolescents included;
- who provided consent;
- whether assent was obtained from the minor;
- procedure used;
- specific ethical approval for the inclusion of minors.

5. RESULTS

5.1. Sample Characterization and Transparency of Denominators

Table 1 should provide a more complete and transparent characterization of the study population. The sample presents a markedly imbalanced sex distribution and a heterogeneous composition, aspects that limit the generalizability of the results to all Nepalese migrant workers and their families.

The authors should expand the characterization of the sample to clearly identify who constitutes the analyzed population and, when applicable, differentiate between migrant workers, family members remaining in Nepal, and other groups included in the evaluation. This information should be consistent with the population defined in Methods and with the population to which the results are subsequently generalized.

Requested Action:

The authors should revise and expand Table 1 to clearly present the composition of the sample, including relevant variables for identifying the different participant groups. When methodologically appropriate, stratified analyses could be considered; if these are not possible due to the size or composition of the subgroups, this limitation should be explicitly stated.

I do not recommend interpreting the results as representative of all Nepalese migrant workers without an explicit discussion of the sample composition and its implications for external validity.

Likewise, a cross-sectional review of Tables 1–3 should be conducted to ensure that each variable has a clearly identifiable denominator. When a variable was assessed only in a subgroup or has missing data, the authors should, when appropriate, use the presentation n/N (%) and indicate the corresponding denominator through the heading or a footnote.

5.2. Clarity in the Presentation of Family, Mental Health, and Disability Variables

Tables 2 and 3 should provide greater clarity regarding the populations to which the different variables apply. Some variables related to family members, migration, remittances, disability, or specific mental health characteristics may correspond only to certain participants or have denominators different from the total sample size.

Requested Action:

The authors should explicitly indicate the denominator used for each variable and explain in footnotes any difference from the total sample size. Likewise, the categories used in the tables should exactly match the operational definitions described in Methods.

When variables derived from mental health screening instruments are presented, the authors should clearly specify the cut-off points used and avoid having screening categories interpreted as clinical diagnoses.

5.3. Discordant Results and Presentation of Changes Between Rounds

The manuscript presents descriptive decreases in anxiety and depression, but simultaneously reports considerable levels of psychological distress and a substantial deterioration in certain life satisfaction indicators. This pattern requires a much more careful interpretation.

It is not appropriate to select only favorable indicators to construct a general narrative of improvement. The results should be presented jointly and considering the possible heterogeneity of the outcomes.

The authors should present and discuss jointly:

- anxiety;
- depression;
- psychological distress;
- life satisfaction;
- happiness; and
- social support.

The Discussion should explain possible reasons for the discrepancies, clearly differentiating between plausible hypotheses and mechanisms that have actually been demonstrated by the data.

Table 4 should also make explicit that the estimates obtained through GHQ-12 and HSCL-25 correspond to results from screening instruments and not necessarily to clinical

diagnoses. The cut-off points used should be clearly identified and supported by appropriate references.

Particular attention is required for Table 4a. The authors should clearly explain the differences in sample size between baseline, midline, and endline, specifying what population each denominator represents and why the number of participants differs between the different rounds.

If the three rounds correspond to independent samples, the presentation of the results should avoid suggesting that longitudinal follow-up of the same individuals was conducted.

In this context, the column called "Trend" should also be reconsidered. Expressions such as "increasing" or "decreasing" may lead to the results being interpreted as individual longitudinal changes. It would be preferable to use a descriptive and neutral label or remove this column.

Requested Action:

The authors should:

- clearly identify the denominator for each round;
- explain the differences in sample size between phases;
- transparently present the results for each phase;
- indicate whether the differences were subjected to statistical tests;
- consider presenting confidence intervals for the main estimates;
- avoid interpreting descriptive differences between independent samples as individual longitudinal changes; and
- revise the language used to describe differences between baseline, midline, and endline.

It is important to clarify that the fact that the samples are independent does not imply that statistical comparisons are impossible; the fundamental issue is that, in the absence of individual follow-up and an appropriate design for causal inference, the observed differences between rounds should not automatically be interpreted as effects attributable to the program.

5.4. Life Satisfaction, Happiness, and Data Availability

Table 5 requires greater transparency regarding the denominators and availability of information for each evaluation round. In particular, when the number of participants available for a variable at midline is substantially lower than at endline, the authors should

explain whether this is due to missing data, differences in instrument administration, or specific inclusion criteria.

The differences observed between rounds should be interpreted taking into account that the participants included in each phase may not be the same.

Requested Action:

The authors should provide complete denominators for each variable and category, explain losses or differences in data availability, and avoid presenting differences between rounds as evidence of individual deterioration or improvement when the same participants were not followed.

When appropriate, they should provide confidence intervals for the main estimates, particularly for results with a small number of events, in order to allow the reader to assess the precision of the estimates.

5.5. Qualitative Results and Causal Claims

Participant testimonies provide important information for understanding their experiences and may appropriately complement the quantitative results. However, qualitative data alone do not allow the effect of the program to be quantified or causality to be established.

For this reason, statements such as "directly prevented suicide attempts" or equivalent expressions should be carefully reviewed. When such statements come from participant testimonies, they should be presented as the participants' perceptions or attributions regarding the benefit of the support received, and not as evidence that the program directly prevented suicide attempts.

Requested Action:

The authors should clearly differentiate between:

1. experiences or perceptions reported by participants;
2. associations observed in the quantitative data; and causal effects or impact of the program.

Claims of population-level impact or effectiveness should be supported by a design and quantitative analysis that allow such inferences to be made. When this is not possible, the language should be limited to describing associations, descriptive changes, or participant perceptions.

6. DISCUSSION

6.1. Insufficiently Developed Discussion

The Discussion requires substantial restructuring.

Currently, a considerable part of the Discussion consists of repeating the main results and briefly associating them with previous literature.

For an article of this nature, the Discussion should interpret the results in depth and place them within the existing evidence.

Requested Action

The authors should reorganize the Discussion following, at minimum, this logic:

main finding, interpretation, comparison with previous studies, agreement/disagreement, possible mechanisms, context of Nepal, implications for intervention/policy, limitations, cautious interpretation.

6.2. Each Main Result Should Be Discussed Against the Evidence

The Discussion should explicitly address the main findings:

Anxiety and depression

The authors should compare the magnitude of the results with previous studies in:

- migrant workers;
- Nepalese population;
- migrant families;
- similar low- and middle-income contexts.

They should also incorporate relevant systematic reviews and meta-analyses.

Psychological distress

The increase in distress deserves specific discussion and cannot be subordinated to the favorable results for anxiety and depression.

Life satisfaction

The observed deterioration requires a much broader and balanced interpretation.

Requested Action

The authors should expand the Discussion of each main outcome and avoid a selective narrative focused exclusively on favorable results.

6.3. Interpretation of Mechanisms

The manuscript proposes explanations such as economic difficulties, job insecurity, and greater recognition of distress.

These explanations are plausible, but the study does not necessarily demonstrate them.

Requested Action

The authors should use explicitly hypothetical language:

- “may reflect”;
- “may be explained by”;
- “one possible explanation is…”;

and avoid presenting unmeasured mechanisms as established explanations.

6.4. Comparison with Systematic Reviews and Meta-analyses

The Discussion should make much better use of the available secondary literature.

Requested Action

The authors should incorporate and discuss relevant systematic reviews and meta-analyses, particularly when comparing:

- prevalences;
- intervention effects;
- psychosocial support;
- community interventions;
- stigma;
- mental health in migrant workers.

This is not simply about increasing the number of references, but about interpreting the findings of the present study against the accumulated body of evidence.

6.5. Limitations

The current limitations should be expanded.

They should explicitly include:

- cross-sectional design;
- independent samples across phases;
- absence of a control group;
- inability to establish causality;
- possible selection bias;

- sex imbalance;
- population heterogeneity;
- possible differences between rounds;
- limitations of the tools used;
- possible confounding factors;
- retrospective information, when applicable;
- limited generalizability.

Requested Action

The authors should expand the limitations section and explain how each limitation specifically affects the interpretation of the results, rather than simply listing them.

7. CONCLUSIONS

The current conclusion presents the program as if the results demonstrated that PARBARDHAN "effectively addressed" psychosocial well-being.

This exceeds what can reasonably be concluded from the described design.

Requested Action

The authors should reformulate the conclusion to indicate that the findings suggest potential benefits or are consistent with perceived benefits of the program, but that they do not allow causality to be established because of the design and the absence of a comparator group/individual follow-up.

They should also mention that the results were not uniformly favorable, particularly regarding psychological distress and life satisfaction.

8. ETHICS AND TRANSPARENCY

8.1. Ethical Approval

Action Required:

The authors should provide the exact date of NHRC approval (ref. 1088), confirm that it preceded recruitment and data collection, and provide supporting documentation.

8.2. Consent

The authors should expand the description of informed consent, particularly for adolescents.

8.3. Data Availability

The authors should review the data availability statement to ensure that it is consistent with PLOS policies.

If ethical or legal restrictions prevent the deposit of individual-level data, they should specifically explain:

- which data are restricted;
- what the ethical/legal basis is;
- who may request access;
- through what procedure;
- under what conditions access would be granted.

A generic "available upon request" statement should be avoided if it does not satisfy the applicable policies.

9. REFERENCES

9.1. Number and Coverage

The evaluated version contains a relatively limited bibliography for the number of concepts addressed.

I do not recommend that the authors artificially increase the number of references. The problem is insufficient coverage of the relevant evidence.

Requested Action

The authors should comprehensively review the references and incorporate current, high-quality literature on:

- migration and mental health;
- migrant workers;
- migrant families;
- MHPSS interventions;
- community interventions;
- peer support;
- stigma;
- systematic reviews;
- meta-analyses.

The central statements in the Introduction and Discussion should be supported by directly relevant references.

Recent Methodological Literature on the Assessment of Depressive and Anxiety Symptoms

In addition to expanding the epidemiological evidence and systematic reviews on mental health in migrant populations, it would be advisable for the authors to consider recent methodological literature related to the measurement of depressive and anxiety symptoms using widely used instruments in population research.

In particular, the authors should consider recent evidence on the psychometric properties, reliability, convergent validity, and measurement equivalence of these instruments. This is especially important when comparisons are made across populations, countries, sociodemographic groups, or different cultural contexts, since a difference in observed scores does not necessarily represent a real difference in the construct if the instrument does not have comparable measurement properties across groups.

As methodological literature that may be useful for contextualizing this aspect, the authors could consult, among others, the following recent works:

- Caldichoury N, Morales-Asencio B, Castellanos-Alvarenga LM, et al. (2025). *Factorial invariance of the Spanish version of the PHQ-9 by gender and country in Latin America and the Caribbean*. Frontiers in Psychiatry, 16, 1667612. https://doi.org/10.3389/fpsyt.2025.1667612
- Caldichoury N, Morales-Asencio B, Coronado J-C, et al. (2025). *Reliability and convergent validity of the PHQ-2 for the rapid detection of depressive symptoms in healthcare professionals in Argentina*. Frontiers in Psychiatry, 16, 1652072. https://doi.org/10.3389/fpsyt.2025.1652072
- López N, Coronado J-C, Ripoll-Córdoba D, et al. (2025). *Factorial invariance of the generalized anxiety disorder scale (GAD-7) in Latin America and the Caribbean*. Frontiers in Psychiatry, 16, 1529424. https://doi.org/10.3389/fpsyt.2025.1529424
- López N, Morales-Asencio B, Ripoll-Córdoba D, et al. (2025). *Internal validity and reliability of the GAD-7 test in Latin America*. Chronic Stress, 9, 24705470251315260. https://doi.org/10.1177/24705470251315260

The incorporation of this literature should be done selectively and only when conceptually relevant to the variables and instruments used in the present study. It is not recommended to incorporate these references solely to increase the number of citations, but rather to strengthen the methodological justification and interpretation of the mental health measurements.

9.2. Literature Update

Although older references may be appropriate when they constitute foundational works, the manuscript requires a substantial update of the recent literature.

Requested Action

The authors should update the bibliography with contemporary evidence, prioritizing recent and methodologically robust studies and reviews, without removing classic references that remain necessary.

9.3. Correspondence Between Claims and References

Some general statements regarding prevalence, effectiveness, mechanisms, and program benefits require a more direct correspondence with the cited evidence.

Requested Action

The authors should review each substantive statement in the Introduction and Discussion and verify that the cited reference actually supports the claim being made.

10. MAJOR COMMENTS — NECESSARY ACTIONS

I consider that the following points must be addressed in a major revision:

1. **Revise the title so that it accurately reflects the population and design.**
2. **Reformulate the abstract**, removing statements of effectiveness or causality that are not compatible with the design.
3. **Substantially restructure the Introduction**, expanding the conceptual and epidemiological framework and clearly defining the knowledge gap.
4. **Incorporate relevant secondary evidence**, including pertinent systematic reviews and meta-analyses.
5. **Fully clarify the design**, particularly the meaning of "retrospective elements".
6. **Demonstrate how baseline, midline, and endline were compared** and acknowledge that independent samples do not allow inference of individual changes.
7. **Present the composition of each study population** and justify the combination of heterogeneous groups.
8. **Strengthen the statistical methodology**, specifying tests, effect estimates, confidence intervals, missing data, and sensitivity analyses.

9. **Strengthen the description of the qualitative component and the integration of mixed methods.**
10. **Reformulate statements regarding suicide and effectiveness**, particularly those derived from qualitative testimonies.
11. **Substantially reconstruct the Discussion**, confronting each main result with the literature and explicitly addressing discordant results.
12. **Expand the limitations**, particularly those related to causality, independent samples, selection, and population composition.
13. **Reformulate the conclusions** so that they are proportional to the design and evidence.
14. **Document the ethical approval clearly**, including the exact date and its temporal relationship to the start of the research.
15. **Completely review the data availability statement** according to PLOS policies.
16. **Update and strengthen the references**, prioritizing relevant evidence and evidence syntheses.

11. MINOR COMMENTS

1. Conduct a complete linguistic review of the manuscript.
2. Standardize the terminology used for "mental health", "psychosocial wellbeing", "psychological distress", "anxiety", and "depression".
3. Review the consistency between title, abstract, objectives, methods, results, and conclusions.
4. Verify all denominators and percentages in the tables.
5. Ensure that each table can be interpreted independently.
6. Define all abbreviations the first time they appear.
7. Review consistency in the presentation of units, percentages, and statistical values.
8. Verify that all results mentioned in the text can be directly located in the corresponding tables or figures.
9. Review that all references cited in the text appear in the bibliography and vice versa.
10. Review the supplementary material and ensure that the ethical and methodological documents cited are actually available
