# Supplementary material for: Mental health and psychosocial wellbeing among beneficiaries of a community-based intervention in Nepal: Findings from a cross-sectional endline evaluation of the PARBARDHAN project
Source: PLOS Glob Public Health. 2026 Oct 16;6(10):e0006666. doi: 10.1371/journal.pgph.0006666 (PMC13674775; doi:10.1371/journal.pgph.0006666)
Supplement: Supplementary file 3 [file pgph.0006666.s001.docx]

**My Comments After Reviewing The Paper**

This manuscript presents findings from the endline evaluation of the PARBARDHAN project, a community-based mental health and psychosocial support (MHPSS) intervention for migrant workers and their left-behind families in Nepal. The mixed-methods design is appropriate, and the topic is highly relevant given the scale of labour migration from Nepal. However, several methodological and interpretative issues need to be addressed before publication can be recommended.

**Major Concerns:**

1. **Temporal mismatch in data collection:** The manuscript states quantitative data were collected in February 2026 (Page 5, line 25). As we are currently in 2026, this suggests either a typographical error or a serious anachronism. If data have not yet been collected, the study cannot be an endline evaluation. This requires immediate clarification.
2. **Contradictory interpretation of psychological distress:** The authors report a rise in psychological distress (from 20.4% at baseline to 34.7% at endline) yet frame this as project effectiveness. While they suggest increased awareness may lead to higher reporting, the discussion fails to adequately reconcile why a successful intervention would show worsening distress alongside improved anxiety/depression. This needs more complexed interpretation, including possible external stressors (e.g., economic downturn, post-migration challenges).
3. **Life satisfaction reversal unexplained:** The dramatic decline in life satisfaction from ~80% positive at midline to >88% dissatisfied at endline is concerning. The qualitative explanation (job insecurity, debt) is plausible, but the manuscript does not clarify whether these economic factors worsened during the project period or if the midline sample was systematically different. Without this, the project’s net impact remains unclear.
4. **Sampling and generalizability:** The sample is 94% female, yet migration in Nepal is predominantly male. While left-behind families are often female, the near-absence of male returnee migrants limits the ability to draw conclusions about migrant workers themselves. This should be acknowledged as a major limitation.

**Minor Concerns:**

- Abstract and results sections should consistently report that psychological distress increased, not just that challenges were "highlighted."
- Table 5 (life satisfaction) references midline data but no midline n or statistical comparison is provided.

**Conclusion:** The study offers valuable qualitative insights into stigma reduction and peer support, but quantitative claims of effectiveness are undermined by contradictory distress and life satisfaction trends, as well as potential data collection timeline issues. Major revisions are required.
