# Supplementary material for: Mental health and psychosocial wellbeing among beneficiaries of a community-based intervention in Nepal: Findings from a cross-sectional endline evaluation of the PARBARDHAN project
Source: PLOS Glob Public Health. 2026 Oct 16;6(10):e0006666. doi: 10.1371/journal.pgph.0006666 (PMC13674775; doi:10.1371/journal.pgph.0006666)
Supplement: Supplementary file 4 [file pgph.0006666.s003.docx]

Response to Editorial Office – Resubmission of PGPH-D-26-01321

Dear Editors,

Thank you for your request.

This manuscript is a resubmission of the accidentally withdrawn submission PGPH-D-26-01321. The previous submission was withdrawn by the author in error before it entered peer review. No editorial decision or reviewer comments were ever issued.

Therefore, no changes have been made to the manuscript itself as there were no reviewer or editor comments to address.

However, the following administrative corrections have been made to the submission record to comply with PLOS policies:

- Financial disclosure statement added
- Competing interests statement added
- Data availability statement added

The manuscript file remains unchanged from the original submission.

We respectfully submit this manuscript for your consideration and apologize again for the administrative error.

Sincerely,

Dr. Ganesh Bhandari
