# Supplementary material for: Mental health and psychosocial wellbeing among beneficiaries of a community-based intervention in Nepal: Findings from a cross-sectional endline evaluation of the PARBARDHAN project
Source: PLOS Glob Public Health. 2026 Oct 16;6(10):e0006666. doi: 10.1371/journal.pgph.0006666 (PMC13674775; doi:10.1371/journal.pgph.0006666)
Supplement: Supplementary file 5 [file pgph.0006666.s004.docx]

**Response to Reviewers**

Dear Editor,

We are grateful to the Editor and all four reviewers for their thorough and constructive assessment of our manuscript. All concerns have been addressed and are detailed below. Changes in the revised manuscript are shown as tracked changes. Each reviewer comment is presented in italics, followed by our response and a description of specific changes made.

**EDITOR'S COMMENTS**

**Major Concern 1 — Temporal mismatch: February 2026**

**Reviewer:** *The manuscript states quantitative data were collected in February 2026. As we are currently in 2026, this suggests a typographical error or serious anachronism requiring immediate clarification.*

**Response:** This is a typographical error. Data were collected from 3–26 February 2025, not 2026. This has been corrected at all instances throughout the manuscript. The endline evaluation was conducted in February 2025, consistent with the project period (January 2022–December 2025) and NHRC ethical approval (Ref. 1088) obtained prior to data collection. We sincerely apologise for this error.

**Major Concern 2 — Contradictory interpretation of psychological distress**

**Reviewer:** *The authors report a rise in psychological distress (20.4% to 34.7%) yet frame this as project effectiveness. The discussion fails to adequately reconcile why a successful intervention would show worsening distress alongside improved anxiety/depression. This needs more complex interpretation, including possible external stressors (e.g. economic downturn, post-migration challenges).*

**Response:** We fully accept this concern. The Discussion has been substantially revised. We now present two distinct evidence-based explanatory mechanisms: (1) The GHQ-12 is a broad screening instrument sensitive to short-term situational stressors including financial strain and economic uncertainty, capturing a wider spectrum of distress than the symptom-specific HSCL-25 subscales. Persistent economic hardship and migration-related stressors continued to generate transient distress even as clinical anxiety and depression were addressed through project interventions — consistent with South Asian evidence that financial stress is an independent driver of general psychological distress. (2) The project's sustained awareness-raising activities may have increased participants' willingness to acknowledge distress, a phenomenon documented in similar MHPSS evaluations where baseline underreporting due to stigma is common. Critically, the manuscript no longer frames the increase in psychological distress as evidence of project effectiveness. Language throughout has been moderated, and structural determinants of distress are explicitly discussed.

**Major Concern 3 — Life satisfaction reversal unexplained**

**Reviewer:** *The dramatic decline in life satisfaction from ~80% positive at midline to >88% dissatisfied at endline is concerning. The manuscript does not clarify whether economic factors worsened during the project period or if the midline sample was systematically different. Without this, the project's net impact remains unclear.*

**Response:** We fully accept this concern. The Discussion has been revised to address this more rigorously. We now explicitly acknowledge: (1) we do not have concurrent longitudinal economic indicator data to demonstrate directly whether economic conditions worsened during the project period — this is clearly stated as a study limitation; (2) the midline (n=68) and endline (n=245) life satisfaction samples were independent cross-sectional samples of different sizes drawn from overlapping but non-identical populations, meaning systematic differences between samples cannot be ruled out as a contributing factor; (3) qualitative evidence consistently identifies financial insecurity, remittance uncertainty, and debt as primary drivers of dissatisfaction, providing plausible contextual explanation, but causal attribution is not possible given the cross-sectional design. A recommendation for future evaluations to collect concurrent economic and social indicator data alongside psychosocial measures has been added to the conclusion.

**Major Concern 4 — Sampling and generalizability**

**Reviewer:** *The sample is 94% female, yet migration in Nepal is predominantly male. While left-behind families are often female, the near-absence of male returnee migrants limits the ability to draw conclusions about migrant workers themselves. This should be acknowledged as a major limitation.*

**Response:** We fully accept this as a major limitation. The Results, Discussion, Limitations, and Conclusion sections now all contain explicit statements that the 94.3% female sample — while reflecting daytime household availability of left-behind family members in high male out-migration districts — means that male returnee migrants are almost entirely unrepresented in the quantitative findings. Claims regarding the psychosocial wellbeing of migrant workers themselves are explicitly qualified throughout, and the limitations paragraph specifically recommends future evaluations employ strategies such as evening or weekend fieldwork and targeted recruitment of returnee migrants to ensure adequate male representation.

**Minor Concerns — Transparent reporting and Table 5**

**Reviewer:** *Abstract and results sections should consistently report that psychological distress increased. Table 5 references midline data but no midline n or statistical comparison is provided.*

**Response:** The abstract and results sections have been revised to explicitly report the increase in psychological distress (20.4% to 34.7%) alongside the reductions in anxiety (37.2% to 8.2%) and depression (26.2% to 5.3%). Midline sample sizes have been added to Table 5 (life satisfaction: midline n=68; happiness: midline n=91). A note beneath each table clarifies that formal statistical comparison is not appropriate given the independent cross-sectional samples at each evaluation phase, and that baseline data for these instruments were not available.

**REVIEWER 1 COMMENTS**

**Abstract — Five sub-points**

**Reviewer:** *Introduction and objective insufficiently aligned with title and aims. Methods section should state specific assessment tools. Key prevalence estimates from baseline and midline should be included. Age range of participants should be stated. Conclusion appears abrupt and insufficiently grounded in preceding findings.*

**Response:** All five sub-points have been addressed: (1) The opening sentence and study objective have been revised to reflect the longitudinal evaluative nature (baseline–midline–endline comparison); (2) specific validated instruments are now named — GHQ-12 (psychological distress), HSCL-25 (anxiety and depression), 6-item Life Satisfaction Tool, and 25-item Happiness Tool — with domains stated; (3) baseline prevalence figures (distress 20.4%, anxiety 37.2%, depression 26.2%) and reference to midline improvements have been incorporated; (4) participant age range (16–80 years) has been added; (5) the conclusion has been substantially revised to explicitly connect endline findings to the trajectory of change observed across all three assessment phases.

**Title — Restructuring**

**Reviewer:** *The title may benefit from minor restructuring to better reflect the primary aim of the study.*

**Response:** The title has been revised to: 'Changes in Psychosocial Wellbeing Among Migrant Workers and their Left-Behind Families in Nepal: Evidence from a Longitudinal Evaluation of the PARBARDHAN Community-Based Intervention.' This revision explicitly signals: (a) the outcome of interest (psychosocial wellbeing), (b) the study population, (c) the country context, (d) the evaluative/longitudinal nature, and (e) the community-based intervention being evaluated.

**Introduction — Multiple issues**

**Reviewer:** *More detailed discussion of PARBARDHAN project needed. Rationale for selecting Salyan and Kailali districts should be justified. Baseline and midline prevalence estimates should be provided. Additional recent literature needed. Sparse and inconsistent citation practices.*

**Response:** A substantially expanded description of PARBARDHAN is now included: full English name (EMHPW-MW) and its relationship to the community name PARBARDHAN clarified; project period (January 2022–December 2025); implementing partner (CMC-Nepal) and financial partner (Felm); target beneficiaries; geographic coverage; and key intervention components (SHGs, peer support networks, counselling, mhGAP-based health worker training, primary care integration, local government engagement). District selection is explicitly justified — Salyan and Kailali are among Nepal's highest labour out-migration districts. Baseline (distress 20.4%, anxiety 37.2%, depression 26.2%) and midline figures are now stated in the introduction. Additional recent literature has been incorporated throughout. The statement at line 63 on higher mental health rates among migrant workers has been expanded and precisely referenced including Hasan et al. (2021), a systematic review and meta-analysis.

**Methods — Four issues**

**Reviewer:** *Opening statement should be restructured. Alignment with GRAMMS guideline encouraged. Greater sampling detail needed. Clarification on inclusion criteria, age eligibility, and tool suitability.*

**Response:** The study design statement has been substantially restructured: the revised text specifies the cross-sectional mixed-methods design, rationale for integration, point of integration (data interpretation), limitation of quantitative component addressed by qualitative inquiry, and retrospective comparison with baseline and midline. Reporting is explicitly aligned with the GRAMMS guideline. Sampling procedures are fully described: quantitative (simple random sampling from CMC-Nepal beneficiary list using random number generator, with random replacement for unavailable/non-consenting participants); qualitative (purposive sampling until inductive thematic saturation per Saunders et al., 2018, assessed iteratively after each KII/FGD). Formal eligibility criteria now stated: PARBARDHAN beneficiary status (direct/indirect), age ≥15 years, project municipality residency, defined beneficiary group membership, and capacity to consent. Age threshold of 15 years is justified in relation to project beneficiary definitions; cautionary note on GHQ-12/HSCL-25 validation for adolescents added.

**Results — Multiple issues**

**Reviewer:** *Female sample dominance — potential selection bias. One participant below 18. Subgroup analysis of migrant workers vs left-behind families. Table 5 midline n missing. Statistical significance tests needed. Overall analytical rigour concerns.*

**Response:** An explanatory paragraph has been added addressing the female dominance: daytime household data collection in districts where male out-migration is predominant results in disproportionate female representation, mirroring the actual beneficiary population composition. The 17-year-old participant with physical disability is now identified with eligibility justification, assent/consent procedure, and acknowledgement as a minor limitation. Absence of subgroup disaggregation by beneficiary category is acknowledged as a limitation with a recommendation for future evaluations. A three-phase comparison table (Table X) has been added presenting distress, anxiety, and depression for baseline, midline, and endline with instruments and sample sizes. Midline N added to Table 5. The methodological inappropriateness of formal inferential tests across independent cross-sectional samples is explicitly explained and justified; comparisons are presented descriptively.

**Discussion — Strengthening**

**Reviewer:** *Thematic interpretation requires clearer structure and analytical depth. Greater comparison with regional and international studies. Insufficiently supported claims. Need for stronger scientific framing and critical interpretation.*

**Response:** The Discussion has been substantially expanded with: clearer thematic structure with each major finding in a dedicated analytically coherent paragraph; comparative evidence from Hoeft et al. (systematic review, community-based MHPSS in LMICs), Jordans et al. (Nepal-based RCT), Cochrane review evidence on peer support, South Asian literature on left-behind spouse life satisfaction and disability certification barriers; theoretical framing using the socio-ecological model and Link and Phelan's structural vs individual stigma dimensions; critical explanation of divergent GHQ-12 vs HSCL-25 findings; explicit contextualisation of life satisfaction decline; and stronger critical interpretation throughout.

**Conclusion — Cautious framing**

**Reviewer:** *Conclusions appear somewhat overstated relative to the analyses presented.*

**Response:** The conclusion has been substantially revised. Causal language has been replaced with appropriately hedged framing ('suggests that... may contribute to'). The cross-sectional independent-sample design limitation, contrasting findings (increased distress, declining life satisfaction), and predominantly female sample are all explicitly acknowledged. Recommendations are grounded in actual findings and include livelihood integration, strengthened referral systems, disability certification advocacy, sustained anti-stigma efforts, and adoption of longitudinal cohort designs for future evaluations.

**Minor Concerns — Syntax, grammar, spelling, flow, referencing**

**Reviewer:** *Syntax and grammatical inconsistencies require attention. Spelling errors noted. Improved flow and transitions needed. Citation formatting should be standardised.*

**Response:** The manuscript has been thoroughly proofread: grammar, spelling, punctuation, and tense consistency corrected throughout; transitional sentences added between paragraphs; all references reviewed with journal titles added where missing; citation formatting standardised to a consistent style including author names, year, title, journal, volume, issue, and pages for all journal articles.

**REVIEWER 2 COMMENTS**

**Abstract — Analyses description and actual changes not described**

**Reviewer:** *Please add a description of which analyses you did — this is more important than which software you used. You write 'These outcomes reflect changes' but don't describe the actual changes in the results.*

**Response:** The abstract methods paragraph now describes the analytical approach rather than the software: quantitative data were analysed using descriptive statistics (frequencies, means, proportions) and bivariate analyses, with retrospective comparison to baseline and midline; qualitative data were analysed using framework analysis. The vague sentence has been replaced with explicit figures: anxiety declined from 37.2% (baseline) to 8.2% (endline), depression from 26.2% to 5.3%, while psychological distress increased from 20.4% to 34.7%.

**Introduction — Return migration figures; EMHPW-MW/PARBARDHAN relationship; project interventions; baseline indicators**

**Reviewer:** *How many Nepalis return from abroad each year? It is not clear how the EMHPW-MW and PARBARDHAN projects relate. Please also describe what PARBARDHAN actually did. You state endline evaluation allowed comparison with baseline indicators but don't describe any quantitative baseline indicators.*

**Response:** (1) Precise national-level data on annual return migration volumes are not consistently published in the available literature; a contextually grounded statement on reintegration challenges (unemployment, debt, psychosocial distress) has been added instead. (2) The relationship between names is now explicitly clarified in the introduction: PARBARDHAN is the Nepali community name for the project formally titled EMHPW-MW in English — they are the same project. (3) Key intervention components are now described in detail: SHG/peer support network formation, psychosocial counselling, awareness campaigns, mhGAP-based health worker training, MHPSS integration into primary healthcare, and local government engagement. (4) Baseline prevalence estimates for psychological distress (GHQ-12: 20.4%), anxiety (HSCL-25: 37.2%), and depression (HSCL-25: 26.2%) are now explicitly stated in the introduction with the measuring instruments named.

**Methods — Sampling, comparability, saturation, tool validation, FGD guideline, venue, data collection location, statistical analysis, baseline comparison, thematic analysis type**

**Reviewer:** *Multiple methods clarifications requested across sampling, saturation, tool validation, terminology, and analysis.*

**Response:** All points addressed: (1) Quantitative sampling: simple random sampling from CMC-Nepal beneficiary list using random number generator with random replacement; qualitative: purposive sampling. (2) Independent cross-sectional samples at each evaluation phase — explicitly stated with comparability limitations noted. (3) Saturation operationalised per Saunders et al. (2018) as inductive thematic saturation, assessed iteratively after each KII/FGD within each participant category. (4) GHQ-12 and HSCL-25 validated for Nepali/South Asian populations (referenced); Happiness and Life Satisfaction Tools locally validated by CMC-Nepal. (5) 'FGD guideline' replaced with 'semi-structured FGD discussion guide' with topic domains described. (6) KIIs conducted at municipal offices/health facility premises; FGDs at community gathering venues (SHG meeting spaces, community halls). (7) Quantitative data collected through face-to-face structured household interviews. (8) Scores normally distributed; means ± SD reported alongside proportions exceeding cut-off thresholds. (9) Three-phase comparison table (Table X) added presenting all evaluation phases. (10) Framework analysis specified with five stages: familiarisation, thematic framework identification, indexing, charting, mapping and interpretation — with deductive and inductive logic described and inter-rater consistency procedures noted.

**Results — Female dominance, table information, baseline presentation, qualitative themes, interpretation, KII/IDI discrepancy**

**Reviewer:** *Nearly all participants female. Tables need more information. Baseline results not presented. All themes should be stated at beginning of qualitative section. Themes underdeveloped in favour of quotes. Quote attribution error (KII vs IDI).*

**Response:** (1) Female dominance explained in results section — daytime household visits in high male out-migration setting. (2) All tables now include total N, instrument name, and cut-off thresholds. (3) Table X added presenting distress, anxiety, depression across all three phases; midline N added to Tables 10/11; baseline unavailability noted for life satisfaction/happiness. (4) Summary paragraph added at opening of Section 3.2 listing all eight major themes. (5) All thematic sections revised to lead with analytical interpretation before quotes; number of quotes per subtheme reduced; quotes embedded within analytical text. (6) The quote beginning 'Significant changes have occurred...' is confirmed as IDI, Ghodaghodi — the erroneous KII label corrected throughout.

**Discussion — Strengths and limitations**

**Reviewer:** *Please add a paragraph on strengths and limitations of your study.*

**Response:** A dedicated strengths and limitations paragraph has been added as a new subsection before the conclusion. Strengths: mixed-methods triangulation; geographic breadth across seven municipalities; locally validated and internationally recognised instruments; inclusive sampling of marginalised groups; multi-phase data enabling trend analysis. Limitations: independent cross-sectional samples precluding causal inference; no control group; predominantly female sample limiting generalisability; absence of beneficiary category disaggregation; missing baseline data for two instruments; potential social desirability bias; risk of analytical bias given evaluation team's project involvement.

**References — Missing journal titles**

**Reviewer:** *Most of the references lack journal titles, please add.*

**Response:** All references have been reviewed and journal titles added where missing. The full reference list has been standardised to a consistent format including author names, year, title, journal title, volume, issue, and page numbers for all journal articles.

**General — Proofreading**

**Reviewer:** *Please proof-read the article carefully, currently there are multiple typos and grammatical errors.*

**Response:** The manuscript has been thoroughly proofread. Corrections include: consistent punctuation after introductory clauses; correction of run-on sentences and fragments; standardisation of hyphenation for compound modifiers; correction of subject-verb agreement; standardisation of tense (past tense for methods/results, present tense for discussion/implications); removal of redundant phrases; correction of spelling errors; standardisation of number reporting conventions; and correction of the KII/IDI attribution error.

**REVIEWER 3 COMMENTS**

**Critical Concern 1 — Data collection date**

**Reviewer:** *The manuscript reports quantitative data collected in February 2026. As this date is in the future relative to the current submission, a serious error appears present.*

**Response:** This is a typographical error. Data were collected from 3–26 February 2025. Corrected at all instances throughout the manuscript. The error arose from a transcription mistake during preparation and does not reflect any issue with the actual study timeline.

**Critical Concern 2 — Contradictory outcomes interpretation**

**Reviewer:** *Psychological distress increased from 20.4% to 34.7% yet the authors frame this as project effectiveness. Life satisfaction collapsed from ~80% positive at midline to >88% dissatisfied — attributed vaguely to economic insecurity without demonstrating whether such factors changed during the project period.*

**Response:** We fully accept both points. For psychological distress: the Discussion now presents two evidence-based mechanisms — (1) persistent economic stress captured by the broad GHQ-12 vs the symptom-specific HSCL-25, and (2) increased detection sensitivity following awareness-raising. The manuscript no longer frames increased distress as effectiveness evidence. For life satisfaction: we explicitly acknowledge that concurrent economic indicator data are not available, that midline and endline comprised different-sized independent samples, and that causal attribution is not possible. These are stated as study limitations with recommendations for future evaluations.

**Critical Concern 3 — Female sample and migrant worker claims**

**Reviewer:** *The sample is 94% female. The near-absence of male returnee migrants severely limits claims about 'migrant workers' themselves. This limitation must be explicitly stated.*

**Response:** Explicit statements have been added throughout — in Results, Discussion, Limitations, and Conclusion — that the predominantly female sample means male returnee migrants are almost entirely unrepresented in the quantitative findings. Claims about migrant workers themselves are explicitly qualified. Future evaluation recommendations include targeted male recruitment strategies.

**Minor Concerns**

**Reviewer:** *Abstract and results should report distress increase transparently. Table 5 references midline data without midline n or statistical comparison.*

**Response:** Abstract and results now explicitly report distress increased from 20.4% to 34.7%. Midline N added to Table 5 (life satisfaction: n=68; happiness: n=91). Statistical comparison note clarifies descriptive-only presentation given independent cross-sectional samples.

**REVIEWER 4 COMMENTS**

**Comment 1 — Punctuation and grammar**

**Reviewer:** *Punctuation and grammar are very important for effective communication; greater attention should be paid to their correct use.*

**Response:** The entire manuscript has been thoroughly proofread. Corrections include: consistent comma use after introductory clauses and within lists; correction of run-on sentences; standardisation of hyphenation; correction of apostrophe and quotation mark usage; standardisation of verb tense; correction of subject-verb agreement; removal of redundant phrasing; and correction of spelling errors throughout.

**Comment 2 — Sample size formula**

**Reviewer:** *It is highly important to clearly indicate the formula used in determining the sample size.*

**Response:** The sample size formula is now explicitly presented in the Methods section: n = Z²pq/d², where Z = 1.96 (95% confidence level), p = 13.2% (midline depression prevalence), q = 1 − p = 86.8%, and d = 0.05 (margin of error), yielding a minimum of 177 participants. Adding a 20% non-response rate gave an estimated requirement of 212, and 245 participants were ultimately enrolled, exceeding the minimum.

**Comment 3 — Standard deviation notation**

**Reviewer:** *The standard deviation should be appropriately reported using the '±' notation to reflect variability around the mean.*

**Response:** All standard deviation values throughout the manuscript have been revised to use ± notation consistently. For example, mean age is now reported as 38.16 ± 12.12 years (range 16–80). This formatting has been applied to all descriptive statistics reporting means with standard deviations across the results section.

**SUMMARY OF ALL REVISIONS**

The table below summarises all major revisions made to the manuscript in response to reviewer and editor comments.

| **Section** | **Issue Raised** | **Action Taken** |
| --- | --- | --- |
| Title | Did not reflect evaluative/longitudinal nature | Revised to reflect longitudinal evaluation and community-based intervention |
| Abstract | Missing tools, age range, baseline figures, vague changes, overconfident conclusion, date error | Fully revised: tools named, age added, baseline/midline figures, direction of change explicit, cautious conclusion, date corrected to 2025 |
| Introduction | EMHPW-MW/PARBARDHAN confusion; project description lacking; no district rationale; no baseline figures; weak citations | Naming clarified; project description expanded; district rationale added; baseline figures stated; literature strengthened |
| Methods | Vague design statement; no GRAMMS; insufficient sampling; no eligibility criteria; no tool validation; FGD terminology; no venue info; no formula; no saturation operationalisation; thematic analysis type unspecified | GRAMMS alignment; eligibility criteria added; formula stated (Z²pq/d²); saturation per Saunders et al. 2018; tool validation noted; venues described; framework analysis specified |
| Results | Female dominance unexplained; minor participant; no subgroup analysis; no baseline comparison table; missing midline N; no statistical tests justified; themes unlisted; quote-heavy; KII/IDI error | All addressed: explanation; participant noted; limitation stated; comparison table added; N added; design limitation explained; 8 themes listed; interpretation strengthened; IDI label corrected |
| Discussion | Contradictory findings unexplained; life satisfaction unexplained; insufficient literature; no strengths/limitations; weak framing | Substantially expanded; two mechanisms for distress; life satisfaction contextualised; comparative literature added; strengths/limitations paragraph added; theoretical framing included |
| Conclusion | Overstated relative to evidence | Revised with cautious hedged language; limitations acknowledged; balanced and grounded in actual findings |
| References | Missing journal titles; inconsistent formatting | Journal titles added; formatting standardised throughout |
| Language/SD/Formula | Grammar, spelling, punctuation, ± notation missing, formula not stated | Full proofread; ± notation applied throughout; formula explicitly stated |

We believe these comprehensive revisions have substantially strengthened the manuscript and addressed all concerns raised. We hope the revised manuscript meets the standards required for publication and welcome any further guidance from the Editor.

Sincerely,

Himal Gaire and Co-authors

Centre for Mental Health and Counselling Nepal (CMC-Nepal)

Corresponding author: Himal.gaire@cmcnepal.org
