# Supplementary material for: Mental health and psychosocial wellbeing among beneficiaries of a community-based intervention in Nepal: Findings from a cross-sectional endline evaluation of the PARBARDHAN project
Source: PLOS Glob Public Health. 2026 Oct 16;6(10):e0006666. doi: 10.1371/journal.pgph.0006666 (PMC13674775; doi:10.1371/journal.pgph.0006666)
Supplement: Supplementary file 6 [file pgph.0006666.s005.docx]

**RESPONSE TO REVIEWERS**

Manuscript ID: PGPH-D-26-01439R1

Title: Changes in Psychosocial Wellbeing Among Migrant Workers and their Families in Nepal: Evidence from a Longitudinal Evaluation of the PARBARDHAN Community-Based Intervention

Journal: PLOS Global Public Health

Date: 06 June 2026

Dear Editor,

We thank the Editor for the opportunity to revise our manuscript (PGPH-D-26-01439R1). We note that the manuscript was returned for revision without new reviewer comments. Upon careful review of our first revision against the original reviewer concerns and our response letter, we identified several instances where changes promised in the response letter were either not fully implemented or not correctly applied in the submitted manuscript. We sincerely apologise for these inconsistencies.

This second revision addresses all of these gaps. Below we: (1) summarise the additional corrections made in this revision, and (2) provide the complete point-by-point response to all four reviewers and the Editor for reference. All changes are visible in the Revised Manuscript with Track Changes file.

1. Conclusion — Not revised as promised

Additional Correction in This Revision: The response letter stated the conclusion was 'substantially revised with cautious hedged language,' but the original conclusion remained unchanged in the submitted manuscript. The conclusion has now been fully revised to: (a) replace causal language with appropriately hedged framing ('suggests that... may contribute to'); (b) explicitly acknowledge the cross-sectional independent-sample design limitation; (c) acknowledge the predominantly female sample as limiting generalisability to male migrants; (d) balance positive and negative findings; and (e) provide grounded recommendations for future programming and evaluation design.

2. Three-phase comparison table (Table 4a) — Missing

Additional Correction in This Revision: The response letter stated a three-phase comparison table had been added presenting distress, anxiety, and depression for baseline, midline, and endline. This table was absent from the submitted manuscript. Table 4a has now been inserted in Section 3.1, presenting all three outcomes across all three evaluation phases with sample sizes, instruments, cut-off thresholds, and a trend indicator column. An explanatory note beneath the table clarifies the independent cross-sectional sampling design and the descriptive nature of comparisons.

3. Table 5 — Midline sample size (N) missing

Additional Correction in This Revision: The response letter stated midline N had been added to Table 5 (life satisfaction: n=68; happiness: n=91). The midline column in Table 5 did not include sample sizes. This has been corrected — the column headers now read 'Endline (n=245)' and 'Midline (n=68)' for life satisfaction, with the happiness section updated accordingly.

4. Abstract — Software statement retained instead of replaced

Additional Correction in This Revision: The response letter stated the abstract methods sentence was revised to describe analytical approaches rather than software. The original sentence ('Data analysis was performed using SPSS v26 for quantitative data and NVivo 12 for qualitative data') remained in the submitted version. This has been corrected: the abstract now states — 'Quantitative data were analysed using descriptive statistics (frequencies, means, proportions) and bivariate analyses, with retrospective comparison to baseline and midline findings. Qualitative data were analysed using framework analysis to identify themes related to project relevance, implementation, and impact.'

5. Typographical errors — Not corrected despite promised proofread

Additional Correction in This Revision: Several typographical and grammatical errors identified in the original manuscript were not corrected in the first revision despite a full proofread being promised. The following have now been corrected: (a) 'formally totled' corrected to 'formally titled'; (b) duplicate age range '(aged 16-80)' removed from abstract; (c) incomplete sentence 'using simple random sampling using.' corrected; (d) 'society/country' replaced with 'communities'; (e) 'during the experiment' replaced with 'during the project period'; (f) 'showed strong programmatic achievement' replaced with more precise language; (g) 'from 4 to 19 health facilities till midline' revised to 'rising to 19 by midline'; (h) inconsistent article use and punctuation corrected throughout the abstract and methods.

6. PARBARDHAN/EMHPW-MW naming — Insufficiently clarified

Additional Correction in This Revision: The response letter promised an explicit clarification of the relationship between PARBARDHAN and EMHPW-MW. The introduction still read 'PARBARDHAN project-formally totled' without clear explanation. This has been corrected to: 'the PARBARDHAN project — formally titled "Enhancing Mental Health and Psychosocial Wellbeing of Migrant Workers and their Families" (EMHPW-MW) in English, and known locally in Nepali communities as PARBARDHAN.'

**EDITOR'S COMMENTS**

Major Concern 1 — Temporal mismatch: February 2026

Previous Reviewer Concern: The manuscript states quantitative data were collected in February 2026. This suggests a typographical error or serious anachronism requiring immediate clarification.

Response/Action: Confirmed typographical error. Data were collected from 3–26 February 2025. Corrected throughout the manuscript. The endline evaluation was conducted in February 2025, consistent with the project period (January 2022–December 2025) and NHRC ethical approval (Ref. 1088). This correction was implemented in the first revision and is confirmed in this revision.

Major Concern 2 — Contradictory interpretation of psychological distress

Previous Reviewer Concern: The authors report a rise in psychological distress (20.4% to 34.7%) yet frame this as project effectiveness. The discussion fails to adequately reconcile why a successful intervention would show worsening distress alongside improved anxiety/depression.

Response/Action: Fully accepted and addressed. The Discussion presents two evidence-based explanatory mechanisms: (1) the GHQ-12 captures a broader spectrum of situational and financial distress than the symptom-specific HSCL-25; persistent economic hardship and migration-related stressors continued independently of project interventions; (2) increased mental health literacy through awareness-raising may have improved willingness to acknowledge and report distress. The manuscript no longer frames the distress increase as project effectiveness. Structural determinants of distress are explicitly discussed. This was implemented in the first revision and confirmed in this revision.

Major Concern 3 — Life satisfaction reversal unexplained

Previous Reviewer Concern: The dramatic decline in life satisfaction from ~80% positive at midline to >88% dissatisfied at endline. The manuscript does not clarify whether economic factors worsened or if the midline sample was systematically different.

Response/Action: Fully accepted and addressed. The Discussion now explicitly acknowledges: (1) absence of concurrent economic indicator data preventing direct demonstration of economic change — stated as a study limitation; (2) the midline (n=68) and endline (n=245) samples were independent with different sizes, meaning compositional differences cannot be ruled out; (3) qualitative evidence identifies financial insecurity and debt as plausible drivers but causal attribution is not possible. This was implemented in the first revision and confirmed in this revision.

Major Concern 4 — Sampling and generalizability (94% female)

Previous Reviewer Concern: The sample is 94% female, yet migration in Nepal is predominantly male. The near-absence of male returnee migrants limits conclusions about migrant workers themselves.

Response/Action: Fully accepted as a major limitation. Explicit statements added throughout Results, Discussion, Limitations, and Conclusion that the predominantly female sample means male returnee migrants are almost entirely unrepresented. Claims about migrant workers are qualified throughout. Future evaluation recommendations include targeted male recruitment strategies. This was implemented in the first revision and the revised Conclusion in this revision further strengthens this acknowledgement.

Minor Concerns — Transparent reporting and Table 5

Previous Reviewer Concern: Abstract and results should consistently report that psychological distress increased. Table 5 references midline data but no midline N or statistical comparison is provided.

Response/Action: Abstract and results now explicitly report distress increased from 20.4% to 34.7%. Midline N has been added to Table 5 column headers in this revision (Endline n=245; Midline n=68 for life satisfaction). Statistical comparison note clarifies descriptive-only presentation given independent cross-sectional samples.

**REVIEWER 1 COMMENTS**

Abstract — Five sub-points (tools, age range, baseline figures, conclusion)

Previous Reviewer Concern: Introduction and objective insufficiently aligned. Methods should state specific tools. Baseline/midline prevalence needed. Age range needed. Conclusion abrupt.

Response/Action: All five sub-points addressed in first revision and confirmed: (1) objective revised to reflect longitudinal evaluative nature; (2) GHQ-12, HSCL-25, Life Satisfaction Tool, Happiness Tool named with domains; (3) baseline figures incorporated (distress 20.4%, anxiety 37.2%, depression 26.2%); (4) age range 16–80 years added; (5) conclusion fully revised in this revision with cautious hedged language and explicit design limitation acknowledgement.

Title

Previous Reviewer Concern: Title may benefit from restructuring to better reflect primary aim.

Response/Action: Revised to: 'Changes in Psychosocial Wellbeing Among Migrant Workers and their Families in Nepal: Evidence from a Longitudinal Evaluation of the PARBARDHAN Community-Based Intervention.' Implemented in first revision, confirmed.

Introduction — PARBARDHAN description, district rationale, baseline figures, citations

Previous Reviewer Concern: More detail on PARBARDHAN needed. District selection rationale needed. Baseline/midline figures needed. Additional literature needed.

Response/Action: All addressed: PARBARDHAN project description expanded with naming clarification (implemented in this revision), project period, partners, coverage, key interventions. District rationale added (among highest out-migration districts). Baseline figures stated. Additional literature incorporated. Citations standardised.

Methods — Design statement, GRAMMS, sampling, eligibility, tools

Previous Reviewer Concern: Opening statement should be restructured. GRAMMS alignment encouraged. Greater sampling detail needed. Inclusion criteria, age eligibility, tool suitability needed.

Response/Action: All addressed in first revision: design statement restructured; GRAMMS guideline cited; quantitative and qualitative sampling procedures fully described; Cochran formula stated; eligibility criteria added; saturation per Saunders et al. (2018); tool validation noted; framework analysis specified with five stages.

Results — Female dominance, minor participant, subgroup, tables, statistics

Previous Reviewer Concern: Female dominance unexplained. One participant below 18. Subgroup analysis needed. Table 5 midline N missing. Statistical tests needed.

Response/Action: All addressed: female dominance explained; 17-year-old participant with physical disability identified and justified; subgroup limitation acknowledged; three-phase comparison table (Table 4a) added in this revision; midline N added to Table 5 in this revision; methodological inappropriateness of formal inferential tests explained.

Discussion — Strengthening

Previous Reviewer Concern: Thematic interpretation needs clearer structure. Greater international comparison needed. Insufficiently supported claims. Stronger scientific framing needed.

Response/Action: Discussion substantially expanded in first revision with: thematic structure; comparative evidence (Hoeft et al., Jordans et al., Luitel et al.); evidence-based explanation of divergent GHQ-12/HSCL-25 findings; contextualisation of life satisfaction decline; theoretical framing; stronger critical interpretation throughout.

Conclusion — Cautious framing

Previous Reviewer Concern: Conclusions appear somewhat overstated relative to analyses presented.

Response/Action: Conclusion fully revised in this revision. Causal language replaced with hedged framing. Cross-sectional design limitation, contrasting findings, and predominantly female sample explicitly acknowledged. Recommendations grounded in actual findings.

Minor Concerns — Grammar, spelling, flow, referencing

Previous Reviewer Concern: Syntax and grammatical inconsistencies, spelling errors, flow issues, citation formatting.

Response/Action: Full proofread completed in this revision. Additional typographical errors identified and corrected (see Additional Corrections 1–6 above). References standardised with journal titles throughout.

**REVIEWER 2 COMMENTS**

Abstract — Analyses description and actual changes

Previous Reviewer Concern: Add description of analyses done, not just software. 'These outcomes reflect changes' does not describe actual changes.

Response/Action: Addressed in this revision: abstract now states — 'Quantitative data were analysed using descriptive statistics and bivariate analyses, with retrospective comparison to baseline and midline findings. Qualitative data were analysed using framework analysis.' Explicit figures now given: anxiety 37.2% to 8.2%, depression 26.2% to 5.3%, distress increased 20.4% to 34.7%.

Introduction — Return migration figures, EMHPW-MW/PARBARDHAN, project description, baseline indicators

Previous Reviewer Concern: How many Nepalis return annually? EMHPW-MW and PARBARDHAN relationship unclear. Describe what PARBARDHAN actually did. No baseline indicators described.

Response/Action: All addressed: (1) contextual statement on reintegration challenges added instead of unsupported figure; (2) naming relationship explicitly clarified in this revision — PARBARDHAN is the Nepali community name for the project formally titled EMHPW-MW in English; (3) intervention components described; (4) baseline figures stated with instruments.

Methods — Multiple clarifications

Previous Reviewer Concern: Sampling, comparability, saturation, tool validation, FGD guide, venue, data location, statistical analysis, baseline comparison, thematic analysis type.

Response/Action: All addressed in first revision: random sampling described; independent cross-sectional samples noted; saturation per Saunders et al. (2018); tool validation stated; semi-structured FGD guide described with topics; KII and FGD venues stated; household data collection stated; normally distributed scores; means ± SD reported; three-phase comparison table added in this revision; framework analysis specified.

Results — Female dominance, tables, baseline presentation, qualitative themes, quotes, KII/IDI error

Previous Reviewer Concern: Nearly all participants female. Tables need more information. Baseline results not presented. Themes not listed. Themes underdeveloped. Quote attribution error.

Response/Action: All addressed: female dominance explained; three-phase comparison table (Table 4a) added in this revision; midline N added to Table 5; eight themes listed at opening of Section 3.2; thematic sections strengthened with analytical interpretation; IDI/KII attribution corrected (confirmed as IDI, Ghodaghodi).

Discussion — Strengths and limitations

Previous Reviewer Concern: Please add a paragraph on strengths and limitations.

Response/Action: Dedicated strengths and limitations section added in first revision and confirmed: strengths (mixed-methods triangulation, validated instruments, multi-phase data, inclusive sampling); limitations (independent cross-sectional samples, no control group, predominantly female sample, no subgroup disaggregation, missing baseline data for two instruments, social desirability bias risk).

References and proofreading

Previous Reviewer Concern: Most references lack journal titles. Multiple typos and grammatical errors.

Response/Action: Journal titles added to all references in first revision. Additional typographical corrections made in this revision as detailed in Additional Corrections 1–6 above.

REVIEWER 3 COMMENTS

Critical Concern 1 — Data collection date February 2026

Previous Reviewer Concern: Manuscript reports data collected in February 2026 — serious anachronism.

Response/Action: Typographical error — corrected to February 2025 in first revision and confirmed.

Critical Concern 2 — Contradictory outcomes

Previous Reviewer Concern: Distress increased 20.4% to 34.7% yet framed as effectiveness. Life satisfaction collapsed without demonstrating economic factors changed.

Response/Action: Fully addressed in first revision: two evidence-based mechanisms presented for distress divergence; life satisfaction decline contextualised with explicit acknowledgement of absent concurrent economic data and compositional differences between independent samples; causal attribution not claimed. Confirmed in this revision.

Critical Concern 3 — Female sample and migrant worker claims

Previous Reviewer Concern: 94% female sample severely limits claims about migrant workers themselves.

Response/Action: Major limitation explicitly stated throughout Results, Discussion, Limitations, and revised Conclusion in this revision. Findings qualified as primarily representing left-behind family members.

Minor Concerns — Transparent reporting and Table 5

Previous Reviewer Concern: Report distress increase transparently. Table 5 missing midline N.

Response/Action: Distress increase now reported explicitly in abstract and results. Midline N added to Table 5 in this revision.

REVIEWER 4 COMMENTS

Comment 1 — Punctuation and grammar

Previous Reviewer Concern: Greater attention to punctuation and grammar for effective communication.

Response/Action: Full proofread completed. Additional corrections made in this revision: 'formally totled' corrected; incomplete sentences fixed; 'society/country' replaced; 'during the experiment' corrected; informal phrasing standardised. See Additional Corrections section above.

Comment 2 — Sample size formula

Previous Reviewer Concern: Clearly indicate the formula used for sample size determination.

Response/Action: Formula explicitly stated in Methods: n = Z²pq/d², where Z=1.96, p=13.2%, q=86.8%, d=0.05, yielding minimum 177; with 20% non-response rate, estimated 212; 245 enrolled. Implemented in first revision, confirmed.

Comment 3 — Standard deviation notation

Previous Reviewer Concern: Standard deviation should use ± notation.

Response/Action: ± notation applied throughout. Mean age reported as 38.16 ± 12.12 years. Implemented in first revision, confirmed.

We believe these corrections, together with those made in the first revision, have now fully addressed all concerns raised by the reviewers and Editor. We sincerely apologise for the inconsistencies in the first revision and are grateful to the Editor for the opportunity to correct them. We hope the manuscript now meets the publication standards of PLOS Global Public Health.

Sincerely,

Himal Gaire and Co-authors

Centre for Mental Health and Counselling Nepal (CMC-Nepal)

Corresponding author: Himal.gaire@cmcnepal.org

Manuscript ID: PGPH-D-26-01439R1
