# Supplementary material for: Mental health and psychosocial wellbeing among beneficiaries of a community-based intervention in Nepal: Findings from a cross-sectional endline evaluation of the PARBARDHAN project
Source: PLOS Glob Public Health. 2026 Oct 16;6(10):e0006666. doi: 10.1371/journal.pgph.0006666 (PMC13674775; doi:10.1371/journal.pgph.0006666)
Supplement: Supplementary file 7 [file pgph.0006666.s007.docx]

Response to Editor

Manuscript ID: PGPH-D-26-01439R2

Title: Changes in Psychosocial Wellbeing Among Families and Communities of Migrant Workers in Nepal: Evidence from an Endline Evaluation of the PARBARDHAN Community-Based Intervention

Journal: PLOS Global Public Health

Date: 01 August 2026

Dear Dr. Hossain,

Thank you for the opportunity to revise our manuscript (PGPH-D-26-01439R2). We are grateful for your assessment that the manuscript addresses a critical issue and provides adequate data and insight, and we have carefully addressed both of the conceptual points you raised. All changes are visible in the Revised Manuscript with Track Changes file. Our point-by-point response is below.

**Comment 1 — Title and study population**

Reviewer/Editor comment: Although the title has been revised, it appears to place greater emphasis on migrant workers themselves. However, the study sample consists predominantly of family members rather than migrant workers, which may be somewhat misleading, particularly in the title. This is particularly important because the analysis does not present the data separately for these two groups.

Response: We agree, and thank the Editor for this important observation. On review of Table 2, we confirmed that 88.2% of respondents reported having a family member currently working abroad (husband 51.4%, son/daughter 29.7%, parent 0.8%, other 6.1%), while only 11.8% reported no family member abroad. This confirms that the large majority of respondents are left-behind family members of migrant workers rather than migrant workers themselves, and the occupational profile of the sample (predominantly housewives and agricultural workers) is consistent with this.

We have made the following changes:

1. The title has been revised to: “Changes in Psychosocial Wellbeing Among Families and Communities of Migrant Workers in Nepal: Evidence from an Endline Evaluation of the PARBARDHAN Community-Based Intervention.” This foregrounds families and communities rather than migrant workers themselves, and more accurately reflects the composition of the study sample.
2. The short title has been revised to “Mental Health of Migrant Worker Families in Nepal.”
3. We have added an explicit statement in the Results (Section 3.1.1) reporting the 88.2% figure and its implication for interpreting the sample.
4. We have expanded the Limitations paragraph to state plainly that the sample was overwhelmingly composed of left-behind family members rather than migrant workers themselves, that the study did not formally disaggregate the two groups in subgroup analyses, and that findings should be interpreted primarily as evidence about migrant-worker households and communities rather than about migrant workers directly. We note this as a direction for future research, since disaggregated subgroup analysis was not feasible within the current dataset and sample composition.

**Comment 2 — Interpretation of changes over time**

Reviewer/Editor comment: The authors appropriately acknowledge the limitation of having a different population at endline due to the repeated cross-sectional study design. However, this limitation also affects the interpretation of changes observed between baseline, midline, and endline, since the endline participants are not the same individuals assessed previously. It may be more appropriate to frame the manuscript primarily as an endline evaluation, using baseline and midline findings for contextual comparison rather than implying longitudinal improvement.

Response: We agree with this assessment and thank the Editor for the specific example provided, which we have followed closely in reframing the manuscript. We have revised language throughout the Title, Abstract, Introduction, Methods, Results, Discussion, and Conclusion that previously implied within-person or within-cohort change (e.g., “improved,” “declined,” “sustained gain,” “building on the improvements observed at midline”) to instead describe differences between evaluation phases, consistent with the repeated cross-sectional design. Specific changes include:

1. The title now reads “Evidence from an Endline Evaluation” rather than “Evidence from a Longitudinal Evaluation,” to correctly frame the study design.
2. In the Abstract, “By midline, significant improvements were recorded in anxiety and depression levels… remained substantially reduced compared to baseline” has been revised to state that the midline evaluation, conducted with a different sample, reported lower prevalence, and that endline prevalence was lower than at baseline — without implying change within the same individuals. The concluding sentence has been similarly revised to state that the endline evaluation found lower prevalence and stronger stigma/support outcomes than earlier evaluation phases, rather than describing these as “sustained gains” built on midline improvement.
3. In the Introduction, the summary of the midline evaluation and the transition into the endline evaluation have been reworded to avoid implying a single longitudinal trajectory across phases.
4. In the Methods, “assess the trajectory of change over the project period” has been revised to “assess patterns of difference across evaluation phases,” consistent with the independent cross-sectional design already described elsewhere in the Methods.
5. In the Results (Section 3.1.4), the description of psychological distress, anxiety, and depression prevalence across baseline, midline, and endline has been reworded from directional change language (“increased,” “declined substantially,” “worsening… improving”) to comparative language describing prevalence differences between phases.
6. In the Discussion, the paragraph describing anxiety and depression reduction as an “achievement… decreased substantially over the project period” has been reworded to describe lower prevalence at endline relative to earlier phases. The sentence attributing “gains… sustained through to project completion” to programmatic durability has been revised to explicitly note that, because each phase drew an independent sample, the pattern cannot be interpreted as change sustained within the same individuals. The sentence describing “a substantial decline in life satisfaction… over the project period” has been revised to state that life satisfaction was lower at endline than at midline, without implying within-population decline (this sits alongside the existing caveat, retained from the prior revision, that the midline and endline samples were independent and not the same participants).
7. In the Conclusion, the opening sentence has been revised to state that the endline evaluation found lower prevalence of anxiety and depression, and increased awareness, stigma reduction, and service availability, relative to earlier evaluation phases, with an explicit statement that these differences should be interpreted as contrasts between evaluation points rather than change experienced by the same individuals.

We believe these revisions, together with those addressing the title and sample composition, more accurately reflect the study’s repeated cross-sectional endline-evaluation design and address both of the Editor’s remaining conceptual concerns. We thank the Editor again for the constructive and specific guidance, and we hope the revised manuscript is now suitable for publication.

Sincerely,

Himal Gaire and Co-authors

Centre for Mental Health and Counselling Nepal (CMC-Nepal)

Corresponding author: Himal.gaire@cmcnepal.org
