# Supplementary material for: Mental health and psychosocial wellbeing among beneficiaries of a community-based intervention in Nepal: Findings from a cross-sectional endline evaluation of the PARBARDHAN project
Source: PLOS Glob Public Health. 2026 Oct 16;6(10):e0006666. doi: 10.1371/journal.pgph.0006666 (PMC13674775; doi:10.1371/journal.pgph.0006666)
Supplement: Supplementary file 8 [file pgph.0006666.s008.docx]

**Response to the reviewer 7**

**Reviewer Comment 1: Title**

**Response:**
Thank you for this important observation. We agree that the previous title did not adequately reflect the heterogeneity of the endline study population or clearly communicate the cross-sectional nature of the evaluation. The endline sample included beneficiaries from several groups associated with the PARBARDHAN program, rather than exclusively migrant workers and their left-behind family members. We also agree that the phrase “Insights from” was nonspecific.

We have therefore revised the title to more accurately describe the study population, program context, and study design. We have also avoided terminology that could imply longitudinal follow-up of the same individuals across the baseline, midline, and endline evaluation rounds.

**Revised title:**
“Mental Health and Psychosocial Wellbeing Among Beneficiaries of a Community-Based Intervention in Nepal: Findings from a Cross-Sectional Endline Evaluation of the PARBARDHAN Program.”

This revision also aligns the title with our clarification in the Methods, Results, Discussion, and Limitations that the evaluation rounds comprised independent cross-sectional samples and therefore do not permit inference about within-person longitudinal change or causal intervention effects.

**Reviewer Comment 2.1: Use of Causal Language**

**Response:**
Thank you for this important observation. We agree that the previous wording in the Abstract was stronger than warranted by the study design. In particular, statements referring to “changes observed following the implementation” and concluding that the PARBARDHAN project “effectively improved” psychosocial wellbeing could be interpreted as causal claims. Because the baseline, midline, and endline evaluations were based on independent cross-sectional samples rather than longitudinal follow-up of the same individuals, such causal interpretations are not supported.

We have therefore revised the Abstract throughout to ensure that the findings are presented as descriptive differences observed across independent evaluation rounds. We have removed language implying that the observed differences were caused by the PARBARDHAN intervention. We now explicitly state that the baseline, midline, and endline findings were compared descriptively and that these comparisons do not represent longitudinal follow-up or establish causal intervention effects.

The revised Abstract reports the observed differences in probable psychological distress, anxiety, depression, life satisfaction, and happiness without attributing these differences directly to the intervention. We have also applied the same principle throughout the Results, Discussion, and Conclusion to ensure consistency in interpretation.

**Reviewer Comment 2.2: Statement Regarding Suicide Prevention**

**Response:**
We agree with the reviewer that the previous wording regarding reduced suicide risk and prevention of suicide attempts was not supported by a systematic population-level assessment of suicide risk. The qualitative component captured participants’ experiences and perceptions of support during periods of severe emotional distress, but these accounts cannot be used to estimate a population-level reduction in suicide risk or establish that the intervention prevented suicide attempts.

We have therefore removed the statement indicating that the PARBARDHAN project reduced suicide risk from the Abstract. We have also revised the relevant qualitative findings and their interpretation throughout the manuscript so that they are presented as participants’ reported experiences of emotional and social support rather than evidence of a population-level reduction in suicide risk or a causal prevention effect.

The revised Abstract now states that participants described counselling, peer support, and community activities as helpful sources of support during periods of severe emotional distress. This wording reflects the qualitative evidence while avoiding unsupported causal or population-level claims.

**Reviewer Comment 3: Introduction**

**Response:**
Thank you for this detailed and constructive comment. We agree that the previous version of the Introduction did not sufficiently develop the conceptual relationship between labor migration, psychosocial determinants, mental health and wellbeing outcomes, access to services, and community-based MHPSS. We also agree that the scientific rationale and knowledge gap for the endline evaluation required clearer articulation.

We have substantially reconstructed the Introduction rather than simply adding additional references. The revised Introduction now follows a clearer conceptual sequence from the context of labor migration to the mental health burden among migrant workers, the psychosocial consequences for families remaining in the country of origin, migration-related social and structural determinants, barriers to mental health services, evidence on community-based MHPSS approaches, the Nepal-specific context and PARBARDHAN program, and finally the remaining knowledge gap and scientific rationale for the endline evaluation.

**3.1. Insufficiently Developed Conceptual Framework**

We have expanded the Introduction to establish a clearer conceptual framework linking labor migration with mental health and psychosocial wellbeing. The revised text discusses migration-related determinants including family separation, economic insecurity, employment uncertainty, discrimination, exploitation, social isolation, limited social support, stigma, and barriers to healthcare. These factors are then linked conceptually with the outcomes assessed in the study, including psychological distress, anxiety, depression, life satisfaction, and happiness.

We have also clarified the potential protective role of social and community resources, including family relationships, peer support, self-help groups, community participation, counselling, mental health awareness, and integration of mental health services into primary healthcare. This provides a clearer rationale for examining both individual mental health outcomes and the qualitative dimensions of support, stigma, service access, inclusion, and sustainability.

**3.2. Insufficient Use of Systematic Reviews and Meta-analyses**

We have substantially updated the literature underpinning the Introduction and incorporated relevant systematic reviews and meta-analyses to establish the current state of evidence rather than adding references solely to increase the number of citations.

The revised Introduction now incorporates evidence concerning the prevalence and determinants of common mental health problems among migrant workers, the psychosocial wellbeing of left-behind family members, and evidence concerning community-based approaches to mental health and psychosocial support. We have also incorporated evidence showing that findings among migration-affected populations can vary across settings and family characteristics.

In particular, the revised Introduction uses systematic evidence to demonstrate the substantial burden of anxiety and depression among migrant workers and the importance of occupational, environmental, individual, and social determinants. It also acknowledges the heterogeneous evidence concerning left-behind family members and avoids presenting migration as having uniformly negative effects across all populations and settings.

We have therefore used the literature to establish both what is known and what remains uncertain, and to provide a stronger scientific basis for interpreting the findings of the present study.

**3.3. Insufficiently Defined Knowledge Gap**

We agree with the reviewer that the previous Introduction did not sufficiently distinguish the scientific question addressed by the endline evaluation from the fact that the project had reached its endline.

We have therefore explicitly articulated the remaining knowledge gaps after the baseline and midline evaluations. The revised Introduction explains that the baseline established the burden of common mental health problems and contextual barriers, while the midline reported lower proportions screening positive for anxiety and depression and higher reported happiness and life satisfaction, together with expansion of mental health services. However, these evaluation rounds comprised independent cross-sectional samples and therefore did not establish individual-level trajectories or causal intervention effects.

The revised Introduction now identifies the specific questions addressed by the endline evaluation: (1) the mental health and psychosocial wellbeing profile of participants at the end of the project; (2) the descriptive differences between endline and earlier evaluation rounds across multiple dimensions of wellbeing; (3) whether different mental health and wellbeing indicators showed consistent or discordant patterns; and (4) how qualitative experiences related to social support, stigma, service access, inclusion, and sustainability help contextualize the quantitative findings.

We have also explicitly identified the primary quantitative outcomes as psychological distress, anxiety, depression, life satisfaction, and happiness. The qualitative component is described as providing contextual information on mental health awareness, social and community support, stigma, service access, inclusion, and sustainability.

Finally, we have clarified that the baseline, midline, and endline samples were independent cross-sectional samples. Accordingly, the revised Introduction states that comparisons across evaluation rounds represent differences between populations assessed at different evaluation points rather than within-person longitudinal change, and that the study was not designed to estimate the causal effectiveness of PARBARDHAN. This clarification has also been applied consistently to the Abstract, Methods, Results, Discussion, Limitations, and Conclusion.

**Reviewer Comment 4.1: Study Design and Attribution of Effectiveness**

**Reviewer comment:**
The manuscript describes the study as cross-sectional and uses the expression “retrospective elements”; however, it is not sufficiently clear what information was obtained retrospectively, what time period this information covers, and how this characteristic relates to the overall study design. The reviewer requested clarification of the epidemiological design, the meaning of “retrospective elements,” the information corresponding to each evaluation round, whether participants were individually followed, the distinction between descriptive comparisons and causal evaluation, and removal of unsupported causal language.

**Response:**
Thank you for this important methodological clarification. We agree that the previous use of the terms “retrospective elements” and “retrospective comparison” was insufficiently precise and could imply that retrospective participant-level information had been collected during the endline evaluation.

We have substantially revised Section 2.2, “Study design and attribution of effectiveness,” to clarify the study design and the interpretation of comparisons across evaluation rounds. The endline evaluation is now explicitly described as a cross-sectional mixed-methods evaluation. The quantitative component consisted of a cross-sectional survey conducted during the endline fieldwork period from 3 to 26 February 2025, while the qualitative component consisted of FGDs and KIIs examining project experiences, implementation, perceived benefits, service access, stigma, coping, social support, and sustainability.

We have also clarified that the term “retrospective comparison” did not refer to retrospective collection of participants’ previous mental health information. Rather, it referred only to the use of previously completed baseline and midline evaluation findings for descriptive comparison with the newly collected endline findings. No participant was asked to recall their baseline or midline mental health status or scores.

Importantly, we have explicitly stated that the baseline, midline, and endline evaluations used independent cross-sectional samples. Participants were not individually followed across evaluation rounds, and no longitudinal participant-level dataset was constructed. The study therefore did not involve random allocation, a control group, a concurrent comparator group, individual follow-up, or a quasi-experimental design.

We have consequently clarified that comparisons between evaluation rounds are descriptive comparisons of observed proportions and patterns at different evaluation points, rather than estimates of within-person change or causal intervention effects. The manuscript now explicitly states that observed differences across baseline, midline, and endline cannot be causally attributed to the PARBARDHAN intervention.

In addition, we have revised the interpretation throughout the manuscript to distinguish participants perceived benefits and observed differences in outcomes from evidence of intervention effectiveness. Causal terminology that could not be supported by the study design has been removed or replaced with appropriately cautious language.

These revisions are intended to ensure that the methodological design, analytical approach, and interpretation of findings are fully aligned and that the manuscript does not imply a level of causal inference that the study design cannot support.

**Reviewer Comment 4.2: Comparability of Baseline, Midline, and Endline**

**Reviewer comment:**
The reviewer raised concerns regarding the comparability of the baseline, midline, and endline samples, noting that the samples were independent and that observed differences could reflect differences in sample composition, sociodemographic characteristics, migration status, socioeconomic conditions, selection procedures, temporal factors, or measurement procedures. The reviewer requested a detailed comparison of the three samples and clarification regarding the sampling frame and selection procedures.

**Response:**
Thank you for this important methodological comment. We agree that, because the baseline, midline, and endline assessments used independent cross-sectional samples, differences in observed outcomes cannot be interpreted as individual-level changes without accounting for differences in sample composition.

We have revised the manuscript to make this limitation explicit. The three evaluation rounds were conducted within the PARBARDHAN project areas in Salyan and Kailali and targeted the project's beneficiary population, but participants were not individually followed across assessment rounds. Thus, the samples should be considered independent cross-sectional samples rather than a longitudinal cohort.

We have also clarified that the endline sampling procedure involved selection from a comprehensive beneficiary list obtained from CMC-Nepal for each municipality, using a random number generator, with replacement of individuals who were unavailable or did not consent. We have avoided stating that the sampling procedures were identical across all three evaluation rounds because sufficiently detailed and harmonized documentation of the baseline and midline sampling procedures was not available to the authors for direct verification.

We acknowledge that detailed participant-level characteristics from the baseline and midline assessments were not available in a sufficiently harmonized format to permit a complete variable-by-variable comparison of age, sex, migration status, beneficiary category, socioeconomic characteristics, and disability status across all three rounds. We therefore have not imputed or reconstructed values that cannot be verified from the available evaluation documentation. Instead, the revised manuscript explicitly acknowledges that differences in sample composition may have contributed to the observed differences between evaluation points.

The endline sample was predominantly female (94.3%) and largely represented left-behind family members, with 88.2% of respondents reporting a family member currently working abroad. We have added greater emphasis to this sample composition in the Methods, Results, Discussion, and Limitations sections because it is particularly relevant to interpretation and generalisability.

We have also revised the interpretation of the cross-round findings. The baseline, midline, and endline estimates are now described as descriptive differences between independent cross-sectional samples, rather than changes occurring within the same individuals. We explicitly acknowledge that these differences may reflect sample composition, sociodemographic and migration characteristics, geographical and socioeconomic differences, temporal changes, selection factors, and differences in measurement or data-collection procedures. Consequently, the manuscript does not interpret differences between the three rounds as evidence of a causal effect of the PARBARDHAN intervention.

These revisions ensure that the conclusions drawn from the available data remain consistent with the cross-sectional, non-randomised design and avoid attributing observed differences to the intervention without appropriate causal evidence.

**Reviewer Comment 4.2: Comparability of Baseline, Midline, and Endline**

*This is probably the most important methodological problem. The manuscript compares different phases but acknowledges that the samples were independent. Observed differences may therefore be due to the intervention, but also to sample composition, sociodemographic differences, territorial differences, migration status, economic changes, selection differences, and instrument/procedure differences. This is particularly important because the endline sample consists of 231 women (94.3%) and 14 men (5.7%). Please provide a detailed comparison of baseline, midline, and endline samples, including sample size, age, sex, district, migration status, migrant worker/family member status, socioeconomic variables, disability, and other characteristics. Please explain whether the same sampling frame was used and whether selection procedures were comparable. The Discussion should also acknowledge that independent samples cannot be directly interpreted as individual changes over time.*

**Response:**

Thank you for this important methodological comment. We agree that, because the baseline, midline, and endline assessments used independent cross-sectional samples, differences in observed outcomes cannot be interpreted as individual-level changes over time. We have therefore revised the manuscript to make this issue more explicit and to ensure that comparisons across the three evaluation phases are interpreted appropriately.

The three evaluation rounds were conducted within the PARBARDHAN project areas in Salyan and Kailali and targeted the project's beneficiary population; however, participants were not individually followed across the assessment rounds. Thus, the three rounds should be considered independent cross-sectional assessments rather than a longitudinal cohort.

We have added a new subsection in the Methods, **Section 2.4, “Comparability of baseline, midline, and endline samples,”** to clarify the design and limitations of cross-round comparisons. We have also added **Table 2A** to summarize the available information regarding the study populations, assessment design, geographical coverage, sample selection, and comparability across the three evaluation rounds.

For the endline assessment, a comprehensive beneficiary list was obtained from CMC-Nepal for each municipality, and participants were selected using a random number generator, with replacement of individuals who were unavailable or did not provide consent. We have described this procedure more clearly in the revised Methods section.

We acknowledge that detailed participant-level demographic information from the baseline and midline assessments was not available to the authors in a sufficiently harmonized format to permit a complete variable-by-variable comparison of age, sex, migration status, beneficiary category, socioeconomic characteristics, and disability status across all three rounds. We have therefore not reconstructed, estimated, or imputed values that could not be verified from the available evaluation documentation. Instead, the revised manuscript explicitly acknowledges this limitation and avoids presenting the samples as fully comparable.

We have given particular attention to the composition of the endline sample. Of the 245 endline participants, 94.3% were women and 5.7% were men. In addition, 88.2% reported having a family member currently working abroad. This composition indicates that the endline findings predominantly represent the experiences of women and left-behind family members rather than migrant workers themselves. We have clarified this issue in the Results, Discussion, and Limitations sections and have considered its implications for interpretation and generalisability.

We have also revised the interpretation of differences in mental health indicators across baseline, midline, and endline. These estimates are now explicitly presented as **descriptive differences between independent cross-sectional samples at different evaluation points**, rather than changes experienced by the same individuals. We acknowledge that differences may reflect variation in sample composition, age, sex, migration status, beneficiary category, district or municipality, socioeconomic circumstances, disability status, participant selection, temporal context, and differences in measurement or data-collection procedures.

Accordingly, we have removed or softened language that could imply that observed differences necessarily represent individual-level improvement or a causal effect of the PARBARDHAN intervention. The Discussion and Limitations now explicitly state that the independent cross-sectional design does not permit estimation of individual-level change or attribution of observed differences to the intervention.

We believe these revisions provide a more cautious and methodologically appropriate interpretation of the findings and directly address the concern regarding comparability of the three assessment samples.

**Reviewer Comment 4.3: Population Composition**

**Reviewer comment:**
“There is an important discrepancy between the population suggested by the title and the population actually analyzed. A considerable proportion of the sample does not clearly correspond to migrant workers. Requested Action: authors should clearly present number and proportion of participants belonging to each population category and explain: why included; what analyses conducted for each group; whether all participants exposed to intervention; whether outcomes analyzed jointly or by subgroups. If conceptually different populations combined, epidemiologically justify.”

**Response:**

Thank you for identifying this important issue. We agree that the original manuscript did not adequately distinguish between the broader PARBARDHAN evaluation population and the population represented in the quantitative endline analysis. We have therefore substantially revised the description of the study population throughout the manuscript.

The PARBARDHAN project was designed to address the mental health and psychosocial wellbeing of migrant workers and their families, with specific attention to persons with mental health conditions and persons with disabilities. The broader evaluation also involved SHG and peer-support group members, health workers, local government representatives, and other community stakeholders. The project documentation identifies migrant workers, families of migrant workers, persons with mental health conditions, and persons with disabilities as its primary beneficiary groups.

**Population composition of the quantitative sample**

We re-examined the endline quantitative dataset and have now explicitly presented the population composition in a new table. Among the 245 quantitative respondents, 20 (8.2%) were classified as migrant/returnee workers and 225 (91.8%) as family/community respondents. In addition, 216 respondents (88.2%) reported having a family member currently abroad, while 29 (11.8%) reported no family member currently abroad.

Among those reporting a family member abroad, the person abroad was most commonly a husband (126, 51.4%) or son/daughter (73, 29.7%), followed by father/mother (2, 0.8%) and other relatives (15, 6.1%). These findings demonstrate that the quantitative endline sample predominantly represents family and community members associated with migrant-affected households rather than migrant workers themselves.

We have therefore revised the Abstract, Methods, Results, Discussion, and Limitations to avoid implying that the quantitative findings are primarily estimates among migrant workers.

**Why were these populations included?**

The inclusion of family and community respondents was conceptually consistent with the PARBARDHAN intervention. The project addressed the psychosocial consequences of labor migration at individual, household, and community levels. Its stated goal was to improve mental health and psychosocial wellbeing among migrant workers and their families, while also strengthening disability inclusion and community-based mental health systems.

Family members were therefore not considered an unrelated comparison population. They were a central target population of the intervention because family separation, migration-related uncertainty, economic stress, social support, and stigma can affect both migrants and households remaining in the community.

**Persons with disabilities and persons with mental health problems**

We also clarified that disability and mental health status are overlapping characteristics rather than mutually exclusive population categories. In the quantitative dataset, 74 respondents (30.2%) were coded as having a disability and 68 (27.8%) reported a mental health problem. These respondents could simultaneously belong to the migrant/returnee or family/community categories and therefore were not added to those groups.

The project explicitly incorporated disability inclusion into its community-based approach, including engagement with persons with disabilities, self-help groups, and organizations of persons with disabilities.

**Were all participants exposed to the intervention?**

Participants were recruited from the project beneficiary population and project communities. However, we do not assume that every respondent received the same intervention component or intensity.

PARBARDHAN consisted of multiple activities, including mental health awareness, psychosocial counselling, self-help and peer-support groups, referral mechanisms, health-worker capacity building, disability inclusion, livelihood-related activities, and engagement with local governments and health institutions. Consequently, exposure was heterogeneous across respondents.

We have revised the Methods section to make this explicit and have removed wording that could imply a uniform intervention dose across all participants.

**How were outcomes analyzed?**

The quantitative mental health and psychosocial wellbeing outcomes were analyzed jointly for the 245 respondents because the study was designed to provide an overall assessment of psychosocial wellbeing among the endline beneficiary population.

We did not combine the qualitative participants with the quantitative sample. Qualitative data from SHG and peer-support group members, health workers, local government representatives, and other stakeholders were analyzed separately using framework analysis and were used to contextualize the quantitative findings.

We also did not conduct separate inferential outcome analyses for every population category. The migrant/returnee subgroup consisted of only 20 respondents, and the study was not designed or powered to provide robust subgroup-specific estimates across all beneficiary categories. We therefore consider the overall quantitative analysis to be the most appropriate primary analysis while presenting the population composition transparently.

**Epidemiological justification for combining the populations**

The populations were combined at the level of the overall evaluation because they represented interconnected groups within the same migration-affected community and were exposed to a community-based intervention operating across individual, household, community, and health-system levels.

However, we agree that conceptual relevance does not mean that these populations are epidemiologically interchangeable. We have therefore revised the manuscript to distinguish clearly between the primary respondent categories and overlapping characteristics.

Most importantly, we now explicitly state that the quantitative findings predominantly represent family/community respondents and should not be interpreted as direct estimates of mental health outcomes among migrant workers themselves.

**Changes made in the manuscript**

In response to this comment, we have:

1. Added a new table presenting the population composition of the 245 quantitative respondents.
2. Distinguished migrant/returnee workers from family/community respondents.
3. Reported the number and proportion of respondents with a family member currently abroad.
4. Clarified that disability and reported mental health problems are overlapping characteristics rather than mutually exclusive populations.
5. Revised the Methods section to distinguish the broader evaluation population from the quantitative analytic population.
6. Revised the Results section to explicitly describe the predominance of family/community respondents.
7. Revised the Discussion to interpret the findings primarily in relation to migrant-affected families and communities.
8. Revised the Limitations to acknowledge the limited representation of migrant/returnee workers and the resulting limitation in generalizability.
9. Clarified that intervention exposure was heterogeneous rather than uniform.
10. Clarified that quantitative outcomes were analyzed jointly because the study was not designed or powered for comprehensive subgroup-specific inference.

We believe these revisions provide a more transparent and epidemiologically appropriate description of the population represented by the endline quantitative analysis.

**Sample size**

**Quantitative data**

The quantitative sample size was calculated using the Cochran formula for estimating a single population proportion:

$$n=\frac{Z^{2}pq}{d^{2}}$$

where *n* is the required sample size, *Z* is the standard normal value corresponding to the desired confidence level, *p* is the anticipated prevalence, *q* = 1 − *p*, and *d* is the desired margin of error. The prevalence of depression was selected as the basis for the sample-size calculation because depression was one of the key mental health outcomes assessed in the evaluation. The assumed prevalence (*p*) was 13.2%, based on the prevalence of depression observed among project beneficiaries during the midterm evaluation.

Using a 95% confidence level (*Z* = 1.96), 5% absolute precision (*d* = 0.05), and an assumed prevalence of 13.2% (*p* = 0.132), the calculated minimum sample size was 177 participants. An additional 20% was added to account for potential non-response, resulting in a target sample size of 212 participants. Simple random sampling was used to select participants from the available beneficiary lists. A total of 245 participants were ultimately included in the endline quantitative assessment, exceeding the calculated target sample size.

The sample-size calculation was primarily intended to provide adequate precision for estimating the prevalence of the prespecified mental health outcome, depression. Other outcomes assessed in the evaluation, including anxiety, psychological distress, and psychosocial wellbeing indicators, were considered additional outcomes and were not individually used to determine the sample size. Therefore, the achieved sample size should not be interpreted as having been separately powered to estimate each outcome with the same level of precision.

The evaluation used independent cross-sectional samples at the different assessment phases. Thus, the quantitative sample size was not calculated to provide statistical power for paired longitudinal comparisons of the same participants across baseline, midline, and endline. The assessment was primarily designed to estimate and describe the prevalence and distribution of mental health and psychosocial outcomes at each evaluation phase. Any comparison across phases therefore represents differences between independent cross-sectional samples rather than individual-level changes over time.

**Qualitative data**

The qualitative sample size was determined using a purposive sampling approach guided by data saturation and the qualitative objectives of the evaluation. The qualitative component was designed to obtain in-depth perspectives from relevant beneficiary and stakeholder groups and to provide contextual information regarding experiences, perceived changes, implementation processes, barriers, facilitators, and community-level responses to the intervention. It was not intended to generate statistically representative prevalence estimates.

The qualitative component comprised seven focus group discussions (FGDs) and 14 key informant interviews (KIIs). Four FGDs were conducted with self-help group (SHG) members across Ghodaghodi, Tikapur, Janaki, and Chhatreshwori, and three FGDs were conducted with peer support group members across Sharada, Bangadkupinde, and Bagchaur. Each FGD was planned to include approximately eight participants.

The 14 KIIs included five mayors/chairpersons, two chief administrative officers, five health section chiefs, and two health facility in-charges from the participating municipalities. The interviews used structured or semi-structured guides appropriate to the respondent category. The qualitative sample was considered adequate based on the planned coverage of relevant stakeholder and beneficiary groups and the attainment of sufficient thematic information to address the qualitative objectives.

**Reviewer 4.5: Instruments and Measurement Validity**

**Reviewer comment:**
GHQ-12, HSCL-25, and life satisfaction and happiness tools are used. However, the methodological description is not sufficiently complete to assess the validity of the measurements in the population studied. The authors should specify for each instrument the version, language, translation/adaptation process, evidence of validation in the Nepalese population, cut-off point, rationale for the cut-off point, relevant psychometric properties, treatment of missing data, and scoring method. If the cut-off points were developed for another population, this should be explicitly acknowledged as a limitation.

**Response:**
Thank you for this important methodological comment. We have substantially expanded the description of the measurement instruments in the Methods section.

For each instrument, we now report the version used, language of administration, translation and contextual adaptation procedures, scoring approach, cut-off values and the rationale/source for the selected thresholds. We have also clarified how incomplete responses were handled during data cleaning and analysis.

**Reviewer 4.6: Statistical Analysis**

**Reviewer comment:**
The statistical section requires greater specificity. The manuscript does not allow the following to be fully reconstructed: which statistical tests were used; how the rounds were compared; which measures of association were calculated; how continuous variables were handled; how missing data were handled; whether adjusted analyses were performed; how covariates were selected; and whether sensitivity analyses were performed. The authors should fully expand the statistical plan and specify tests used for each comparison, effect measures, confidence intervals, treatment of missing data, criteria for covariate inclusion, relevant stratified analyses, sensitivity analyses, and handling of multiple comparisons when applicable.

**Response:**
Thank you for this important comment. We reviewed the quantitative analysis and agree that the previous description did not sufficiently distinguish the descriptive analysis from inferential statistical analysis.

Importantly, **no inferential statistical tests were performed in this evaluation**. The quantitative results were based on descriptive statistics, including frequencies, percentages, and descriptive presentation of outcome distributions. We have therefore revised the Statistical Analysis subsection to accurately reflect the analyses that were actually conducted rather than introducing post-hoc inferential procedures.

We have clarified that categorical variables were summarized using frequencies and percentages and that continuous or scale-based measures were summarized using appropriate descriptive statistics. We have also specified how the GHQ-12, HSCL-25, life satisfaction, and happiness measures were summarized according to their prespecified scoring and categorization procedures.

We have further clarified how the evaluation phases were handled. Baseline, midline, and endline assessments comprised independent cross-sectional samples rather than repeated observations of the same individuals. The baseline and midline findings were therefore presented alongside endline findings as **descriptive comparisons of observed population-level estimates**. No formal statistical test was used to assess differences between phases, and the observed trends are not interpreted as statistically significant changes or causal intervention effects.

No measures of association, adjusted regression models, covariate-selection procedures, formal stratified inferential analyses, or sensitivity analyses were performed. We have explicitly stated this in the revised Methods section. Similarly, no formal correction for multiple comparisons was applied because the analysis was descriptive and no hypothesis-testing framework was used.

We have also clarified the treatment of missing data. Missing observations were assessed during data cleaning, and analyses for individual variables were based on available observations. No statistical imputation was performed.

These revisions provide a more accurate and reproducible description of the analytical approach actually used in the evaluation and ensure that the manuscript does not imply inferential analyses that were not conducted.

**Reviewer 4.7: Qualitative Component**

**Reviewer comment:**
The description of the qualitative component does not reach the level of detail necessary for reproducibility. It is not sufficiently clear how participant selection, number of interviews/groups, saturation, coding, development of categories, triangulation, participation of different researchers, and resolution of discrepancies were conducted.

**Response:**
Thank you for this important comment. We have substantially expanded the description of the qualitative methodology and analysis to improve transparency and reproducibility.

We now clearly describe the qualitative participant groups and sampling approach. The qualitative component included key informant interviews with local government officials and health-sector personnel and focus group discussions with self-help group and peer support group members. The revised Methods specifies the composition and distribution of the qualitative sample, including **14 KIIs and seven FGDs**, covering the seven participating municipalities.

We have also clarified that participants were purposively selected based on their roles, experiences, and relevance to the evaluation objectives. The qualitative sample was guided by planned coverage of relevant beneficiary and stakeholder groups and the principle of data saturation.

The data-processing procedures are now described in greater detail. Audio recordings were transcribed in Nepali and translated into English by trained translators. Transcripts were anonymized and reviewed for completeness, accuracy, and contextual clarity before analysis.

For analysis, we have explicitly described the five stages of framework analysis: familiarization, development of the thematic framework, indexing/coding, charting, and mapping and interpretation. The initial framework was developed deductively from the evaluation objectives and OECD-DAC evaluation criteria, while inductive coding allowed additional themes and concepts to emerge from the data. Related codes were subsequently grouped into categories, subthemes, and overarching themes.

We have also clarified researcher involvement in the analysis. Two researchers independently coded a subset of transcripts. Differences in coding or interpretation were discussed and resolved through consensus, and the coding framework was refined during the analysis process where necessary.

To strengthen credibility, we have described triangulation across respondent groups and qualitative data sources, as well as integration of qualitative findings with the quantitative findings during interpretation. This allowed us to examine areas of convergence and divergence and to use qualitative evidence to contextualize unexpected or contrasting quantitative findings.

Finally, we have described additional quality-assurance procedures, including training of research assistants, field supervision, daily debriefing, transcript review, independent coding, consensus-based resolution of discrepancies, and triangulation. These changes have been incorporated into the revised **Methods – Qualitative Data Analysis** section.

4.8 Mixed method integration

Response: New section has been added to result section.

**Response to Reviewer 4.9: Ethical approval**

Thank you for identifying this important issue. We agree that the ethical approval date should be explicitly reported and that it should be consistent with the study data collection period.

We have reviewed the ethical approval documentation and have added the exact approval date to the revised manuscript. Ethical approval for the study was granted by the Nepal Health Research Council (NHRC; Ref. 1088) on Jan 13 2026, before the commencement of participant recruitment and data collection on 3 February 2026. We have also corrected the manuscript to consistently report the endline data collection period as 3–26 February 2026.

The revised Ethics statement now specifies the NHRC reference number and approval date. We will also provide the ethical approval letter/certificate as supplementary documentation, subject to PLOS One's requirements and the conditions specified by NHRC regarding dissemination of the approval document.

**Response to Reviewer 4.10: Consent and minor participants**

Thank you for raising this important ethical clarification. We agree that the procedures for participation of adolescents should be described more explicitly.

We have revised the Methods and Ethical Considerations sections to clarify the age eligibility, consent and assent procedures, and ethical safeguards for participants below 18 years of age. Adolescents were included only if they met the eligibility criteria specified in the approved study protocol. For participants younger than 18 years, written informed consent was obtained from a parent or legally authorized guardian, and assent was obtained from the adolescent before participation. The study procedures, including the purpose of the study, voluntary nature of participation, confidentiality, and the right to decline or withdraw, were explained in age-appropriate language before assent was obtained. Adolescents were not enrolled if the required parental/guardian consent or participant assent was not provided.

We have also clarified that the inclusion of participants below 18 years was covered under the ethical approval granted by the Nepal Health Research Council (NHRC; Ref. 1088). The revised manuscript now reports the relevant age range and consent/assent procedure explicitly.

**Reviewer 5: Results**

**Comment 5.1: Sample characterization and transparency of denominators**

**Reviewer comment:**
Table 1 should provide a more complete characterization of the study population, including differentiation between migrant workers, family members remaining in Nepal, and other groups. The reviewer also requested a cross-sectional review of Tables 1–3 to ensure clearly identifiable denominators.

**Response:**
Thank you for this important observation. We agree that the original presentation did not sufficiently distinguish the composition of the endline sample or consistently identify the denominator for subgroup variables. We have revised the Results and tables to improve transparency.

Table 1 has been expanded to include migration-related household information. In particular, we now report that 216 of 245 participants (88.2%) reported having a family member currently working abroad, while 29 (11.8%) did not. We also report the relationship with the family member abroad using the subgroup denominator of 216.

We have also clarified in both the Methods and Results that the available quantitative data do not provide a sufficiently clear mutually exclusive classification of all 245 participants into migrant workers, left-behind family members, persons with psychosocial disabilities, SHG members, and other beneficiary groups. We therefore no longer present the sample as representative of all Nepalese migrant workers. The manuscript now explicitly states that the findings primarily describe project beneficiaries and migration-affected households in the participating municipalities.

We have also acknowledged the marked sex imbalance, with 231 of 245 participants (94.3%) being female, and its implications for generalizability, particularly to male migrant workers and returnees.

In addition, Tables 1–3 have been reviewed for denominator consistency. Variables assessed in subgroups are now presented using n/N (%) and the relevant denominator is identified in the table footnotes.

**Changes made:**

- Revised Methods, Section 2.3, to clarify the study population and external validity.
- Expanded and revised Table 1.
- Revised Results, Section 3.1.1.
- Revised Tables 2 and 3 to identify subgroup denominators.
- Strengthened the Strengths and Limitations section regarding sex imbalance, sample composition, and generalizability.

**Reviewer Comment 6.2: Each Main Result Should Be Discussed Against the Evidence**

**Reviewer comment:**
The Discussion should explicitly address the main findings, including anxiety and depression, psychological distress, and life satisfaction. The authors should compare the magnitude of the findings with previous studies among migrant workers, Nepalese populations, migrant families, and similar low- and middle-income contexts. Relevant systematic reviews and meta-analyses should also be incorporated. The increase in psychological distress should receive specific discussion and should not be subordinated to favourable findings for anxiety and depression. The observed difference in life satisfaction requires a broader and more balanced interpretation. The authors should avoid a selective narrative focused exclusively on favourable results.

**Response:**
Thank you for this important observation. We agree that the previous version of the Discussion placed greater emphasis on the lower proportions of anxiety and depression and did not provide sufficiently balanced interpretation of the increased proportion of participants screening positive for psychological distress or the differences observed in life satisfaction.

We have substantially revised the Discussion to address each major outcome separately and to interpret the findings in relation to the accumulated evidence. Specifically:

1. **Anxiety and depression:** We now compare the endline estimates with evidence from systematic reviews and meta-analyses of migrant workers, including studies from low- and middle-income settings, as well as evidence concerning Nepalese migrant workers and migration-affected families. We also clarify that direct comparison of prevalence estimates is limited by differences in populations, instruments, cut-offs, sampling strategies, and study designs.
2. **Psychological distress:** We have added a dedicated subsection discussing the GHQ-12 finding. The Discussion now explicitly recognizes that the 34.7% endline estimate of probable psychological distress contrasts with the lower proportions screening positive for anxiety and depression. We explain that GHQ-12 and HSCL-25 assess related but distinct dimensions of psychological wellbeing and therefore should not be treated as interchangeable outcomes.
3. **Life satisfaction:** We have expanded the interpretation of life satisfaction and avoided presenting the observed difference as evidence of individual-level deterioration. We now emphasize that the assessment phases involved independent samples and that life satisfaction was not assessed at baseline. The revised Discussion considers broader socioeconomic and migration-related circumstances that may influence life satisfaction while explicitly acknowledging that these mechanisms were not measured or established by the present study.
4. **Intervention evidence:** We have incorporated relevant systematic reviews and meta-analyses on community-based psychological interventions, psychosocial interventions among migrants and refugees, and psychological treatments delivered by nonspecialist providers in low- and middle-income countries. These studies are used to contextualize the plausibility and relevance of community-based MHPSS rather than to claim that the observed differences were caused by the PARBARDHAN intervention.
5. **Balanced interpretation:** The revised Discussion now presents the findings as a heterogeneous pattern of psychosocial wellbeing rather than as uniformly favourable change. In particular, the coexistence of lower anxiety and depression screening estimates with higher general psychological distress and less favourable life-satisfaction distributions is explicitly discussed.

These changes have been incorporated in **Sections 6.2–6.6 of the revised Discussion**.

**Reviewer Comment 6.3: Interpretation of Mechanisms**

**Reviewer comment:**
The manuscript proposes explanations such as economic difficulties, job insecurity, and greater recognition of distress. These explanations are plausible, but the study does not necessarily demonstrate them. The authors should use explicitly hypothetical language such as “may reflect,” “may be explained by,” and “one possible explanation is...” and avoid presenting unmeasured mechanisms as established explanations.

**Response:**
We appreciate this clarification and agree that some mechanisms proposed in the previous Discussion were stated too definitively.

We have revised the Discussion throughout to distinguish between **observed findings, participant-reported experiences, and possible explanations**. Statements concerning economic difficulties, employment insecurity, family separation, greater mental health awareness, and increased recognition or reporting of distress are now explicitly presented as hypotheses rather than demonstrated mechanisms.

**Reviewer Comment 6.4: Comparison with Systematic Reviews and Meta-analyses**

**Reviewer comment:**
The Discussion should make much better use of available secondary literature, particularly systematic reviews and meta-analyses addressing prevalence, intervention effects, psychosocial support, community interventions, stigma, and mental health among migrant workers. This should be used to interpret the findings against the accumulated body of evidence rather than simply increasing the number of references.

**Response:**
Thank you. We agree that the previous Discussion did not sufficiently position the findings within the broader evidence base.

We have therefore incorporated and critically discussed relevant systematic reviews and meta-analyses in the revised Discussion. These sources are used for several specific purposes rather than simply to increase the number of citations.

**Reviewer Comment 6.5: Limitations**

**Reviewer comment:**
The current limitations should be expanded to explicitly include cross-sectional design, independent samples across phases, absence of a control group, inability to establish causality, possible selection bias, sex imbalance, population heterogeneity, possible differences between rounds, limitations of the tools used, possible confounding factors, retrospective information when applicable, and limited generalizability. The authors should explain how each limitation specifically affects interpretation rather than simply listing them.

**Response:**
We agree with the reviewer. The limitations section has been substantially expanded to address each of these issues and, importantly, to explain their implications for interpretation.

The revised manuscript now explicitly addresses the following:

- **Cross-sectional design:** We clarify that the study provides estimates at each assessment phase but cannot establish individual-level change or temporal causality.
- **Independent samples across phases:** We explicitly state that baseline, midline, and endline participants were not necessarily the same individuals. Therefore, differences between phases cannot be interpreted as within-person improvement or deterioration.
- **Absence of a control group:** We explain that without a comparison group, changes cannot be separated from secular trends, contextual changes, other interventions, or differences in participant composition.
- **Causal inference:** We now explicitly state that the evaluation cannot establish that PARBARDHAN caused the observed differences in anxiety, depression, psychological distress, or life satisfaction.
- **Possible selection bias:** We acknowledge that differences in participant availability, willingness to participate, or beneficiary composition across assessment rounds may have influenced the observed estimates.
- **Sex imbalance:** We report that the endline sample was predominantly female and explain that this limits generalizability, particularly to male migrant workers and returnee migrants.
- **Population heterogeneity:** We clarify that the study population comprised project-associated and migration-affected participants and was not a homogeneous sample of migrant workers. Consequently, findings should not be generalized to all Nepalese migrant workers.
- **Differences between assessment rounds:** We explain that differences in sample composition, sample size, and contextual circumstances may partly account for differences in outcome distributions.
- **Measurement limitations:** We clarify that GHQ-12 and HSCL-25 are screening instruments rather than diagnostic assessments. A positive screening classification therefore should not be interpreted as a clinically confirmed diagnosis.
- **Possible confounding:** We acknowledge that factors including socioeconomic circumstances, migration history, disability, family circumstances, employment conditions, and exposure to other programmes may influence psychosocial outcomes. Because the quantitative analysis was descriptive and did not include multivariable modelling, their independent effects could not be assessed.
- **Retrospective information:** We acknowledge the potential for recall and social desirability bias in self-reported information and participant accounts of experiences or perceived changes.
- **Limited generalizability:** We explicitly state that the evaluation was conducted among project-associated participants in selected municipalities of Salyan and Kailali and that the findings should not be considered representative of all migrant workers or migrant families in Nepal.

We have therefore revised the limitations section so that each limitation is directly connected to the interpretation of the study findings rather than being presented as a standalone list.

**Reviewer Comment 7: Conclusions**

Reviewer comment:
The current conclusion presents the program as if the results demonstrated that PARBARDHAN “effectively addressed” psychosocial well-being. This exceeds what can reasonably be concluded from the described design.

Response:
Thank you for this important comment. We agree that the conclusion should clearly distinguish between the observed findings and evidence of causal programme effectiveness.

We reviewed and revised the conclusion to further strengthen this distinction. The revised conclusion now explicitly states that the findings were not uniformly favorable across psychosocial outcomes, noting that lower proportions screening positive for anxiety and depression coexisted with a higher proportion screening positive for probable psychological distress and a substantially different distribution of life satisfaction between assessment rounds.

We have also revised the interpretation of the qualitative findings to state that they are “consistent with potential benefits and perceived usefulness of community-based MHPSS support” rather than demonstrating programme effectiveness.

In addition, the revised conclusion explicitly identifies the absence of a comparator group, together with the use of independent cross-sectional samples and descriptive quantitative analysis, as reasons why the observed differences cannot be interpreted as causal effects of PARBARDHAN. We have retained the statement concerning limited generalizability to all migrant workers and their families in Nepal and strengthened the recommendation for future longitudinal evaluations with individual follow-up and appropriate comparison groups.

**Reviewer Comment 8.1: Ethical Approval**

**Reviewer comment:**
The authors should provide the exact date of NHRC approval (Ref. 1088), confirm that it preceded recruitment and data collection, and provide supporting documentation.

**Response:**
Thank you for highlighting the need for greater transparency regarding ethical approval. We have revised the Ethics Statement to provide the NHRC reference number and the exact approval date based on the official NHRC approval documentation.

The revised manuscript now explicitly confirms that ethical approval was obtained from the Nepal Health Research Council (NHRC; Ref. No. 1088) before participant recruitment and commencement of data collection.

We have also provided the relevant NHRC ethical approval documentation as a supplementary file to support this statement.

**Reviewer Comment 8.2: Informed Consent**

**Reviewer comment:**
The authors should expand the description of informed consent, particularly for adolescents.

**Response:**
Thank you. We have expanded the informed-consent description in the Methods section.

The revised manuscript now specifies that adult participants provided written informed consent after receiving information about the study, including its purpose, procedures, potential risks and benefits, confidentiality, voluntary participation, and the right to decline participation or withdraw without consequence.

For participants below 18 years of age, we have clarified that participation required **written consent from a parent or legally authorized guardian together with assent from the adolescent participant**. The adolescent was provided with age-appropriate information about the study and participation was voluntary. We have also clarified that refusal or withdrawal did not affect access to available services or other benefits.

The revised text distinguishes parental/guardian permission from the adolescent's own assent and makes clear that both were obtained for adolescent participation.

**Reviewer Comment 8.3: Data Availability**

**Reviewer comment:**
The authors should review the data availability statement to ensure that it is consistent with PLOS policies. If ethical or legal restrictions prevent the deposit of individual-level data, the authors should specifically explain which data are restricted, the ethical/legal basis, who may request access, through what procedure, and under what conditions access would be granted. A generic “available upon request” statement should be avoided if it does not satisfy the applicable policies.

**Response:**
We agree with the reviewer that the previous data availability statement required greater specificity.

We have revised the Data Availability Statement to distinguish between the de-identified individual-level research data and the supporting study materials. Because the individual-level dataset contains potentially sensitive information relating to mental health, disability, migration status, family circumstances, and other participant characteristics, unrestricted public deposition may pose risks to participant confidentiality and is subject to the ethical conditions under which the study was approved.

Accordingly, the revised statement specifies the nature of the restricted data, the ethical basis for restricting public access, and the process through which qualified researchers may request access. Any data sharing will be subject to review to ensure that the proposed use is consistent with the original ethical approval, informed-consent conditions, applicable privacy requirements, and protection of participant confidentiality.

We have therefore replaced the previous generic “available upon request” wording with a more detailed Data Availability Statement that specifies the responsible institutional contact/process and the conditions under which access may be considered.

**Revised manuscript location:** Data Availability Statement.

**Reviewer Comment 9.1: Number and Coverage of References**

**Reviewer comment:**
The evaluated version contains a relatively limited bibliography for the number of concepts addressed. The authors should comprehensively review the references and incorporate current, high-quality literature on migration and mental health, migrant workers, migrant families, MHPSS interventions, community interventions, peer support, stigma, systematic reviews, and meta-analyses. Central statements in the Introduction and Discussion should be supported by directly relevant references.

**Response:**
Thank you for this important recommendation. We agree that the previous version did not provide sufficiently comprehensive coverage of the evidence underlying several key statements in the Introduction and Discussion.

We have therefore undertaken a comprehensive review of the reference base and revised the manuscript selectively rather than increasing the number of citations solely for the purpose of expanding the bibliography.

The revised manuscript now incorporates more directly relevant and recent evidence in the following areas:

1. **Migration and mental health:** We have strengthened the background literature describing the relationship between migration-related circumstances, family separation, socioeconomic conditions, and mental health.
2. **Migrant workers:** We have incorporated recent systematic reviews and meta-analyses describing the prevalence and determinants of anxiety, depression, psychological distress, and related mental health outcomes among migrant workers.
3. **Migrant families and left-behind populations:** We have added evidence concerning the psychosocial consequences of migration and family separation, while clearly distinguishing these populations from the heterogeneous population included in the present study.
4. **MHPSS interventions:** We have incorporated systematic reviews and meta-analyses examining psychosocial and psychological interventions among migrant, refugee, and other vulnerable populations.
5. **Community-based interventions:** We have added evidence concerning community-based mental health interventions and interventions delivered by nonspecialist providers, particularly in low- and middle-income settings.
6. **Peer and self-help support:** Relevant literature has been incorporated to contextualize the qualitative findings concerning peer support, self-help groups, and community-based psychosocial support.
7. **Stigma and mental health awareness:** The revised Discussion includes evidence concerning stigma, mental health literacy, help-seeking, and barriers to accessing mental health services.
8. **Systematic reviews and meta-analyses:** We have prioritized systematic reviews and meta-analyses where these provide more appropriate evidence for interpreting prevalence estimates and intervention effects, while avoiding direct comparisons when populations, instruments, or study designs are substantially different.

We have also revised the Introduction and Discussion so that key epidemiological and intervention-related claims are supported by references that are directly relevant to the specific statements being made.

Importantly, we have avoided treating the broader literature as confirmation of programme effectiveness. Instead, previous evidence is used to contextualize the magnitude and direction of our findings and to identify areas of agreement, difference, and uncertainty.

**Location in revised manuscript:** Introduction and Sections 6.2–6.7 of the Discussion.

**Reviewer Comment 9.1: Recent Methodological Literature on the Assessment of Depressive and Anxiety Symptoms**

**Reviewer comment:**
The authors should consider recent methodological literature concerning the psychometric properties, reliability, convergent validity, and measurement equivalence of widely used instruments for assessing depressive and anxiety symptoms. This is particularly important when comparing populations, countries, sociodemographic groups, or cultural contexts. The reviewer specifically suggests recent studies concerning the PHQ-9, PHQ-2, and GAD-7.

**Response:**
We appreciate this methodological recommendation and agree with the underlying concern that differences in observed symptom estimates may reflect differences in measurement properties as well as differences in the underlying construct.

We have incorporated this methodological perspective into the revised Discussion and clarified the implications for interpretation of cross-phase and cross-study comparisons. In particular, we now emphasize that differences in prevalence estimates across studies should be interpreted cautiously because measurement instruments, language versions, cut-off values, cultural context, respondent characteristics, and study populations may differ.

We have also selectively considered the recent methodological literature suggested by the reviewer concerning factorial invariance, reliability, and convergent validity of commonly used depression and anxiety screening instruments. We use this literature to support the **general methodological principle of measurement comparability**, rather than to imply that studies of PHQ-9, PHQ-2, or GAD-7 directly establish the validity of the GHQ-12 or HSCL-25 instruments used in the present study.

Because the present study used the **GHQ-12 and HSCL-25 rather than PHQ-9, PHQ-2, or GAD-7**, we have avoided making claims that the suggested studies directly validate our instruments. Where relevant, instrument-specific literature concerning the GHQ-12 and HSCL-25 has been prioritized for methodological justification and interpretation.

We have also clarified in the revised manuscript that GHQ-12 and HSCL-25 are **screening instruments rather than diagnostic instruments**, and that positive classifications should not be interpreted as clinically confirmed anxiety or depressive disorders.

Finally, we have moderated comparisons of prevalence estimates across studies and explicitly acknowledge that differences in measurement instruments and cut-offs can limit direct comparability.

**Location in revised manuscript:** Methods, particularly the description of the mental health instruments, and Sections 6.2–6.3 of the Discussion.

**Reviewer 9.2. Literature Update**

**Reviewer Comment:**
Although older references may be appropriate when they constitute foundational works, the manuscript requires a substantial update of the recent literature. The authors should update the bibliography with contemporary evidence, prioritizing recent and methodologically robust studies and reviews, without removing classic references that remain necessary.

**Response:**
We thank the reviewer for this important recommendation. We agree that the reference base should better reflect recent evidence, particularly given the rapidly evolving literature on migration, mental health, psychosocial wellbeing, community-based MHPSS, and migrant and left-behind families.

We therefore conducted a comprehensive update of the bibliography and incorporated recent peer-reviewed studies, systematic reviews, and meta-analyses published in recent years. The revised literature base includes contemporary evidence on mental health among migrant workers and migrant families, psychosocial wellbeing in migration-affected populations, community-based psychological and psychosocial interventions, peer and self-help approaches, stigma and access to mental health services, and relevant methodological considerations for mental health screening.

Recent systematic reviews and meta-analyses were prioritized where available because they provide a stronger basis for contextualizing the findings than individual studies alone. At the same time, foundational and historically important references were retained where they remain directly relevant to the theoretical framework, measurement approaches, or established concepts used in the study.

We also reviewed the Introduction and Discussion to ensure that recently published evidence is used where appropriate rather than relying predominantly on older literature.

**Revised manuscript location:**
References throughout the **Introduction and Discussion**, with corresponding additions and updates to the **Reference list**.

**Reviewer 9.3. Correspondence Between Claims and References**

**Reviewer Comment:**
Some general statements regarding prevalence, effectiveness, mechanisms, and program benefits require a more direct correspondence with the cited evidence. The authors should review each substantive statement in the Introduction and Discussion and verify that the cited reference actually supports the claim being made.

**Response:**
We appreciate this important observation and agree that the correspondence between substantive claims and supporting references needed to be strengthened. We conducted a claim-by-claim review of the Introduction and Discussion and revised statements where the original wording was broader than the evidence provided by the cited reference.

In the revised manuscript, references are now linked more directly to the specific claims they support. In particular:

1. **Prevalence estimates:** Statements concerning the prevalence of anxiety, depression, psychological distress, and other mental health outcomes among migrant workers and migration-affected populations have been linked to directly relevant studies and systematic reviews. We have avoided presenting estimates from different populations or instruments as directly comparable where important methodological differences exist.
2. **Intervention effectiveness:** Claims concerning the effectiveness of community-based MHPSS, psychological interventions, peer support, or related approaches are now supported primarily by systematic reviews, meta-analyses, and intervention studies where appropriate. We have also distinguished evidence demonstrating intervention effectiveness from evidence describing perceived usefulness or acceptability.
3. **Mechanisms and explanatory pathways:** Statements about possible mechanisms linking migration, family separation, socioeconomic conditions, social support, and mental health have been revised to avoid presenting proposed mechanisms as established causal pathways unless directly supported by the cited evidence. Where evidence is observational, the manuscript now uses appropriately cautious language such as “may,” “may be associated with,” or “one possible explanation.”
4. **Program and intervention benefits:** We revised statements that could be interpreted as demonstrating a causal benefit of the PARBARDHAN intervention. Because the evaluation used independent cross-sectional samples, descriptive quantitative analyses, and no comparator group, the findings cannot establish causal intervention effects. Qualitative findings are therefore presented as participants’ experiences and perceptions of potentially useful components of community-based MHPSS rather than as evidence of quantified program effectiveness.
5. **Measurement-related claims:** We have also reviewed references supporting the use and interpretation of mental health screening measures. The revised manuscript makes clear that the GHQ-12 and HSCL-25 were used as **screening measures** and that positive screening results should not be interpreted as clinical diagnoses. Recent methodological literature on measurement reliability and comparability has been considered where relevant, while avoiding the use of evidence concerning instruments such as PHQ-9 or GAD-7 as though it directly validated the instruments used in this study.
6. **Citation specificity:** General statements have been narrowed where necessary so that each citation supports the specific population, outcome, intervention, or methodological claim being made. Where a single reference did not adequately support a broad statement, the statement was either revised to a more precise claim or supported with additional appropriate references.

Overall, these revisions were intended to ensure that the manuscript distinguishes clearly between **what previous studies have demonstrated, what the present study observed, what participants reported, and what can reasonably be inferred from the findings**.

**Revised manuscript location:**
Substantive revisions throughout the **Introduction and Discussion**, with corresponding updates to the **Reference list**.

**Response to Reviewer 6, Major Comments**

**Reviewer Comment:**
I consider that the following points must be addressed as a major revision: [Points 1–16].

**Response:**
We sincerely thank the reviewer for providing this comprehensive and constructive assessment of the manuscript. We agree that the issues identified are important for ensuring that the manuscript accurately reflects the study design, population, analytical approach, and strength of the evidence. We have undertaken a substantial revision of the manuscript in response to these recommendations.

The major revisions are summarized below.

1. **Title:** The title has been revised to more accurately describe the study population, geographical setting, and cross-sectional evaluation design, without implying individual-level change or intervention effectiveness.
2. **Abstract:** The Abstract has been substantially revised. Statements implying intervention effectiveness or causality have been removed. The design is now explicitly described as a cross-sectional mixed-methods evaluation with comparisons across independent assessment rounds, and the interpretation has been aligned with the descriptive nature of the quantitative analysis.
3. **Introduction:** The Introduction has been substantially restructured to provide a stronger conceptual and epidemiological framework concerning migration, family separation, mental health, psychosocial wellbeing, and community-based MHPSS. The knowledge gap has also been clarified, particularly the limited evidence from migration-affected communities in Nepal and the need to examine multiple dimensions of psychosocial wellbeing rather than focusing exclusively on anxiety or depression.
4. **Secondary evidence:** Recent systematic reviews and meta-analyses have been incorporated to strengthen the epidemiological and intervention context. These include evidence concerning mental health among migrant workers and migrant families, community-based psychological interventions, psychosocial interventions, and related migrant and displacement populations. Where relevant, evidence syntheses have been prioritized over isolated individual studies.
5. **Study design and retrospective elements:** The Methods section now provides a clearer explanation of the study design. The term “retrospective” has been clarified so that it does not imply retrospective cohort follow-up or individual-level longitudinal analysis. The baseline, midline, and endline assessments consisted of independent cross-sectional samples, and comparisons across rounds are therefore interpreted as differences in the distributions of the sampled populations rather than changes within individuals.
6. **Comparison across assessment rounds:** The manuscript now explicitly identifies the sample sizes and composition of the baseline, midline, and endline assessment rounds and explains how the reported comparisons were made. The presentation has been revised to avoid implying individual-level trajectories. The “Trend” interpretation has been removed where it could suggest longitudinal change.
7. **Study population:** The composition of the study population has been clarified, including the predominance of participants from migration-affected households at endline. The manuscript now acknowledges the heterogeneity of the eligible population and explains why these groups were included within the broader community-based evaluation. We have also avoided interpreting the endline sample as representative of all migrant workers or all families of migrant workers in Nepal.
8. **Statistical methodology:** The quantitative analysis has been revised to accurately reflect the analyses actually conducted. The manuscript now describes descriptive analysis rather than inferential testing. Frequencies and percentages are presented for categorical variables, with denominators specified where they differ because of missing responses or variable-specific eligibility. We have not added statistical tests, effect estimates, confidence intervals, or sensitivity analyses that were not conducted in the original analysis. This clarification is important to avoid implying an analytical procedure that was not performed.
9. **Qualitative component and mixed-methods integration:** The qualitative Methods and Results sections have been strengthened to describe the KIIs, FGDs, analytical framework, coding process, and integration with the quantitative findings more clearly. Qualitative findings are used to provide contextual explanation and participants' perspectives alongside the quantitative findings, rather than being treated as evidence of intervention effectiveness or causal mechanisms.
10. **Suicide and effectiveness claims:** Statements derived from qualitative testimony concerning suicide and program effectiveness have been carefully revised. Participant accounts are presented as perceptions and experiences rather than objective evidence that the intervention prevented suicide attempts or produced specific mental health outcomes. Causal language concerning the PARBARDHAN intervention has been removed or appropriately qualified.
11. **Discussion:** The Discussion has been substantially reconstructed. Each major quantitative finding is now considered in relation to relevant previous evidence, including systematic reviews and meta-analyses. Particular attention has been given to the apparently discordant pattern of lower anxiety and depression screening positivity alongside higher probable psychological distress and differences in life satisfaction. Possible explanations are presented as hypotheses rather than established mechanisms.
12. **Limitations:** The limitations section has been expanded substantially. It now explicitly addresses the cross-sectional design, independent samples across assessment rounds, absence of a comparator group, inability to establish causality or individual-level change, possible selection bias, substantial sex imbalance, heterogeneity of the study population, differences in sample composition across rounds, use of screening instruments rather than diagnostic assessments, potential confounding, self-reported and retrospective information, and limited generalizability.
13. **Conclusions:** The Conclusions have been reformulated to ensure that they are proportional to the study design and evidence. The findings are described as heterogeneous rather than uniformly favorable. Lower anxiety and depression screening positivity is presented alongside higher probable psychological distress and differences in life satisfaction. The qualitative findings are described as indicating perceived usefulness and acceptability of community-based MHPSS components rather than demonstrating intervention effectiveness.
14. **Ethical approval:** The Ethics section has been revised to provide the NHRC approval reference and the exact approval date based on the official ethical approval documentation. The revised text also clarifies the temporal relationship between ethical approval and participant recruitment/data collection. We have additionally expanded the description of informed consent and assent procedures for adult and adolescent participants.
15. **Data availability:** The Data Availability Statement has been completely reviewed and revised in accordance with PLOS requirements. The revised statement distinguishes individual-level participant data from non-identifying study materials and explains the ethical and confidentiality considerations governing access to the individual-level dataset. The procedure and conditions for requesting access have been specified rather than relying solely on a generic “available upon request” statement.
16. **References:** The reference list has been substantially updated. Recent peer-reviewed studies, systematic reviews, and meta-analyses have been incorporated, while foundational references have been retained where they remain necessary. We also conducted a citation audit to improve the correspondence between substantive claims and the evidence cited.

**Minor Comments**

**Reviewer Comment:**
The reviewer also requested a complete linguistic review, terminology standardization, consistency across manuscript sections, verification of denominators and percentages, independent interpretability of tables, definition of abbreviations, consistency of statistical presentation, traceability of results, reference cross-checking, and review of supplementary materials.

**Response:**
We thank the reviewer for these detailed editorial recommendations. We conducted a comprehensive consistency and language review of the revised manuscript. Specifically:

1. The manuscript has undergone a comprehensive **linguistic and grammatical review** to improve clarity, readability, and academic style.
2. Terminology has been standardized throughout the manuscript. The revised text distinguishes between **mental health**, **psychosocial wellbeing**, **psychological distress**, and screening indicators of **anxiety and depression**. Screening-positive results are not described as clinical diagnoses.
3. The **title, abstract, objectives, methods, results, discussion, and conclusions** have been cross-checked to ensure consistency with the cross-sectional design and descriptive analysis.
4. **Tables and denominators have been reviewed**, including variable-specific denominators where missing responses or eligibility criteria result in different analytical sample sizes. Percentages have been recalculated and checked against the reported numerators.
5. Table titles, headings, footnotes, abbreviations, denominators, and explanatory notes have been revised so that each table can be interpreted independently.
6. Abbreviations are now defined at first use and used consistently thereafter.
7. The presentation of **sample sizes, percentages, cut-offs, and statistical terminology** has been standardized throughout the manuscript.
8. Results reported in the narrative text have been cross-checked against the corresponding tables to improve traceability and avoid reporting results that cannot be located in the quantitative presentation.
9. The **in-text citations and reference list** have been cross-checked for consistency, with missing or unnecessary references corrected where identified.
10. The supplementary materials have been reviewed to ensure that methodological and ethical documents referred to in the manuscript are appropriately identified and available where permitted by ethical and institutional requirements.

We appreciate the reviewer’s detailed comments, which helped us substantially improve the manuscript's methodological transparency, interpretation, and overall consistency. The revised manuscript now more clearly distinguishes **descriptive findings from causal inference, participant perceptions from demonstrated intervention effects, and differences between independent assessment rounds from individual-level change**.
