# Supplementary material for: Mental health and psychosocial wellbeing among beneficiaries of a community-based intervention in Nepal: Findings from a cross-sectional endline evaluation of the PARBARDHAN project
Source: PLOS Glob Public Health. 2026 Oct 16;6(10):e0006666. doi: 10.1371/journal.pgph.0006666 (PMC13674775; doi:10.1371/journal.pgph.0006666)
Supplement: Peer review history

# Author comment: Author response to Decision Letter 0

**DOI:** 10.1371/journal.pgph.0006666.r001

21 Apr 2026

Attachment Submitted filename: Resopnse to Reviewers.docx

# Peer review: Decision Letter 0

**DOI:** 10.1371/journal.pgph.0006666.r002

**Authors:** Md. Alamgir Hossain

22 May 2026

PGPH-D-26-01439

Mental Health and Psychosocial Wellbeing of Migrant Workers and their left behind families in Nepal: Insights from the PARBARDHAN Endline Evaluation

PLOS Global Public Health

Dear Dr. Gaire,

Thank you for submitting your manuscript to PLOS Global Public Health. After careful consideration, we feel that it has merit but does not fully meet PLOS Global Public Health’s publication criteria as it currently stands. Therefore, we invite you to submit a revised version of the manuscript that addresses the points raised during the review process.

Please submit your revised manuscript by 25/06/2026. If you will need more time than this to complete your revisions, please reply to this message or contact the journal office at globalpubhealth@plos.org. When you're ready to submit your revision, log on to https://www.editorialmanager.com/pgph/ and select the 'Submissions Needing Revision' folder to locate your manuscript file.

Please include the following items when submitting your revised manuscript:

- A letter that responds to each point raised by the editor and reviewer(s). You should upload this letter as a separate file labeled 'Response to Reviewers'.
- A marked-up copy of your manuscript that highlights changes made to the original version. You should upload this as a separate file labeled 'Revised Manuscript with Track Changes'.
- An unmarked version of your revised paper without tracked changes. You should upload this as a separate file labeled 'Manuscript'.

Guidelines for resubmitting your figure files are available below the reviewer comments at the end of this letter.

As the corresponding author, your ORCID iD is verified in the submission system and will appear in the published article. PLOS supports the use of ORCID, and we encourage all coauthors to register for an ORCID iD and use it as well. Please encourage your coauthors to verify their ORCID iD within the submission system before final acceptance, as unverified ORCID iDs will not appear in the published article. *Only*  the individual author can complete the verification step; PLOS staff *cannot*  verify ORCID iDs on behalf of authors.

We look forward to receiving your revised manuscript.

Kind regards,

Md. Alamgir Hossain, M.S.S., M.P.H.

Academic Editor

PLOS Global Public Health

**Journal Requirements:**

1. Please provide additional information regarding the considerations  made for the migrant workers included in this study. For instance, please discuss whether participants were able to opt out of the study and whether individuals who did not participate receive the same treatment offered to participants.

2. You indicated that you had ethical approval for your study. In your Methods section, please ensure you have also stated whether you obtained consent from parents or guardians of the minors included in the study or whether the research ethics committee or IRB specifically waived the need for their consent.

3. Please amend your detailed Financial Disclosure statement. This is published with the article. It must therefore be completed in full sentences and contain the exact wording you wish to be published.

i. Please clarify all sources of financial support for your study. List the grants, grant numbers, and organizations that funded your study, including funding received from your institution. Please note that suppliers of material support, including research materials, should be recognized in the Acknowledgements section rather than in the Financial Disclosure.

ii. State the initials, alongside each funding source, of each author to receive each grant. For example: "This work was supported by the National Institutes of Health (####### to AM; ###### to CJ) and the National Science Foundation (###### to AM)."

iii. State what role the funders took in the study. If the funders had no role in your study, please state: “The funders had no role in study design, data collection and analysis, decision to publish, or preparation of the manuscript.”

iv. If any authors received a salary from any of your funders, please state which authors and which funders.

4. We note that you have indicated that there are restrictions to data sharing for this study. For studies involving human research participant data or other sensitive data, we encourage authors to share de-identified or anonymized data. However, when data cannot be publicly shared for ethical reasons, we allow authors to make their data sets available upon request. For information on unacceptable data access restrictions, please see http://journals.plos.org/plosone/s/data-availability#loc-unacceptable-data-access-restrictions.

Before we proceed with your manuscript, please address the following prompts:

a) If there are ethical or legal restrictions on sharing a de-identified data set, please explain them in detail (e.g., data contain potentially identifying or sensitive patient information, data are owned by a third-party organization, etc.) and who has imposed them (e.g., a Research Ethics Committee or Institutional Review Board, etc.). Please also provide contact information for a data access committee, ethics committee, or other institutional body to which data requests may be sent.

b) If there are no restrictions, please upload the minimal anonymized data set necessary to replicate your study findings to a stable, public repository and provide us with the relevant URLs, DOIs, or accession numbers. Please see http://www.bmj.com/content/340/bmj.c181.long for guidelines on how to de-identify and prepare clinical data for publication. For a list of recommended repositories, please see https://journals.plos.org/plosone/s/recommended-repositories. You also have the option of uploading the data as Supporting Information files, but we would recommend depositing data directly to a data repository if possible.

Please update your Data Availability statement in the submission form accordingly.

5. Please ensure that your Ethics Statement is available in its entirety at the beginning of your Methods section, under a subheading 'Ethics Statement'.

If the reviewer comments include a recommendation to cite specific previously published works, please review and evaluate these publications to determine whether they are relevant and should be cited. There is no requirement to cite these works unless the editor has indicated otherwise.

**Additional Editor Comments (if provided):**

[Note: HTML markup is below. Please do not edit.]

Reviewers' comments:

Reviewer's Responses to Questions

**Comments to the Author**

1. Does this manuscript meet PLOS Global Public Health’s publication criteria?

Reviewer #1: Partly

Reviewer #2: Partly

Reviewer #3: Yes

Reviewer #4: Yes

**********

2. Has the statistical analysis been performed appropriately and rigorously?

Reviewer #1: No

Reviewer #2: No

Reviewer #3: Yes

Reviewer #4: Yes

**********

3. Have the authors made all data underlying the findings in their manuscript fully available (please refer to the Data Availability Statement at the start of the manuscript PDF file)?

The PLOS Data policy

Reviewer #1: Yes

Reviewer #2: No

Reviewer #3: Yes

Reviewer #4: Yes

**********

4. Is the manuscript presented in an intelligible fashion and written in standard English?

Reviewer #1: No

Reviewer #2: No

Reviewer #3: Yes

Reviewer #4: Yes

**********

**Reviewer #1:**  1. Summary of the Manuscript

This manuscript evaluates the impact of the PARBARDHAN project on the mental health and psychosocial wellbeing of migrant workers and their left-behind families in the Salyan and Kailali districts of Nepal. The PARBARDHAN project is a community-based mental health and psychosocial support initiative implemented by CMC-Nepal, targeting migrant workers, their families, and other vulnerable populations.

Using a mixed-methods approach, the study assesses psychosocial distress, social support, coping strategies, stigma, life satisfaction, and happiness, while comparing findings with earlier baseline and midline evaluations.

The topic is highly relevant from a public health and psychosocial perspective, particularly in low-resource and migration-affected settings. The use of mixed methods is also appropriate for the study objectives. However, the manuscript would benefit substantially from improved methodological clarity, stronger statistical analysis, deeper engagement with relevant literature, and more rigorous interpretation of findings.

2. Abstract

The abstract is generally concise and structured. However, several areas require improvement for better coherence and completeness.

� The introduction and study objective appear insufficiently aligned with the title and overall aims of the manuscript.

� The methods section of the abstract would benefit from briefly stating the specific assessment tools used and their intended domains of measurement.

� Including key prevalence estimates or trends reported during baseline and midline assessments would improve contextual continuity and help readers appreciate the significance of the endline findings.

� The age range of study participants should also be stated.

� Due to limited contextual linkage with earlier evaluations, the conclusion appears somewhat abrupt and insufficiently grounded in preceding findings.

3. Major Concerns

Title

The title may benefit from minor restructuring to better reflect the primary aim of the study and improve alignment between the study objectives, methods, and conclusions.

Introduction

The introduction would benefit from greater depth and contextual framing.

Specifically:

� More detailed discussion of the PARBARDHAN project, including its scope, interventions, and implementation context, would strengthen reader understanding.

� The rationale for selecting Salyan and Kailali districts should be clearly justified.

� The manuscript should provide prevalence estimates of psychosocial and mental health challenges identified during baseline and midline assessments, including how these outcomes were measured.

� Additional and more recent literature should be incorporated to strengthen contextual grounding and scholarly engagement.

There are also concerns regarding sparse and inconsistent citation practices. For example, the statement in line 63:

“Previous studies have consistently shown that migrant workers experience higher rates of mental health problems compared to native workers…”

requires stronger and more appropriately contextualised referencing.

Methods and Materials

The mixed-methods design involving quantitative and qualitative components (KII and FGD) is appropriate for the research objectives. However, methodological reporting requires further clarification and refinement.

� The opening statement of the methods section should be restructured to more clearly define the study design.

� The authors are encouraged to align reporting with the Good Reporting of A Mixed Methods Study (GRAMMS) guideline.

� Greater detail is required regarding sampling procedures. Although random sampling is mentioned, the process by which participants were selected is insufficiently described. More explicit reporting would improve transparency, methodological rigor, and reproducibility.

� Clarification is needed regarding inclusion criteria, particularly concerning participant age eligibility and suitability of the psychosocial assessment tools used.

Results

Several important issues require clarification and strengthening.

� The study population appears predominantly female. The authors should discuss whether this may have introduced selection bias or influenced findings.

� One participant was reportedly below 18 years of age. The authors should clarify the participant’s exact age and justify the appropriateness of including minors, particularly in relation to the assessment instruments used.

� It would be valuable to distinguish actual migrant workers from left-behind family members within the analysis. This could provide important subgroup insights regarding differential psychosocial outcomes.

� There are also notable concerns regarding statistical analysis and presentation:

� Table 5 presents life satisfaction and happiness data for midline and endline assessments but excludes baseline data.

� Other psychosocial and mental health outcomes are not comparably presented across all evaluation phases.

� No formal tests of statistical significance were reported to assess changes over time.

Where longitudinal measurements are available, appropriate inferential analyses (e.g., repeated-measures ANOVA or Friedman test) should be considered where methodologically appropriate.

Overall, concerns remain regarding data presentation, statistical analysis, interpretation of associations, subgroup analysis, potential confounding, and overall analytical rigor.

Discussion

The discussion section is currently underdeveloped and would benefit from substantial strengthening.

Specifically:

� The thematic interpretation of qualitative findings requires clearer structure and analytical depth.

� Greater comparison with similar regional and international studies is needed.

� Several important claims are insufficiently supported by literature.

� The discussion would benefit from stronger scientific framing and more critical interpretation of findings in relation to existing evidence.

Conclusion

The conclusions appear somewhat overstated relative to the analyses presented. Conclusions should be more cautiously framed and closely aligned with the actual findings and analytical strength of the study.

4. Minor Concerns

• Syntax and grammatical inconsistencies require attention.

• Several spelling errors were noted.

• The manuscript would benefit from improved flow, transitions, and overall readability.

• Citation formatting and referencing style should be standardised throughout the manuscript.

5. Overall Assessment

This manuscript addresses an important and highly relevant public health issue using an appropriate mixed-methods approach. The topic is timely and potentially valuable for informing mental health and psychosocial interventions among migrant populations and their left-behind families.

However, the manuscript would benefit significantly from improved methodological clarity, more rigorous statistical analysis, stronger integration of baseline and midline findings, enhanced literature engagement, improved scientific writing, and more cautious interpretation of findings.

With substantial revision, the study has the potential to make a meaningful contribution to the literature on migration, mental health, and psychosocial wellbeing in low-resource settings.

**Reviewer #2:**  This manuscript covers original research work about an important public health issue, but needs major improvements before publication.

> Abstract

Please add a description of which analyses you did, this is more important than which software you used.

You write “These outcomes reflect changes observed following …”, but don’t describe the actual changes in the results? Please add.

> Introduction

How many Nepalis return from abroad each year?

It is not clear to me how the EMHPW-MW and PARBARDHAN projects relate to each other. Please also describe what the PARBARDHAN actually did, i.e. the intervention delivered.

You write that “the endline evaluation allowed comparison with baseline and midline indicators” but don’t describe any (quantitative) baseline indicators in the paper?

> Materials and Methods

Please describe how the participants for the quantitative and qualitative parts were selected? Random/purposive sampling?

Where the participants the same in the baseline, midline, and endline evaluations? Or did you sample new persons?

If so, how comparable are your findings across timepoints?

You write that you used data saturation to guide sample size. If this was the case, how did you use saturation? See this reference for guidance:

https://link.springer.com/article/10.1007/s11135-017-0574-8

Please describe whether the questionnaires you used (GHQ-12, HSCL-25, life satisfaction tool, happiness tool) have been validated (content/criterion validity) in your study population (or similar populations)?

What is the “FGD guideline”?

Where were the interviews conducted?

How and where was the quantitative data collected? At facilities? In the community?

Did you analyse means/medians? How are the scores distributed (normal/skewed)?

You state “results were compared to baseline to assess project impact”, where are these results presented?

Please describe which type of thematic analysis you used and how you used it.

> Results

How come nearly all participants in the survey were female?

Please add more information to all your tables, e.g. the number of total participants included in that specific table and which questionnaire is presented in which table.

You write “This represents an increase compared to the baseline prevalence…” and “reflecting a substantial reduction from the baseline prevalence…” but don’t actually present these baseline results. I would suggest that you either present the baseline numbers in the Results (preferably in tables) and thus can show the difference, or you discuss the differences compared with baseline results in the Discussion.

Please state all your themes in the beginning of the “3.2 Qualitative results” section.

In general, I perceive the themes to be underdeveloped in favour of many direct quotes. In qualitative research, the emphasis should be on your interpretation of the data rather than focusing on presenting the “raw” data (= quotes) to the audience. I would like to know more about how and why what you described happened (how did it work?), and for this a more thorough interpretation is often crucial.

The quote starting with “Significant changes have occurred…” is presented as KII in one instance and as IDI in another, which one is correct?

> Discussion

A thorough review of the Discussion and Conclusions is impeded by the severe omissions in the Methods and Results described above.

Please add a paragraph on strengths and limitations of your study.

> References

Most of the references lack journal titles, please add.

> General comment

Please proof-read the article carefully, currently there are multiple typos and grammatical errors.

**Reviewer #3:**  The write up of this paper is good and authors need to work on few areas as highlighted on my attached work documents for clarity and consistency.

This endline evaluation of the PARBARDHAN project addresses an important topic: mental health among Nepali migrant workers and their families. The mixed-methods design is appropriate, but several issues require major revision before publication.

Critical concerns: First, the manuscript reports quantitative data collected in February 2026 (page 5, line 25). As this date is in the future relative to the current submission, a serious error appears present. The authors must clarify whether this is a typographical mistake or explain how data were collected prospectively.

Second, the interpretation of key outcomes is contradictory. Psychological distress increased from 20.4% (baseline) to 34.7% (endline), yet the authors frame this as project effectiveness. While increased awareness may improve reporting, the manuscript fails to convincingly reconcile why a successful intervention would show worsening distress alongside improved anxiety and depression. Similarly, life satisfaction collapsed from ~80% positive at midline to >88% dissatisfied at endline a dramatic reversal attributed vaguely to economic insecurity without demonstrating whether such factors changed during the project period.

Third, the sample is 94% female, but Nepal’s labor migration is predominantly male. Although left-behind families are often female, the near-absence of male returnee migrants severely limits claims about "migrant workers" themselves. This limitation must be explicitly stated.

Minor concerns: Abstract and results sections should report the increase in psychological distress transparently, not merely state that "challenges were highlighted." Table 5 references midline data without providing midline n or statistical comparisons.

Ethical note: No concerns about dual publication or research ethics are evident, provided the data collection date is corrected.

Recommendation: Major revision.

**Reviewer #4:**  Dear Author,

Punctuation and grammar are very important for effective communication and reader comprehension; therefore, greater attention should be paid to their correct use. Secondly, regarding data sampling, it is highly important to clearly indicate the formula that was used in determining the sample size. Lastly, the standard deviation should be appropriately reported using the “±” notation to reflect variability around the mean.

**********

what does this mean?). If published, this will include your full peer review and any attached files.

**Do you want your identity to be public for this peer review?**  If you choose “no”, your identity will remain anonymous but your review may still be made public.

For information about this choice, including consent withdrawal, please see our Privacy Policy

Reviewer #1: No

Reviewer #2: No

Reviewer #3: **Yes:**  Chernor H Jallow

Reviewer #4: **Yes:**  MUHIRE HAVUGIMANA DIEUDONNE

**********

[NOTE: If reviewer comments were submitted as an attachment file, they will be attached to this email and accessible via the submission site. Please log into your account, locate the manuscript record, and check for the action link "View Attachments". If this link does not appear, there are no attachment files.]

**Figure Resubmissions:**

Attachment Submitted filename: My Comments After Reviewing The Paper.docx

Attachment Submitted filename: PGPH-D-26-01439.pdf

# Author comment: Author response to Decision Letter 1

**DOI:** 10.1371/journal.pgph.0006666.r003

30 May 2026

Attachment Submitted filename: response to revioewer endline 30 may.docx

# Editor report: Decision Letter 1

**DOI:** 10.1371/journal.pgph.0006666.r004

**Authors:** Md. Alamgir Hossain

2 Jun 2026

PGPH-D-26-01439R1

Changes in Psychosocial Wellbeing Among Migrant Workers and their families in Nepal: Evidence from a Longitudinal Evaluation of the PARBARDHAN Community-Based Intervention

PLOS Global Public Health

Dear Dr. Himal Gaire,

Thank you for submitting your manuscript to PLOS Global Public Health. After careful consideration, we feel that it has merit but does not fully meet PLOS Global Public Health’s publication criteria as it currently stands. Therefore, we invite you to submit a revised version of the manuscript that addresses the points raised during the review process.

Please submit your revised manuscript by 05/07/2026. If you will need more time than this to complete your revisions, please reply to this message or contact the journal office at globalpubhealth@plos.org. When you're ready to submit your revision, log on to https://www.editorialmanager.com/pgph/ and select the 'Submissions Needing Revision' folder to locate your manuscript file.

Please include the following items when submitting your revised manuscript:

- A letter that responds to each point raised by the editor and reviewer(s). You should upload this letter as a separate file labeled 'Response to Reviewers'.
- A marked-up copy of your manuscript that highlights changes made to the original version. You should upload this as a separate file labeled 'Revised Manuscript with Track Changes'.
- An unmarked version of your revised paper without tracked changes. You should upload this as a separate file labeled 'Manuscript'.

Guidelines for resubmitting your figure files are available below the reviewer comments at the end of this letter.

As the corresponding author, your ORCID iD is verified in the submission system and will appear in the published article. PLOS supports the use of ORCID, and we encourage all coauthors to register for an ORCID iD and use it as well. Please encourage your coauthors to verify their ORCID iD within the submission system before final acceptance, as unverified ORCID iDs will not appear in the published article. *Only*  the individual author can complete the verification step; PLOS staff *cannot*  verify ORCID iDs on behalf of authors.

We look forward to receiving your revised manuscript.

Kind regards,

Md. Alamgir Hossain, M.S.S., M.P.H.

Academic Editor

PLOS Global Public Health

Journal Requirements:

1. If the reviewer comments include a recommendation to cite specific previously published works, please review and evaluate these publications to determine whether they are relevant and should be cited. There is no requirement to cite these works unless the editor has indicated otherwise.

2. Please review your reference list to ensure that it is complete and correct. If you have cited papers that have been retracted, please include the rationale for doing so in the manuscript text, or remove these references and replace them with relevant current references. Any changes to the reference list should be mentioned in the rebuttal letter that accompanies your revised manuscript. If you need to cite a retracted article, indicate the article’s retracted status in the References list and also include a citation and full reference for the retraction notice.

Additional Editor Comments (if provided):

[Note: HTML markup is below. Please do not edit.]

Reviewers' comments:

[NOTE: If reviewer comments were submitted as an attachment file, they will be attached to this email and accessible via the submission site. Please log into your account, locate the manuscript record, and check for the action link "View Attachments". If this link does not appear, there are no attachment files.]

# Author comment: Author response to Decision Letter 2

**DOI:** 10.1371/journal.pgph.0006666.r005

6 Jun 2026

Attachment Submitted filename: Response_to_Reviewers_R2.docx

# Peer review: Decision Letter 2

**DOI:** 10.1371/journal.pgph.0006666.r006

**Authors:** Md. Alamgir Hossain

27 Jul 2026

PGPH-D-26-01439R2

Changes in Psychosocial Wellbeing Among Migrant Workers and their families in Nepal: Evidence from a Longitudinal Evaluation of the PARBARDHAN Community-Based Intervention

PLOS Global Public Health

Dear Dr. Himal Gaire,

Thank you for submitting your manuscript to PLOS Global Public Health. After careful consideration, we feel that it has merit but does not fully meet PLOS Global Public Health’s publication criteria as it currently stands. Therefore, we invite you to submit a revised version of the manuscript that addresses the points raised during the review process.

Please submit your revised manuscript by 05/08/2026. If you will need more time than this to complete your revisions, please reply to this message or contact the journal office at globalpubhealth@plos.org. When you're ready to submit your revision, log on to https://www.editorialmanager.com/pgph/ and select the 'Submissions Needing Revision' folder to locate your manuscript file.

Please include the following items when submitting your revised manuscript:

- A letter that responds to each point raised by the editor and reviewer(s). You should upload this letter as a separate file labeled 'Response to Reviewers'.
- A marked-up copy of your manuscript that highlights changes made to the original version. You should upload this as a separate file labeled 'Revised Manuscript with Track Changes'.
- An unmarked version of your revised paper without tracked changes. You should upload this as a separate file labeled 'Manuscript'.

Guidelines for resubmitting your figure files are available below the reviewer comments at the end of this letter.

As the corresponding author, your ORCID iD is verified in the submission system and will appear in the published article. PLOS supports the use of ORCID, and we encourage all coauthors to register for an ORCID iD and use it as well. Please encourage your coauthors to verify their ORCID iD within the submission system before final acceptance, as unverified ORCID iDs will not appear in the published article. *Only*  the individual author can complete the verification step; PLOS staff *cannot*  verify ORCID iDs on behalf of authors.

We look forward to receiving your revised manuscript.

Kind regards,

Md. Alamgir Hossain, M.S.S., M.P.H.

Academic Editor

PLOS Global Public Health

**Journal Requirements:**

If the reviewer comments include a recommendation to cite specific previously published works, please review and evaluate these publications to determine whether they are relevant and should be cited. There is no requirement to cite these works unless the editor has indicated otherwise.

Please review your reference list to ensure that it is complete and correct. If you have cited papers that have been retracted, please include the rationale for doing so in the manuscript text, or remove these references and replace them with relevant current references. Any changes to the reference list should be mentioned in the rebuttal letter that accompanies your revised manuscript. If you need to cite a retracted article, indicate the article’s retracted status in the References list and also include a citation and full reference for the retraction notice.

**Additional Editor Comments (if provided):**

[Note: HTML markup is below. Please do not edit.]

**Reviewers' comments:**

The comments provided by previous reviewers have been addressed.

The manuscript highlights a critical issue related to mental health and migrant workers and their families. The data collected is adequate and provides insights into important aspects of the lives of migrant workers and their families.

A few conceptual issues remain:

1. Title and study population. Although the title has been revised, the title "Changes in Psychosocial Wellbeing Among Migrant Workers and their families in Nepal: Evidence from a Longitudinal Evaluation of the PARBARDHAN Community-Based Intervention" appears to place greater emphasis on migrant workers themselves. However, the study sample consists predominantly of family members rather than migrant workers, which may be somewhat misleading, particularly in the title. This is particularly important because the analysis does not present the data separately for these two groups.

2. Interpretation of changes over time. The authors appropriately acknowledge the limitation of having a different population at endline due to the repeated cross-sectional study design. However, this limitation also affects the interpretation of changes observed between baseline, midline, and endline. Since the endline participants are not the same individuals assessed previously, improvements cannot be directly attributed to changes within the study population. It may therefore be more appropriate to frame the manuscript primarily as an endline evaluation, while using the baseline and midline findings for contextual comparison rather than implying longitudinal improvement. For example, rather than suggesting a decrease in depression and anxiety over time, the manuscript could state that previous evaluations reported higher prevalence estimates, whereas the endline evaluation found lower proportions. This framing would more accurately reflect the study design and strengthen the manuscript's conclusions.

Overall, this is a well-written manuscript addressing an important topic. The remaining issues are primarily related to the framing and interpretation of the findings and can be addressed through revisions.

[NOTE: If reviewer comments were submitted as an attachment file, they will be attached to this email and accessible via the submission site. Please log into your account, locate the manuscript record, and check for the action link "View Attachments". If this link does not appear, there are no attachment files.]

**Figure Resubmissions:**

# Author comment: Author response to Decision Letter 3

**DOI:** 10.1371/journal.pgph.0006666.r007

3 Aug 2026

Attachment Submitted filename: Response_to_Editor.docx

# Peer review: Decision Letter 3

**DOI:** 10.1371/journal.pgph.0006666.r008

**Authors:** Md. Alamgir Hossain

1 Sep 2026

PGPH-D-26-01439R3

Changes in Psychosocial Wellbeing Among Families and Communities of Migrant Workers in Nepal: Evidence from a Endline Evaluation of the PARBARDHAN Community-Based Intervention

PLOS Global Public Health

Dear Dr. Himal Gaire,

Thank you for submitting your manuscript to PLOS Global Public Health. After careful consideration, we feel that it has merit but does not fully meet PLOS Global Public Health’s publication criteria as it currently stands. Therefore, we invite you to submit a revised version of the manuscript that addresses the points raised during the review process.

Please submit your revised manuscript by 05/10/2026. If you will need more time than this to complete your revisions, please reply to this message or contact the journal office at globalpubhealth@plos.org. When you're ready to submit your revision, log on to https://www.editorialmanager.com/pgph/ and select the 'Submissions Needing Revision' folder to locate your manuscript file.

Please include the following items when submitting your revised manuscript:

- A letter that responds to each point raised by the editor and reviewer(s). You should upload this letter as a separate file labeled 'Response to Reviewers'.
- A marked-up copy of your manuscript that highlights changes made to the original version. You should upload this as a separate file labeled 'Revised Manuscript with Track Changes'.
- An unmarked version of your revised paper without tracked changes. You should upload this as a separate file labeled 'Manuscript'.

Guidelines for resubmitting your figure files are available below the reviewer comments at the end of this letter.

As the corresponding author, your ORCID iD is verified in the submission system and will appear in the published article. PLOS supports the use of ORCID, and we encourage all coauthors to register for an ORCID iD and use it as well. Please encourage your coauthors to verify their ORCID iD within the submission system before final acceptance, as unverified ORCID iDs will not appear in the published article. *Only*  the individual author can complete the verification step; PLOS staff *cannot*  verify ORCID iDs on behalf of authors.

We look forward to receiving your revised manuscript.

Kind regards,

Md. Alamgir Hossain, M.S.S., M.P.H.

Academic Editor

PLOS Global Public Health

**Journal Requirements:**

If the reviewer comments include a recommendation to cite specific previously published works, please review and evaluate these publications to determine whether they are relevant and should be cited. There is no requirement to cite these works unless the editor has indicated otherwise.

Please review your reference list to ensure that it is complete and correct. If you have cited papers that have been retracted, please include the rationale for doing so in the manuscript text, or remove these references and replace them with relevant current references. Any changes to the reference list should be mentioned in the rebuttal letter that accompanies your revised manuscript. If you need to cite a retracted article, indicate the article’s retracted status in the References list and also include a citation and full reference for the retraction notice.

**Additional Editor Comments (if provided):**

[Note: HTML markup is below. Please do not edit.]

Reviewers' comments:

Reviewer's Responses to Questions

**Comments to the Author**

Reviewer #6: All comments have been addressed

Reviewer #7: (No Response)

**********

publication criteria?

Reviewer #6: Yes

Reviewer #7: No

**********

3. Has the statistical analysis been performed appropriately and rigorously?

Reviewer #6: Yes

Reviewer #7: No

**********

4. Have the authors made all data underlying the findings in their manuscript fully available (please refer to the Data Availability Statement at the start of the manuscript PDF file)?

The PLOS Data policy

Reviewer #6: Yes

Reviewer #7: No

**********

5. Is the manuscript presented in an intelligible fashion and written in standard English?

Reviewer #6: Yes

Reviewer #7: Yes

**********

**Reviewer #6:**  The authors have addressed previous feedback. However, deidentified data should be made accessible as noted in the submission of the authors when PLOS makes the request. The data set should be handy ahead of such request.

**Reviewer #7** : I thank the editor and the authors for the opportunity to review this manuscript. The study addresses a relevant public health problem related to the mental health and psychosocial well-being of migrant workers and their families in Nepal, within the context of the endline evaluation of the PARBARDHAN project. The topic is relevant to the scope of PLOS Global Public Health, and the use of quantitative and qualitative methods has the potential to generate useful information for the planning of community interventions.

However, after a detailed evaluation of the manuscript, I consider that the current version requires a major and substantial revision before it can be considered for publication. The main concerns do not correspond solely to aspects of editorial presentation, but rather to issues related to the coherence between the study design and the inferences made, the comparability of the different evaluation rounds, the definition of the study population, the statistical strategy, the interpretation of the results, the depth of the Introduction and Discussion, and the documentation of ethical aspects.

The central problem running throughout the manuscript is that different sections use expressions that suggest program effectiveness or impact, whereas the described design corresponds to a cross-sectional evaluation and the different evaluation rounds do not appear to correspond to longitudinal follow-up of the same individuals. Therefore, it is necessary to carefully revise the way the results are presented and discussed in order to avoid causal inferences that cannot be supported by the design.

PLEASE REVIEW THE ATTACHED FILE

**********

what does this mean?). If published, this will include your full peer review and any attached files.

**Do you want your identity to be public for this peer review?**  If you choose “no”, your identity will remain anonymous but your review may still be made public.

For information about this choice, including consent withdrawal, please see our Privacy Policy

Reviewer #6: **Yes:**  Olubunmi Adedoyin OjeladeThe autho

Reviewer #7: No

**********

[NOTE: If reviewer comments were submitted as an attachment file, they will be attached to this email and accessible via the submission site. Please log into your account, locate the manuscript record, and check for the action link "View Attachments". If this link does not appear, there are no attachment files.]

**Figure Resubmissions:**

Attachment Submitted filename: MANUSCRIPT EVALUATION.pdf

# Author comment: Author response to Decision Letter 4

**DOI:** 10.1371/journal.pgph.0006666.r009

15 Sep 2026

Attachment Submitted filename: Response to the reviewers.docx

# Editor report: Decision Letter 4

**DOI:** 10.1371/journal.pgph.0006666.r010

**Authors:** Md. Alamgir Hossain

21 Sep 2026

Mental Health and Psychosocial Wellbeing Among Beneficiaries of a Community-Based Intervention In: Findings from a Cross-Sectional Endline Evaluation of the PARBARDHAN Project

PGPH-D-26-01439R4

Dear Dr. Himal Gaire,

We are pleased to inform you that your manuscript 'Mental Health and Psychosocial Wellbeing Among Beneficiaries of a Community-Based Intervention In: Findings from a Cross-Sectional Endline Evaluation of the PARBARDHAN Project' has been provisionally accepted for publication in PLOS Global Public Health.

Before your manuscript can be formally accepted you will need to complete some formatting changes, which you will receive in a follow up email. A member of our team will be in touch with a set of requests.

Please note that your manuscript will not be scheduled for publication until you have made the required changes, so a swift response is appreciated.

IMPORTANT: The editorial review process is now complete. PLOS will only permit corrections to spelling, formatting or significant scientific errors from this point onwards. Requests for major changes, or any which affect the scientific understanding of your work, will cause delays to the publication date of your manuscript.

If your institution or institutions have a press office, please notify them about your upcoming paper to help maximize its impact. If they'll be preparing press materials, please inform our press team as soon as possible -- no later than 48 hours after receiving the formal acceptance. Your manuscript will remain under strict press embargo until 2 pm Eastern Time on the date of publication. For more information, please contact globalpubhealth@plos.org.

Thank you again for supporting Open Access publishing; we are looking forward to publishing your work in PLOS Global Public Health.

Best regards,

Md. Alamgir Hossain, M.S.S., M.P.H.

Academic Editor

PLOS Global Public Health

***********************************************************

Reviewer Comments (if any, and for reference):
